# New ionization tags based on the structure of the 5-azoniaspiro[4.4]nonyl tag for a sensitive peptide sequencing by mass spectrometry

**DOI:** 10.1007/s00216-017-0771-2

**Published:** 2017-12-06

**Authors:** Bartosz Setner, Zbigniew Szewczuk

**Affiliations:** Faculty of Chemistry, University of Wrocław, F. Joliot-Curie 14, 50383 Wrocław, Poland

**Keywords:** Derivatization of peptides, Peptide sequencing, Ionization tag, Tandem mass spectrometry

## Abstract

Quaternary ammonium salts (QAS), both linear and bicyclic, are often utilized to improve the mass spectrometry (MS) analysis of peptides by fixing a permanent positive charge on the analyzed molecule. However, during collision-induced dissociation (CID) experiments, QAS undergo unwanted side reactions—Hofmann elimination as well as a tertiary amine loss— rendering the data interpretation complicated. In this work, we present 2-thia- and 2-oxa-5-azoniaspiro[4.4]nonyl groups as heterocyclic derivatives of the highly stable ionization group, 5-azoniaspiro[4.4]nonyl, for a sensitive peptide analysis by MS. Due to the permanent positive charge, labeled peptides are characterized by enhanced ionization efficiency during electrospray mass spectrometry (ESI-MS) conditions. Moreover, interpretation of the CID fragmentation of labeled peptides is facilitated since a series of generated fragmentation ions enable a complete sequence coverage. Introduction of a heteroatom into the 5-azoniaspiro[4.4]nonyl scaffold allows for liberation of a stable reporter ion which could be used in selected reaction monitoring (SRM)-targeted quantification experiments. Additionally, we synthesized a deuterated analog of the tag for LC-SRM-targeted quantitative analysis. The obtained results indicate the general usefulness of the proposed heterocyclic quaternary ammonium ionization tag for sequencing and quantification of peptides.

**Electronic supplementary material:**

The online version of this article (10.1007/s00216-017-0771-2) contains supplementary material, which is available to authorized users.

**Graphical abstract:**
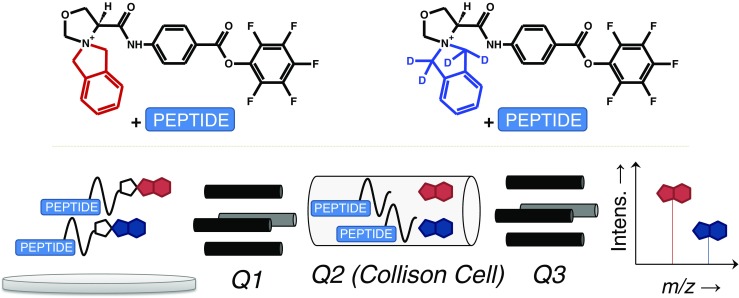
New reagents based on the structure of the 5-azoniaspiro[4.4]nonyl tag for peptide analysis by tandem mass spectrometry

## Introduction

Recently made progress in both instrumentation as well as methodology of mass spectrometry (MS) makes it a method of choice for analysis of complex peptide mixtures [2]. In proteomics studies, a commonly utilized strategy is a shotgun approach. In this technique, protein samples are enzymatically digested into peptides which are then analyzed by MS [3]. However, only qualitative information about the peptides present in the prepared samples could be obtained by this approach. The simplest method for MS-based peptides quantification strategy is based on eXtracted Ion Chromatograms (XIC) derived from LC-MS measurements. In this approach a peak height or a peak area are used to determine the analyte abundance. However, due to a limited sensitivity as well as a poor reproducibility of LC-MS measurements, a new strategy—selected reaction monitoring (SRM) was introduced [4].

SRM is a non-scanning technique exploiting the unique capabilities of a triple quadrupole for quantitative analysis. In the SRM experiment, the first quadrupole (Q1) and the third quadrupole (Q3) are set to filter off any unwanted signals and thus are set to specifically selected *m/z* values. In Q1, *m/z* corresponds to the peptide ion, and in Q3, *m/z* is set at a specific fragment ion of the peptide. The second quadrupole (Q2) in this case serves as a collision cell [5]. The two levels of *m/z* selections with a narrow *m/z* window result a in a high selectivity. Due to non-scanning nature of this technique, sensitivity increases from one to two orders of magnitude compared with a full-scan mode. Furthermore, SRM offers a linear response over a wide dynamic range up to five orders of magnitude. This allows for detection of low abundance peptides (typically <10^−15^ mol) [4, 6]). However, to properly quantify peptides using SRM technique, the internal standard is needed [7, 8]. Absolute Quantification (AQUA) method, first introduced by Barr and coworkers [9] and further extended by Gygi and coworkers [10], used a set of synthetic peptides that incorporated stable isotopes. Obtained standards are chemically identical with naturally occurring peptides except isotopic composition. Although AQUA approach offers simplicity, accuracy, sensitivity, and ability for measurements of multiple peptides in one run, preparation of the standards is highly expensive and thus can limit the number of analyzed peptides in a final sample [5].

Chemical derivatization of target peptide standards could be used as an alternative approach. In stabile-isotope dimethyl labeling approach, all primary amines of peptides (N-termini as well as lysine side chain) are labeled by formaldehyde in combination with the reduction of the initially formed shift base using cyanborohydride [8]. Utilization of different isotopomers of the reagents results in doublets or triplets in the spectra, which offers direct readout of relative protein abundances in the sample [11, 12]. Commercially available mTRAQ reagent is a non-isobaric extension of the iTRAQ reagent [13]. Peptides derivatized with different mTRAQ reagents have identical retention times and ionization efficiencies but different masses due to isotopic composition. SRM quantification experiment is performed using the non-isobaric, sequence specific ions, instead of reporter ions, for a higher specificity in complex mixtures. Similar approach is offered by isotopic *N*,*N*-dimethyl leucine (iDiLeu) reagents [14]. However, both reagents based on tertiary amines do not provide a significant gain in ionization efficiency. Therefore, these tags may not be suitable for a sub-femtomol peptide analysis.

Well-known method to increase the ionization efficiency and thus to lower the detection limit is derivatization with a permanent positive charge [15]. Recently, we developed an efficient synthetic strategy for preparation of peptide conjugates with quaternary ammonium salts [16, 17]. Nano-LC-SRM analysis of the obtained peptide conjugates confirmed that derivatization resulting in a positive charge significantly increases the detection limit of derivatized peptides [18]. Recently, we used triphenylpyrylium salt for peptide analysis [19] as well as developed a highly stable ionization tag—5-azoniaspiro[4.4]nonyl which does not undergo intramolecular fragmentation during MS/MS experiment [20]. Thus, the proposed reagent can be applied in a straightforward peptide sequencing. On the other hand, decreasing the stability of the azoniaspiro tag can be used to selectively liberate a characteristic reporter ion for peptide analysis.

Here, we report a new reagent based on the structure of the 5-azoniaspiro[4.4]nonyl tag. The proposed tag contains the 5-azoniaspiro[4.4]nonyl scaffold with a heteroatom—sulfur or oxygen at position 2, which allows for easy liberation of the reporter ion during MS/MS conditions, thus enabling a straightforward peptide analysis by SRM technique.

## Materials and methods

### Chemicals

All reagents for peptide synthesis were purchased from Iris Biotech GmbH (Marktredwitz, Germany), Fluorochem (Hadfield, Derbyshire, UK), or Bachem AG (Bubendorf, Switzerland). All salts, solvents, buffers, as well as myoglobin from equine heart ≥ 90% (SDS-PAGE) and *o*-xylene-(dimethyl-d_6_) (98 at.% D) were purchased from Sigma Aldrich (St. Louis, MO, USA). All solvents and reagents were used as supplied. Formaldehyde[d_2_] (~ 20 wt.% in D_2_O; 98 at.% D) was purchased from Cambridge Isotope Laboratories (Tewksbury, MA, USA). Trypsin/Lys-C Mix, Mass Spec Grade was purchased from Promega (Madison, WI, USA).

### Heterocyclic quaternary ammonium salt formation

The synthesis of model tetrapeptides was performed on the MBHA-Rink Amide resin (loading, 0.55 mM/g). All syntheses were performed manually in polypropylene syringe reactors (Intavis AG, Köln, Germany) equipped with polyethylene filters, according to a standard Fmoc (9-fluorenylmethoxycarbonyl) solid-phase synthesis procedure. The Fmoc group was removed from the N-terminal amino group of peptides synthesized on the Rink Amide MBHA resin with 25% solution of piperidine in DMF. The peptidyl-resin was coupled with Fmoc-thiazolidine-4-carboxylic acid (Fmoc-Thz-OH) or Fmoc-oxazolidine-4-carboxylic acid (Fmoc-Oxa-OH) in the presence of HATU/Oxyma Pure/DIPEA (3 equiv. each) for 2 h. Next, fivefold excess of 1,4-dibromobutane or *α*,*α*′-dibromo-*o*-xylene and DIPEA were added and the reaction was carried out for additional 24 h. The derivatized peptides were cleaved from the resin simultaneously with the side chain deprotection using a solution of TFA/H_2_O/TIS (95:2.5:2.5, *v*/*v*/*v*) at room temperature for 2 h. All quaternary ammonium groups were stabile at those conditions. The quaternary ammonium salt (QAS) peptides were purified by RP-HPLC.

### Model tryptic peptides synthesis

Model synthetic tryptic peptides were synthesized on the preloaded Wang resins (Fmoc-Lys(Boc)-Wang resin; loading, 0.72 mM/g) according to a standard Fmoc solid-phase synthesis procedure (Table 1).

**Table 1:** Model synthetic tryptic peptides examined in this study

Peptide sequence	*m/z* found	*m/z* calculated	Retention time (min)
LVTDLTK	789.471	798.470 [M+H]^+^	9.1
LVNELTEFAK	1163.630	1163.629 [M+H]^+^	14.7
HGTVVLTALGGILK	1378.857	1378.841 [M+H]^+^	20.9
	689.925	689.924 [M+2H]^2+^	
LFTGHPETLEK	1271.677	1271.663 [M+H]^+^	13.2
	636.336	636.335 [M+2H]^2+^	

### Synthesis of the heterocyclic quaternary ammonium salt derivatization reagent

The synthesis of the derivatization reagent based on heterocyclic quaternary ammonium salt was performed on a preloaded 2-chlorotrityl resin (4-amino benzoic acid-2CT resin; loading, 0.40 mM/g) manually in polypropylene syringe reactors (Intavis AG, Köln, Germany) equipped with polyethylene filters, according to a standard Fmoc (9-fluorenylmethoxycarbonyl) solid-phase synthesis procedure. Heterocyclic quaternary ammonium salt, namely, 2-oxa-benzo-5-azoniaspiro[4.4]nonylcarboxyl group was obtained as described in the section “Heterocyclic quaternary ammonium salt formation.” Obtained compound was cleaved from the resin using a solution of TFA/H_2_O/TIS (95:2.5:2.5, *v*/*v*/*v*) at room temperature for 2 h. The product was purified by RP-HPLC.

The synthesis of the pentafluorophenyl esters was performed by dissolving 0.01 mmol of the appropriate heterocyclic quaternary ammonium salt derivatization reagent in 100 μL of dry DMF and adding 0.8 μL (1 equiv.) of pyridine and 1.7 μL (1 equiv.) of pentafluorophenyl trifluoroacetate. Mixing was performed at room temperature for 1 h, and the solution was stored at − 8 °C until needed for a future labeling.

### Purification

All derivatized peptides were purified by the PR-HPLC using a Thermo Separation Products system (Waltham, MA, USA) with UV detection (210 nm) on a YMC-Pack ODS-AQ12S05-2546WT column (250 × 4.6 mm, 5 μm), with a gradient elution of 0–80% B in A (flow rate, 1 mL/min). The main peak, corresponding to the QAS-peptide derivative or a peptide, was collected and the fraction was lyophilized.

### Peptide labeling

The 50-μL aliquot (from 1 mg/1 mL of triethylammonium bicarbonate (TEAB) (1 M, pH 8.5) solution of a peptide) was taken for labeling. One hundred microliters of acetonitrile was added to each vial with a buffer solution of a peptide, and 10 μL of previously prepared DMF solution of heterocyclic QAS derivatization reagent pentafluorophenyl ester was added (~ 10-molar excess of reagent over primary amine). Mixing was performed at room temperature for 1 h and then 100 μL of HCOOH and 500 μL of water were added and incubation was prolonged for 30 min. Labeled samples were than dried under SpeedVac (CentriVap, Labconco, Kansas City, MO, USA).

### Myoglobin tryptic digest

For the trypsin/rLys-C digestion of myoglobin, 1 mg of myoglobin from equine heart ≥ 90% (SDS-PAGE) lyophilized powder was dissolved in 100 μL of TEAB (1 M, pH 8.5). Ten microliters of trypsin/rLys-C stock solution (20 μg in 500 μL of resuspension buffer) was added to reach the enzyme:substrate mass ratio of 1:25. The reaction mixture was incubated for 48 h at 36 °C. The resulting digest was lyophilized and used for the labeling. Lyophilized myoglobin digest was dissolved in 1 mL of TEAB buffer (1 M, pH 8.5). Fifty microliters of the aliquot were then taken for labeling (Table 2).

**Table 2:** List of peptides from myoglobin tryptic digest

Peptide sequence	Charge				RT (min)
	1+	2+	3+	4+	
	*m/z* found (calculated)				
YLEFISDAIIHVLHSK	n.o.	943.010 (943.014)	629.009 (629.012)	472.006 (472.010)	26.7
GHHEAELKPLAQSHATK	n.o.	n.o.	618.618 (618.658)		25.6
GLSDGEWQQVLNVWGK	n.o.	908.448 (908.454)	605.972 (605.972)		25.6
VEADIAGHGQEVLIR	n.o.	803.924 (803.931)	536.288 (536.289)		18.1
HPGDFGADAQGAMTK	n.o.	751.837 (751.838)	n.o.		15.0
HGTVVLTALGGILK	n.o.	689.923 (689.924)	n.o.		22.7
LFTGHPETLEK	n.o.	636.333 (636.335)	424.558 (424.559)		15.7
ALELFR	748.438 (748.435)	n.o.	n.o.		20.2
TEAEMK	n.o.	n.o.	n.o.		
ASEDLK	n.o.	n.o.	n.o.		
ELGFQG	650.318 (650.314)	n.o.	n.o.		17.0
NDIAAK	n.o.	n.o.	n.o.		

n.o., not observed

### Mass spectrometry

Mass spectrometric experiments were performed on a Bruker micrOTOF-Q mass spectrometer (Bruker Daltonics, Bremen, Germany) and on a Fourier transform ion cyclotron resonance (FT-ICR) Apex-Qe Ultra 7 T instrument (Bruker Daltonics, Bremen, Germany) equipped with an ESI source. Analyte solutions (~ 1 μg of QAS-derivatized peptide in 1 mL of 50:50 acetonitrile–water mixture containing 0.1% HCOOH or ~ 100 μg of peptide in 1 mL of 50:50 acetonitrile–water mixture containing 0.1% HCOOH) were pumped at a rate of 300 μL/h. The instrument was operated in the positive ion mode and calibrated before each analysis with the Tunemix™ mixture (Agilent Technologies, Santa Clara, USA) in quadratic method. In the MS/MS experiments, the singly charged [M]^+^ precursor ions as well as doubly charged [M+H]^2+^ precursor ions were selected on the quadrupole and subsequently fragmented in the collision cell. The collision voltage was optimized for the best fragmentation pattern. Average number of scans for ESI-MS/MS spectra was 20. For the MS as well as MS/MS spectra analysis, a Bruker Compass DataAnalysis 4.0 software was used.

SRM experiments were performed on an LCMS-8050 Triple Quadrupole Liquid Chromatograph Mass Spectrometer, equipped with an ESI source, coupled to UHPLC Nexera X2 (Shimadzu, Duisburg, Germany) on a Vertex Plus Eurospher II 100-2 C18 A column (50 × 2.0 mm, 2 μm) (KNAUER Wissenschaftliche Geräte GmbH, Berlin, Germany), with a gradient elution of 2–95% B in A (A = 0.1% formic acid in water; B = 0.1% formic acid in acetonitrile) for 10 min (flow rate 0.2 mL/min). The instrument was operated in the positive ion mode. For the SRM chromatogram analysis, a LabSolutions software was used.

The LC-MS analysis was performed using an UltiMate 3000 HPLC (Thermo Fisher Scientific, Waltham, MA, USA) system coupled to a Bruker maXis Impact mass spectrometer (Bruker Daltonics, Bremen, Germany). For separation, a Vertex Plus Eurospher II 100-2 C18 A column (50 × 2.0 mm, 2 μm) (KNAUER Wissenschaftliche Geräte GmbH, Berlin, Germany) was used, with a gradient elution of 0% B for 2 min (A = 0.1% formic acid in water; B = 0.1% formic acid in acetonitrile) and then 0–5% B in A for 3 min, then 5–95% B in A for 40 min (flow rate, 0.1 mL/min; column temperature, 30 °C). The injection volume was 2 μL. For the LC-MS chromatogram analysis, a Bruker Compass DataAnalysis 4.0 software was used.

### NMR spectroscopy

^1^H NMR and ^13^C NMR spectra were recorded on high-field spectrometers Bruker Avance III 500 MHz and Bruker Avance III 600 MHz equipped with a broadband inverse gradient probeheads. Spectra were referenced to the residual solvent signal ([D]chloroform, 7.26 ppm and [^13^C]chloroform, 77.16 ppm; [D]acetonitrile, 1.94 ppm and [^13^C]acetonitrile, 1.32 ppm; [D]water, 4.79 ppm; [^13^C]trifluoroacetic acid, 116.60 and 164.20 ppm).

### Non-polar surface area

The non-polar surface area (NPSA) for the peptide was calculated by summing the non-polar surface areas of the individual amino acid residues [21]. A value for the tag was estimated using basic geometry along with bond lengths and Van der Waals radii.

## Results and discussion

**Fig. 1:**
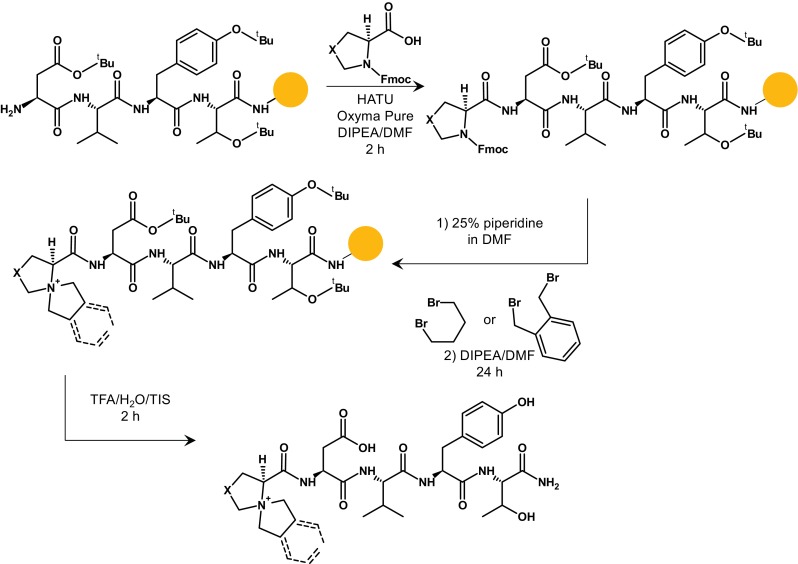
Synthesis of the model QAS-peptide derivatives on the solid support. DIPEA, *N*,*N*-diisopropylethylamine, DMF, *N*,*N*-dimethylformamide, TFA, trifluoroacetic acid, TIS, triisopropylsilane, *X*-sulfur or oxygen atom. Yellow ball represents polystyrene solid support

The aim of our research was to develop a new quaternary ammonium salt ionization tag capable of releasing an intense reporter ion for a sensitive peptide analysis and quantification. We chemically modified the 5-azoniaspiro[4.4]nonyl scaffold, previously developed by us [20], to liberate a stabile fragment ion with a quaternary ammonium nitrogen atom during collision-induced dissociation (CID) experiment. Previously, Bąchor et al. observed that polyethylene glycol-modified QAS peptide conjugates undergo internal fragmentation within the bicyclic moiety during CID experiment [22]. Based on this finding, we designed and synthesized on the solid support a series of model tetrapeptides containing N-terminal heterocyclic analogue of the 5-azoniaspiro[4.4]nonyl as well as benzo-5-azoniaspiro[4.4]nonyl groups (Fig. 1.). The QAS groups were also introduced to the side chain of the C-terminal lysine residue. For the synthesis of the heterocyclic QAS groups, we used two commercially available proline derivatives, namely, thiazolidine-4-carboxylic acid (Thz) and oxazolidine-4-carboxylic acid (Oxa). The QAS peptide sequences and molecular masses of all the synthesized QAS peptides are presented in Table S1 (see Electronic supplementary material (ESM)).

Selected QAS peptides were analyzed using the NMR technique. The analysis of the ^1^H and ^13^C spectra as well as 2D spectra (COSY, HMQC, HMBC, and NOESY) confirmed the presence of the heterocyclic quaternary ammonium scaffolds at the N-terminus as well as the amino acid sequences of obtained compounds (see Figs. S52, S53, S54, S55, S56, and S75 in the ESM).

The differences in electrospray response between various analytes often depend on their hydrophobicity [23]. Previously, Muddiman and coworkers used hydrophobic moieties (such as *N*-ethylmaleimide and iodoacetamide derivatives) to increase the electrospray response of peptides [24, 25]. Reported results show that peptides with the NPSA < 500 Å^2^ displayed an increase in the MS response when modified. On the other hand, peptides with NPSA > 700 Å^2^ show a decreased MS response when modified with a hydrophobic tag. This indicates that there is an optimal NPSA zone where the MS response is optimal. Peptides labeled by heterocyclic QAS ionization tags show intense signals in MS spectra which correspond to non-protonated singly charged ions [M]^+^. Derivatization of model synthetic peptide LVTDLTK (calculated NPSA: 810 Å^2^ [21]) with a positive charge, based on the heterocyclic analogue of the benzo-5-azoniaspiro[4.4]nonyl group (estimated NPSA of the tag, 235 Å^2^), provided up to fivefold ionization amplification compared with the unlabeled synthetic model peptide (see Figs. S15 and S16 in the ESM). In spite of NPSA of the peptide being in the range of > 700 Å^2^ for which no ionization enhancement should be observed according to the literature, a permanent positive charge and increased hydrophobicity of the peptide + tag analyte improve the ESI response [26]. However, ionization improvement obtained for a labeled peptide does not match previously reported tenfold ionization enhancement of the 5-azoniaspiro[4.4]nonyl group [20]. This decrease in ionization enhancement of charged tagged peptides could be caused by a more hydrophilic nature of the heterocyclic azoniaspiro group, containing the sulfur or oxygen atom at position 2 [27]. Thus, heterocyclic tags containing the hydrophobic benzene ring provide a better ionization improvement as judged from MS experiments.

**Fig. 2:**
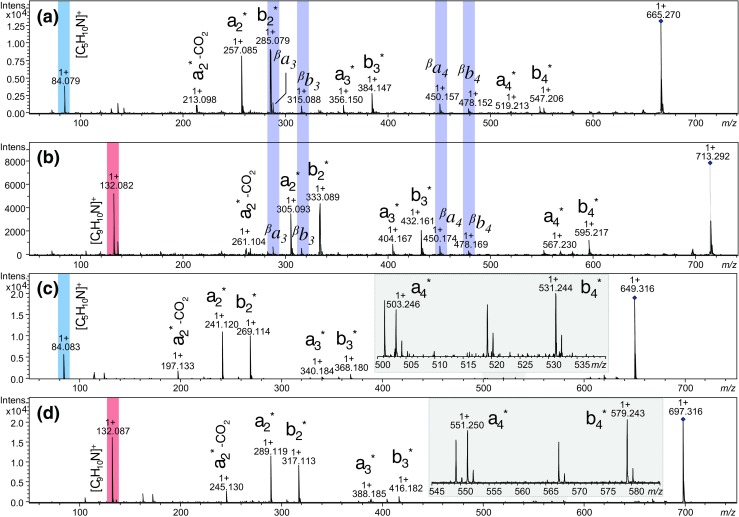
**a** ESI-MS/MS spectrum of the ASN^Thz+^-CO-DVYT-NH_2_, *m/z* 665.270 (collision energy, 25 V); **b** ESI-MS/MS spectrum of the BASN^Thz+^-CO-DVYT-NH_2_, *m/z* 713.292 (collision energy, 25 V); **c** ASN^Oxa+^-CO-DVYT-NH_2_, *m/z* 649.316 (collision energy, 25 V); **d** BASN^Oxa+^-CO-DVYT-NH_2_, *m/z* 697.316 (collision energy, 25 V). Asterisk is added to denote the presence of the derivative moiety that represents a charged (non-protonated) fragment. Diamond symbol denotes the parent peaks

All synthesized model compounds were subjected to high-resolution ESI-MS/MS analysis (selected MS/MS spectra are given in Fig. 2). MS/MS spectra were collected for singly charged, non-protonated [M]^+^ ions. For the model tetrapeptides conjugated with sulfur containing heterocyclic analogue of 5-azoniaspiro[4.4]nonyl and benzo-5-azoniaspiro[4.4]nonyl scaffolds at the N-terminus (see Fig. 2a, b as well as Figs. S1, S2, S3, and S4 in the ESM), we observed the presence of the characteristic series of N-terminal fragments (*a*- as well as *b*-type ions), which facilitates the sequencing analysis of the charged derivatized peptides. However, due to the presence of the heteroatom in the structure of QAS in all obtained ESI-MS/MS spectra, we observed additional *a*- and *b*-type ions (present in the MS/MS spectra as ^β^ *a*_i_ as well as ^β^ *b*_i_, marked violet in Fig. 2), which are derived from liberating the C_4_H_7_N fragment (calc. monoisotopic mass of C_4_H_7_N is 69.057 Da) from the 2-thia-5-azoniaspiro[4.4]nonyl group and C_8_H_7_N fragment (calc. monoisotopic mass of C_8_H_7_N is 117.057 Da) from the 2-thia-benzo-5-azoniaspiro[4.4]nonyl group (proposed ion fragments structures are present in the ESM, Scheme S3). Moreover, an intense signal at *m/z* 84 (calc. for C_5_H_10_N^+^ 84.080) of the ion liberated from the 2-thia-5-azoniaspiro[4.4]nonyl group (marked blue in Fig. 2.) and at *m/z* 132 (calc. for C_9_H_10_N^+^ 132.080) of the ion liberated from the 2-thia-benzo-5-azoniaspiro[4.4]nonyl group (marked red in Fig. 2.) are present in the obtained MS/MS spectra (proposed ion fragment structures are present in the ESM, Scheme S4).

The ESI-MS/MS analysis of the model tetrapeptides conjugated with the oxygen containing heterocyclic analogue of 5-azoniaspiro[4.4]nonyl and benzo-5-azoniaspiro[4.4]nonyl scaffolds (see Fig. 2c, d as well as Figs. S5, S6, S7, and S8 in the ESM) unveils the presence of the *a*- and *b*-type ions. Although in this case, none of the previously registered unwanted signals were present in the spectra which facilitate a straightforward sequence interpretation. This phenomenon could be explained basing on the unique ability of oxygen-containing heterocyclic QAS to liberate formaldehyde molecule from the tag, which facilitates reporter ion formation. Furthermore, intense signals at *m/z* 84 (marked blue in Fig. 2.) and *m/z* 132 (marked red in Fig. 2.) were noticed.

We synthesized model tryptic peptide LVTDLTK from bovine serum albumin, which was modified at the ε-amine group of the C-terminal lysine residue with heterocyclic 5-azoniaspiro[4.4]nonyl and benzo-5-azoniaspiro[4.4]nonyl groups. The ESI-MS/MS analysis was performed for both [M]^+^ as well as [M+H]^2+^ (singly protonated) ions. In the ESI-MS/MS spectra of [M]^+^ QAS peptides modified with both heterocyclic QAS moieties (see Figs. S9, S10, S11, and S12 in the ESM), we observed very low intense signals. Therefore, we introduced a linker, 4-aminobenzoic acid (4-Abz), to separate the peptide chain from the heterocyclic QAS. Additionally, introduction of hydrophobic moiety to the tag structure should contribute to ionization enhancement of the QAS [28]. The ESI-MS/MS analysis of the singly [M]^+^ as well as doubly charged [M+H]^2+^ ions shows a significant improvement in the case of both signal numbers as well as ions intensity (see Figs. S13 and S14 in the ESM).

**Fig. 3:**
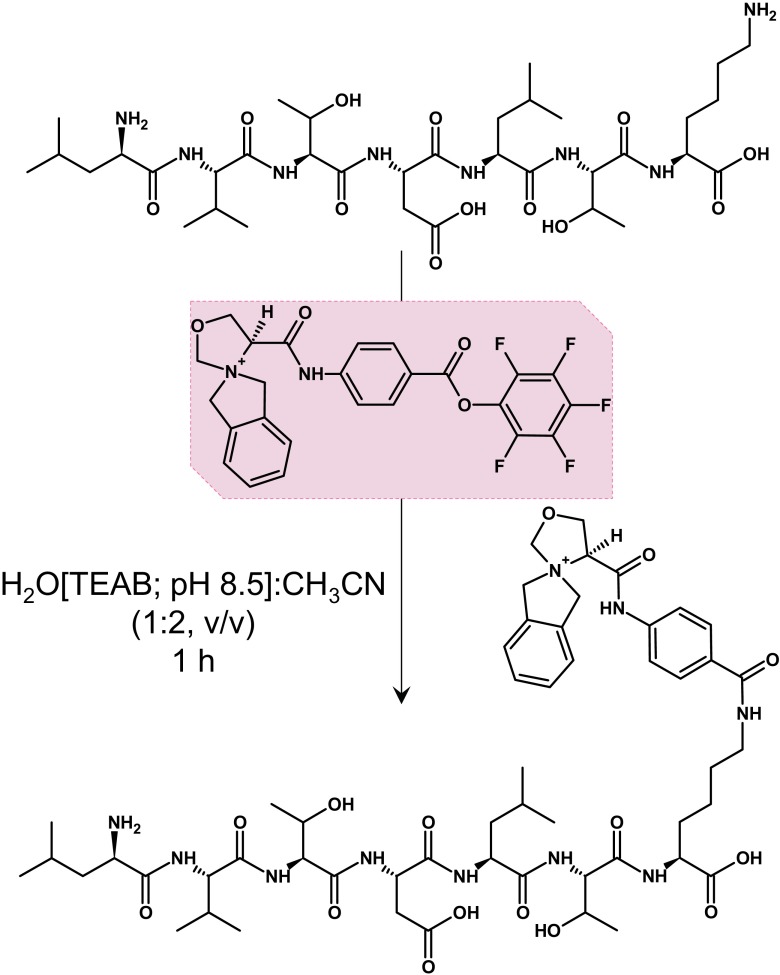
Derivatization reaction with ionization tag based on the heterocyclic analogue of the benzo-5-azoniaspiro[4.4]nonyl scaffold

A new derivatization agent, based on the oxazolidine-4-carboxylic acid and 4-aminobenzoic acid, applicable for peptide labeling in solution was designed and synthesized. The reagent is composed of (1) an ionization tag based on the heterocyclic analogue of the benzo-5-azoniaspiro[4.4]nonyl group enabling liberation of the C_9_H_10_N^+^ fragment ion during MS/MS experiment, (2) appropriate linker, and (3) the amine reactive group suitable for peptide derivatization in solution (Fig. 3.).

**Fig. 4:**
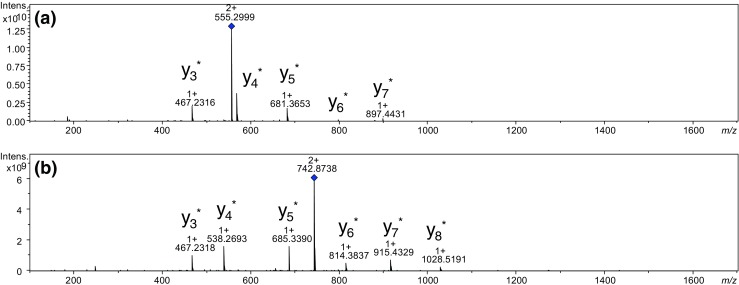
**a** ESI-MS/MS spectrum of the LVTDLTK[BASN^Oxa+^-4-Abz], *m/z* 555.299 (collision energy, 20 V); **b** ESI-MS/MS spectrum of the LVNELTEFAK[BASN^Oxa+^-4-Abz], *m/z* 742.382 (collision energy, 20 V). Asterisk is added to denote the presence of the derivative moiety that represents a charged (non-protonated) fragment. Diamond symbol denotes the parent peaks

The labeling efficiency of the synthesized ionization tag was tested on various synthetic model tryptic peptides (for peptide sequences see Table 1) from bovine serum albumin and myoglobin. Derivatization of model peptides in solution was performed using a modified method presented by Beauchamp et al. [29] and also described by us previously [20]. ESI-MS spectra recorded for the product ions of the derivatized peptides (see Figs. S24 and S25 in the ESM) unveil signals which correspond only to product ions [M]^+^ and protonated product ions [M+H]^2+^. This indicates a nearly quantitative efficiency of the labeling step for the analyzed peptides. Since the C-terminal lysine residue is derivatized, the ionization tag is located at the C-terminus of the sequence. In the ESI-MS/MS spectra of protonated parent ions [M+H]^2+^, these labeled peptides gave clear *y*_i_^*^-type ion series which facilitates unambiguous peptide sequencing (Fig. 4 as well as Figs. S26 and S27 in the ESM).

According to our previous study, derivatization of peptides with QAS at the N-terminus achieved subfemtomole to attomole levels of peptide detection by measurements in the SRM mode [18, 19]. Thus, the presented methodology makes possible the analysis of peptides with a low ionization efficiency. To establish detection limits using SRM method, we synthesized model tryptic peptide LVTDLTK and its labeled analogue LVTDLTK[BASN^Oxa+^-4-Abz]. During the initial MS/MS experiments, these peptides produced strong signals at *m/z* 185.15 (a_2_^*^) and 132.30 (C_9_H_10_N^+^), respectively. Obtained fragments were selected as product ions for SRM experiments. The observed detection limits by SRM for both compounds were estimated at low femtomole (10 fmol) and high attomole (100 amol) levels, respectively (see Figs. S30 and S32 in the ESM). The obtained results confirm that derivatization with the benzo-2-oxa-5-azoniaspiro[4.4]nonyl 4-aminobenzoic tag improves the detection limit of the model peptide.

The usefulness of 2-oxa-benzo-5-azoniaspiro[4.4]nonyl 4-aminobenzoic pentafluorophenyl ester in peptide analysis was examined using myoglobin digested with the trypsin/rLys-C mixture. Proteolysis with this mixture is highly specific and generates tryptic peptides (i.e., peptides with the C-terminal lysine or arginine residue). Furthermore, utilization of the trypsin/rLys-C enzyme mixture addresses several known limitations to trypsin digestion, i.e., protein MS analysis, digestion reproducibility, as well as protein quantification [30]. The unlabeled sample of myoglobin digest was analyzed by LC-MS technique. According to the list of products generated by ExPASy (http://web.expasy.org), nine peptide fragments were found in the trypsin/rLys-C digest, which cover 75% of the protein sequence (see Table 2).

LC-MS analysis of the charged tagged myoglobin digest is summarized in Table S2 (see the ESM as well as Fig. S50 in the ESM). The analysis showed abundance of product ions which correspond to QAS derivatives of peptides with the lysine residue(s). We identified 12 peptides with attached ionization tag(s), which were characterized by longer retention times as compared with the non-labeled peptides. The peptide derivatives with two ionization tags were present, all of them with the glycine residue at the N-terminus and the lysine residue at the C-terminus. Furthermore, peptide GHHEAELKPLAQSHATK, with three ionization tags was found. Moreover, three peptides previously not found in the peptic digest (TEAEMK, ASEDLK, and NDIAAK) were found in LC-MS chromatogram after charge derivatization. Modification of peptides with the lysine residue(s) to increase ionization efficiency is a major advantage of the proposed method, due to the fact that those peptides are usually characterized by signals of lower intensity as compared with peptides with the arginine residue(s). Although proposed derivatization strategy aim lysine side chain ε-amine group, three labeled peptides without the lysine residue were present in the LC-MS chromatogram—VEADIAGHGQEVLIR, ALELFR, and ELGFQG. However, further optimization of the labeling reaction conditions is needed for efficient N-terminal tag introduction.

**Fig. 5:**
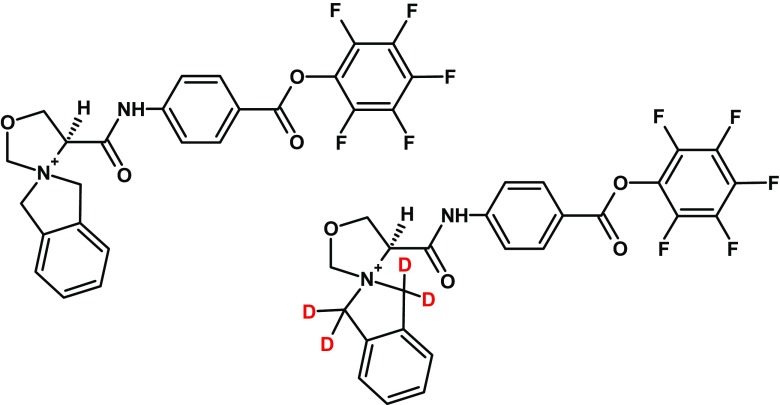
Pair of isotopologues of ionization tag based on the 2-oxa-benzo-5-azoniaspiro[4.4]nonyl scaffold. Light tag (left) and heavy tag (right)

Quantification of peptides using SRM technique requires internal standard(s) which could be added to the analyzed sample and act as reference signal(s) [31]. However, the synthesis of large quantities of peptide standards containing stable isotopes, such as ^13^C and/or ^15^N, is very expensive, time consuming, and in some cases challenging [32]. Thus, we developed a pair of deuterated isotopologues of 2-oxa-benzo-5-azoniaspiro[4.4]nonyl 4-aminobenzoic ionization tags, suitable for SRM experiments. To evaluate the appropriate placement of deuterium atoms in the QA group, we synthesized a series of 2-oxa-benzo-5-azoniaspiro[4.4]nonyl 4-aminobenzoic agents with one, two, three, and four deuterium atoms gathered around the nitrogen spiro atom (see Figs. S38, S40, S42, and S45 in the ESM). ESI-MS/MS analysis of synthesized isotopologues (see Figs. S39, S41, S43, and S45 in the ESM) indicates that only the agent with four deuterium atoms liberated a reporter ion with *m/z* value higher than *m/z* value of a non-deuterated reporter ion (*m/z* 136.113 versus 132.080). This phenomenon could be explained basing on the unique fragmentation pattern of examinated heterocyclic QAS (proposed fragmentation mechanism is present in the ESM, Scheme S5). Thus, we decided to use isotopologue with four deuterium atoms for an easy discrimination between generated light and heavy reporter ions (Fig. 5.).

**Fig. 6:**
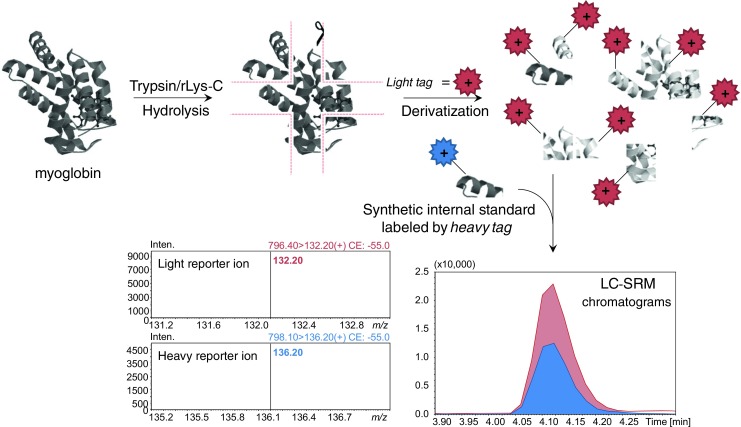
Schematic diagram of the protein quantification by a newly developed pair of light and heavy heterocyclic ionization tags. Based on the peptide sequence: LFTGHPETLEK

To verify whether the newly developed pair of light and heavy heterocyclic ionization tags could be applied in comparative targeted quantification experiments using SRM technique, we used myoglobin tryptic digests derivatized with a light tag. From 12 previously identified QAS peptides, we selected LFTGHPETLEK[BASN^Oxa+^-CO-4Abz−], due to its high abundance in the obtained LC-MS chromatogram. For the SRM experiment, *m/z* 796.40 [M+H]^2+^ ion was selected and transition of the ion at *m/z* 796.40 to *m/z* 132.20 (− 55.0 V) was monitored. Synthetic model peptide LFTGHPETLEK[BASN^Oxa+^{d_4_}-CO-4Abz−] with a stabile isotope substitution was synthesized (see Fig. S49 in the ESM) and used as the internal standard. During SRM experiment conducted for the deuterated isotopomer transition of the ion at *m/z* 798.10 to *m/z* 136.20 (− 55.0 V) was analyzed. After combining both samples, we performed LC-SRM experiment. According to obtained SRM chromatograms (Fig. 6. as well as Fig. S51 in the ESM), no influence of deuterium atoms on the elution time was observed. Obtained result indicates that the protein and the internal standard were mixed at the 2:1 ratio based on the chromatographic peaks intensity, which corresponds to the theoretical value. Furthermore, SRM experiment confirmed that selected reporter ions at *m/z* 132.20 (non-deuterated) and 136.20 (deuterated) are suitable for quantification purposes and are specifically liberated from the designed heterocyclic ionization tags during CID experiment.

## Conclusions

Peptides derivatized with the heterocyclic ionization tag based on the structure of the 5-azoniaspiro[4.4]nonyl tag are characterized by amplified ionization during ESI-MS analysis due to a permanent positive charge as well as a clear series of fragmentation ions during CID experiment. The tag undergoes selective fragmentation at CID conditions liberating a stabile reporter ion for SRM targeted quantification experiments. Obtained reagent was successfully applied in tryptic digest protein analysis by LC-MS.

Utilization of AQUA assays as well as mTRAQ quantification have significantly increased the confidence in LC-MS peptide quantification, but their high costs and a lack of ionization amplification may limit their applicability, especially for peptides of a low abundance. Our tag is a cost-effective alternative to previously proposed solutions to targeted quantification. 2-oxa-benzo-5-azoniaspiro[4.4]nonyl 4-aminobenzoic pentafluorophenyl ester is characterized by the following: (1) efficiently enhanced ionization during ESI-MS experiments, (2) greatly improved CID fragmentation pathway, (3) lack of isotopic (deuterium) effect during the LC separation on the retention times of labeled peptides, and (4) intense and stabile reporter ions during CID experiment at *m/z* 132.080 (d_0_) and 136.105 (d_4_). It may be assumed that application of our newly developed tag for targeted quantification experiments using SRM technique may find its use in peptide analysis workflow.

## Electronic supplementary material

ESM 1 (PDF 2584 kb)
