# Supplementary material for: New ionization tags based on the structure of the 5-azoniaspiro[4.4]nonyl tag for a sensitive peptide sequencing by mass spectrometry
Source: Anal Bioanal Chem. 2017 Dec 6;410(4):1311–21. doi: 10.1007/s00216-017-0771-2 (PMC5775984; doi:10.1007/s00216-017-0771-2)
Supplement: Supplementary file 1 — (PDF 2584 kb) [file 216_2017_771_MOESM1_ESM.pdf]

Analytical and Bioanalytical Chemistry

Electronic Supplementary Material

New ionization tags based on the structure of the 5-azoniaspiro[4.4]nonyl tag for a sensitive peptide sequencing by mass spectrometry

Bartosz Setner, Zbigniew Szewczuk

Table of Contents

1. ESI-MS/MS SPECTRA OF QAS DERIVATIZED MODEL TETRAPEPTIDES........ 4

2. ESI-MS SPECTRA OF THE 1:1:1 MIXTURE OF THE MODEL SYNTHETIC PEPTIDE LVTDLTK AND ITS HETEROCYCLIC QAS DERIVATIVES........ 11

3. SYNTHESIS AND ANALYSIS OF THE HETEROCYCLIC QUATERNARY AMMONIUM IONIZATION TAG [BASN^{OXA+}–CO–4ABZ–OPFP] 12

4. ESI-MS AND ESI-MS/MS SPECTRA OF THE QAS DERIVATIZED MODEL SYNTHETIC PEPTIDES EXAMINED IN THIS STUDY 17

5. SRM EXPERIMENTS – ESTIMATION OF THE DETECTION LIMIT OF THE SYNTHETIC MODEL PEPTIDE LVTDLTK AND THE QAS DERIVATIZED MODEL SYNTHETIC PEPTIDE LVTDLTK[BASN^{OXA+}–CO–4ABZ–] 19

6. SYNTHESIS AND ANALYSIS OF THE A,A'-DIBROMO-*O*-XYLENE[D_4] 22

7. SYNTHESIS AND ANALYSIS OF THE FMOC–OXA–OH[D_2] 24

8. SYNTHESIS, ESI-MS AND ESI-MS/MS ANALYSIS OF DEUTERIUM LABELED HETEROCYCLIC QUATERNARY AMMONIUM IONIZATION TAGS........ 27

9. ESI-MS/MS ANALYSIS OF THE HETEROCYCLIC QAS DERIVATIZED MODEL SYNTHETIC PEPTIDE LFTGHPETLEK[BASN^{OXA+}{D_4}–CO–4ABZ–] 33

10. LC-MS CHROMATOGRAM OF MYOGLOBIN TRYPTIC DIGEST DERIVATIZED WITH HETEROCYCLIC QAS 34

11. SRM – QUANTIFICATION EXPERIMENT USING MODEL SYNTHETIC PEPTIDE LFTGHPETLEK[BASN^{OXA+}{D_4}–CO–4ABZ–] AND MYOGLOBIN TRYPTIC DIGEST 35

12. NMR SPECTRA 35

Table S1 QAS derivatized model tetrapeptides examined in this study

Lp.	Sequence	*m/z*	Charge	*m/z* calculated	Retention time [min]
1.	ASN^{Thz+}–CO–DVYT–NH$_2$	665.270		665.296	12.6
2.	ASN^{Thz+}–CO–SWVE–NH$_2$	688.310		688.312	15.1
3.	ASN^{Thz+}–CO–DILN–NH$_2$	642.327		642.327	14.5
4.	BASN^{Thz+}–CO–DVYT–NH$_2$	713.292		713.296	14.6
5.	BASN^{Thz+}–CO–SWVE–NH$_2$	736.311		736.312	16.9
6.	BASN^{Thz+}–CO–DILN–NH$_2$	690.325		690.327	16.7
7.	ASN^{Oxa+}–CO–DVYT–NH$_2$	649.316	[M]$^+$	649.319	11.1
8.	ASN^{Oxa+}–CO–SWVE–NH$_2$	672.336		672.335	13.7
9.	ASN^{Oxa+}–CO–DILN–NH$_2$	626.351		626.350	12.3
10.	BASN^{Oxa+}–CO–DVYT–NH$_2$	697.316		697.319	12.8
11.	BASN^{Oxa+}–CO–SWVE–NH$_2$	720.335		720.335	14.6
12.	BASN^{Oxa+}–CO–DILN–NH$_2$	674.349		674.350	14.4
13.	LVTDLTK[ASN^{Thz+}–CO–]	958.516	[M]$^+$	958.527	13.9
		479.785	[M+H]$^{2+}$	479.767	
14.	LVTDLTK[BASN^{Thz+}–CO–]	1006.512	[M]$^+$	1006.527	14.6
		503.787	[M+H]$^{2+}$	503.767	
15.	LVTDLTK[ASN^{Oxa+}–CO–]	942.540	[M]$^+$	942.550	13.1
		471.803	[M+H]$^{2+}$	471.778	
16.	LVTDLTK[BASN^{Oxa+}–CO–]	990.516	[M]$^+$	990.550	14.1
		495.798	[M+H]$^{2+}$	495.778	
17.	LVTDLTK[BASN^{Thz+}–CO–4Abz–]	1125.562	[M]$^+$	1125.564	17.5
		563.297	[M+H]$^{2+}$	563.286	
18.	LVTDLTK[BASN^{Oxa+}–CO–4Abz–]	1109.585	[M]$^+$	1109.587	17.1
		555.293	[M+H]$^{2+}$	555.297	

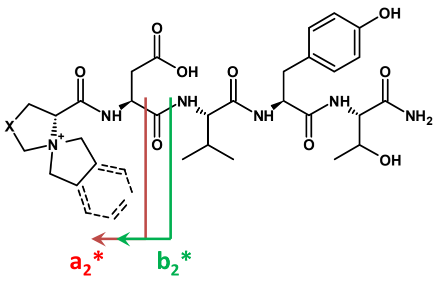

Scheme S1 Proposed nomenclature for a* and b*-type ions. X = sulfur or oxygen atom

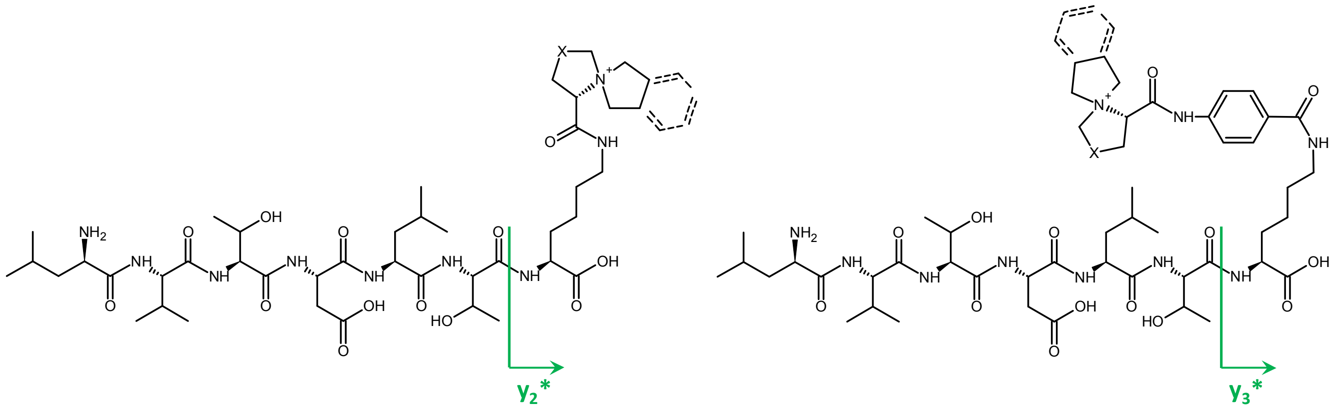

Scheme S2 Proposed nomenclature for y*-type ions. X = sulfur or oxygen atom

1. ESI-MS/MS spectra of QAS derivatized model tetrapeptides

a. ASN^{Thz+}–CO–SWVE–NH$_2$

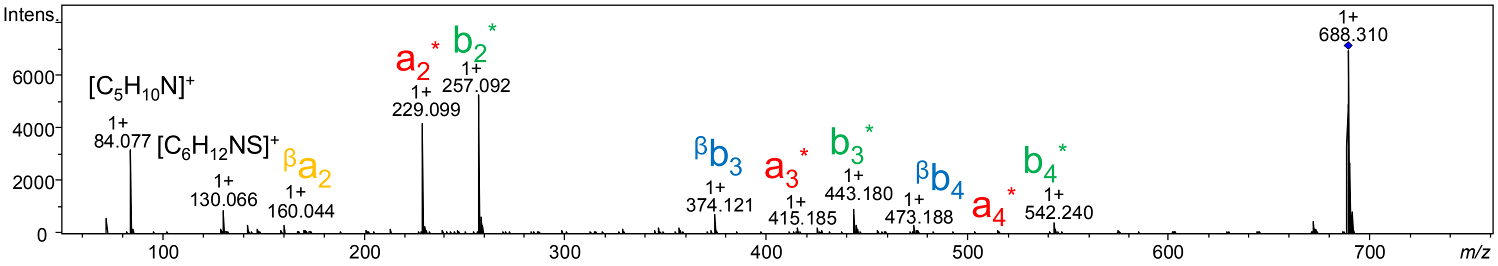

Fig. S1 ESI-MS/MS spectrum of the ion at *m/z* 688.310 [ASN^{Thz+}–CO–SWVE–NH$_2$]. Collision energy 25 V

b. BASN^{Thz+}–CO–SWVE–NH$_2$

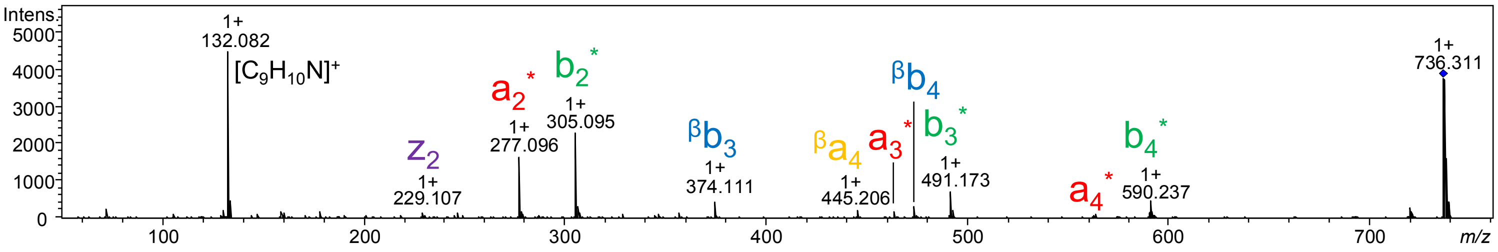

Fig. S2 ESI-MS/MS spectrum of the ion at *m/z* 736.311 [BASN^{Thz+}–CO–SWVE–NH$_2$]. Collision energy 25 V

c. ASN^{Thz+}–CO–DILN–NH$_2$

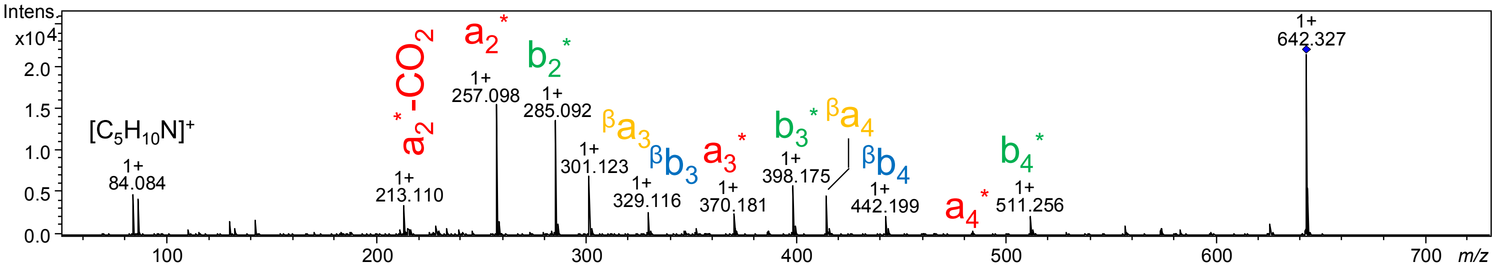

Fig. S3 ESI-MS/MS spectrum of the ion at *m/z* 642.327 [ASN^{Thz+}–CO–DILN–NH$_2$]. Collision energy 25 V

d. BASN^{Thz+}–CO–DILN–NH$_2$

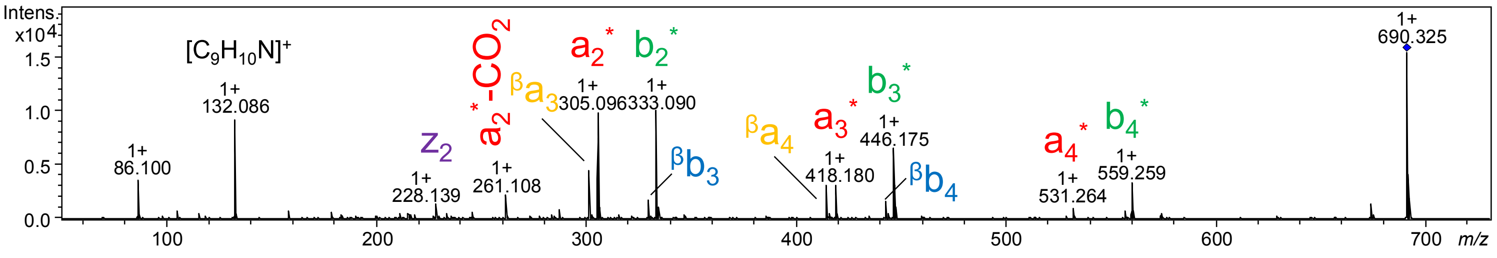

Fig. S4 ESI-MS/MS spectrum of the ion at *m/z* 690.325 [BASN^{Thz+}–CO–DILN–NH$_2$]. Collision energy 25 V

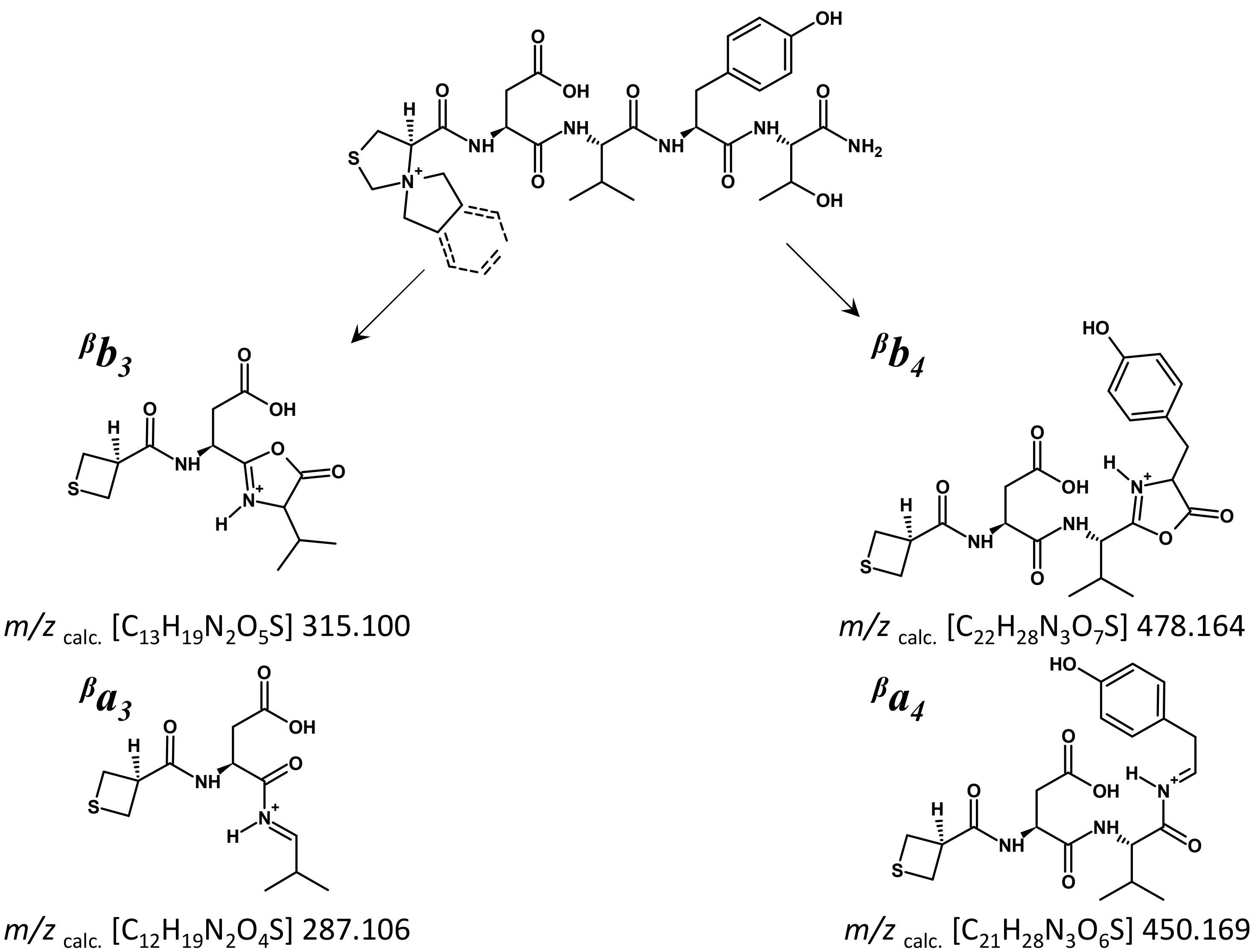

Scheme S3 Proposed $^{\beta}a_i$ and $^{\beta}b_i$ ion fragments derived from the liberation of the C_4H_7N fragment from the ASN^{Thz+}-CO-DVYT-NH_2 or C_8H_7N fragment from the $BASN^{Thz+}$-CO-DVYT-NH_2

Author's comment

Due to the present of identical *m/z* values marked as $^{\beta}a_i$ and $^{\beta}b_i$ on the MS/MS spectra of the 2-thia-5-azoniaspiro[4.4]nonyl derivative as well as the 2-thia-benzo-5-azoniaspiro[4.4]nonyl derivatives author's assumed fragmentation of quaternary ammonium salts, during which 2-thia-5-azoniaspiro[4.4]nonyl group liberated C_4H_7N fragment and 2-thia-benzo-5-azoniaspiro[4.4]nonyl group liberated C_8H_7N fragment. Calculated *m/z* values are present in Scheme S3

e. *ASN*$^{Oxa+}$*–CO–SWVE–NH*$_2$

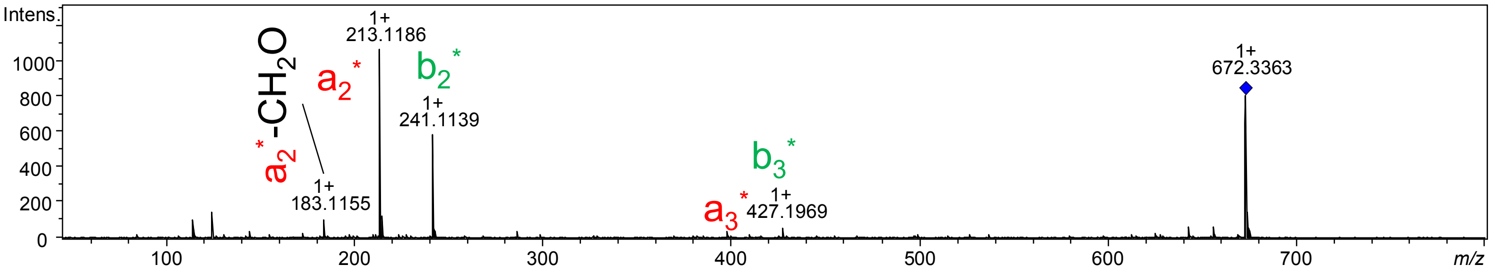

Fig. S5 ESI-MS/MS spectrum of the ion at *m/z* 672.336 [ASN^{Oxa+}–CO–SWVE–NH$_2$]. Collision energy 25 V

f. *BASN*$^{Oxa+}$*–CO–SWVE–NH*$_2$

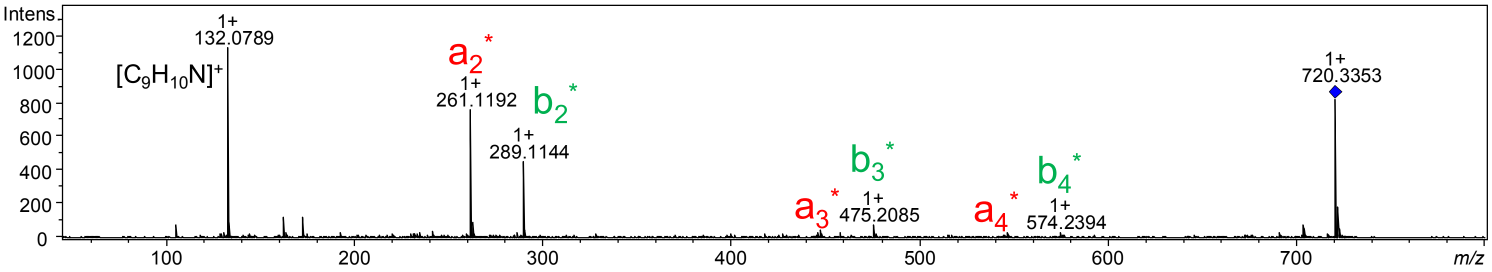

Fig. S6 ESI-MS/MS spectrum of the ion at *m/z* 720.335 [BASN^{Oxa+}–CO–SWVE–NH$_2$]. Collision energy 25 V

g. *ASN*$^{Oxa+}$*–CO–DILN–NH*$_2$

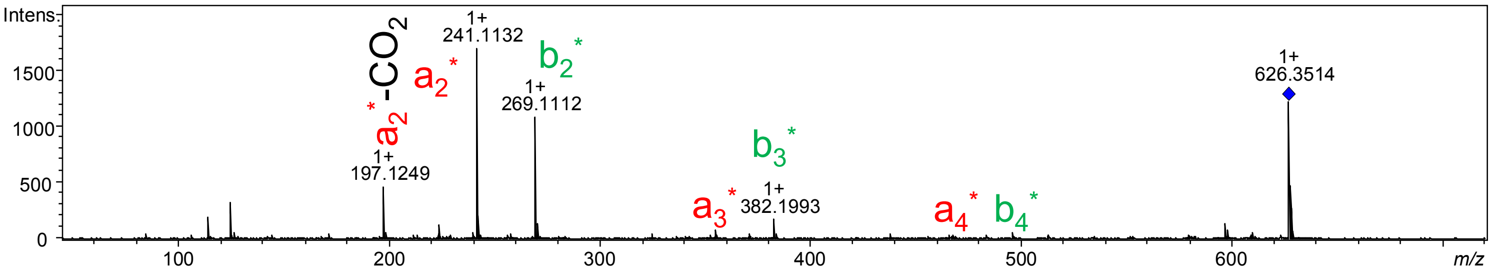

Fig. S7 ESI-MS/MS spectrum of the ion at *m/z* 626.351 [ASN^{Oxa+}–CO–DILN–NH$_2$]. Collision energy 25 V

h. *BASN*$^{Oxa+}$*–CO–DILN–NH*$_2$

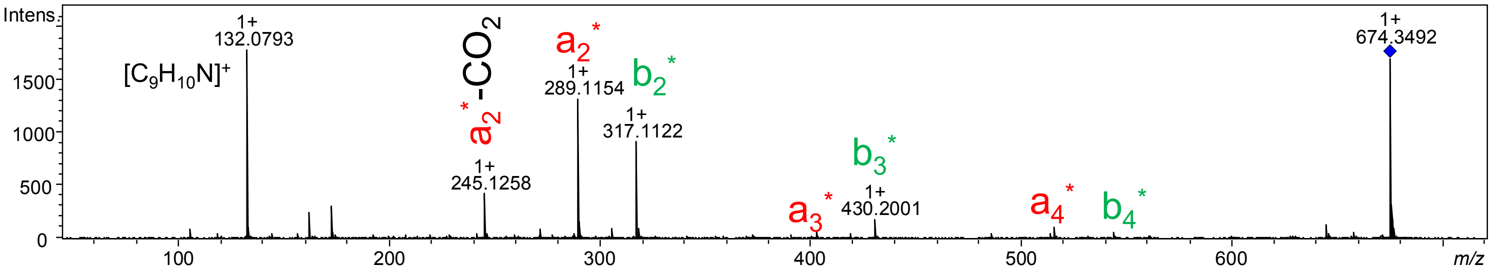

Fig. S8 ESI-MS/MS spectrum of the ion at *m/z* 674.349 [BASN^{Oxa+}–CO–DILN–NH$_2$]. Collision energy 25 V

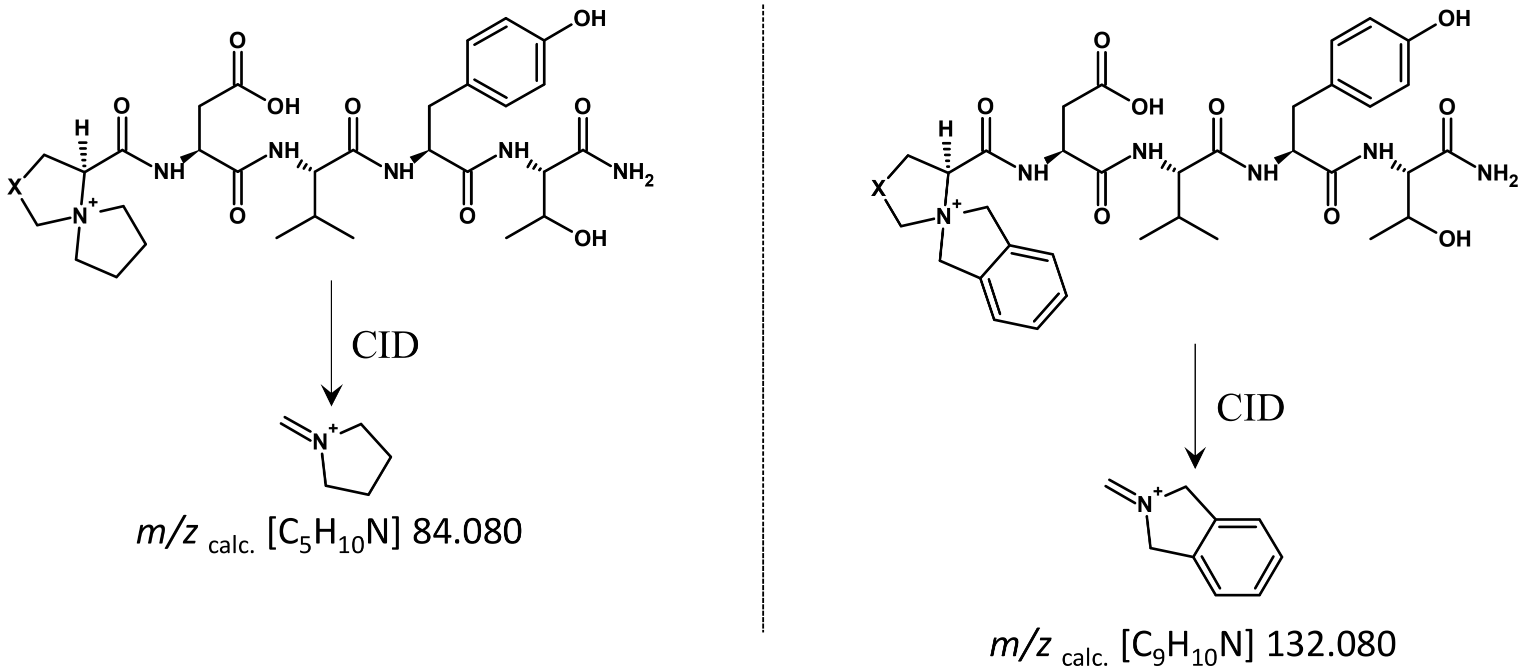

Scheme S4 Proposed structures of the reporter ion fragments derived from the liberation of the $C_5H_{10}N^+$ fragment from the ASN^{Thz+}-CO-DVYT-NH_2 or $C_9H_{10}N^+$ fragment from the $BASN^{Thz+}$-CO-DVYT-NH_2. X = sulfur or oxygen atom

i. LVTDLTK[ASN^{Thz+}–CO–]

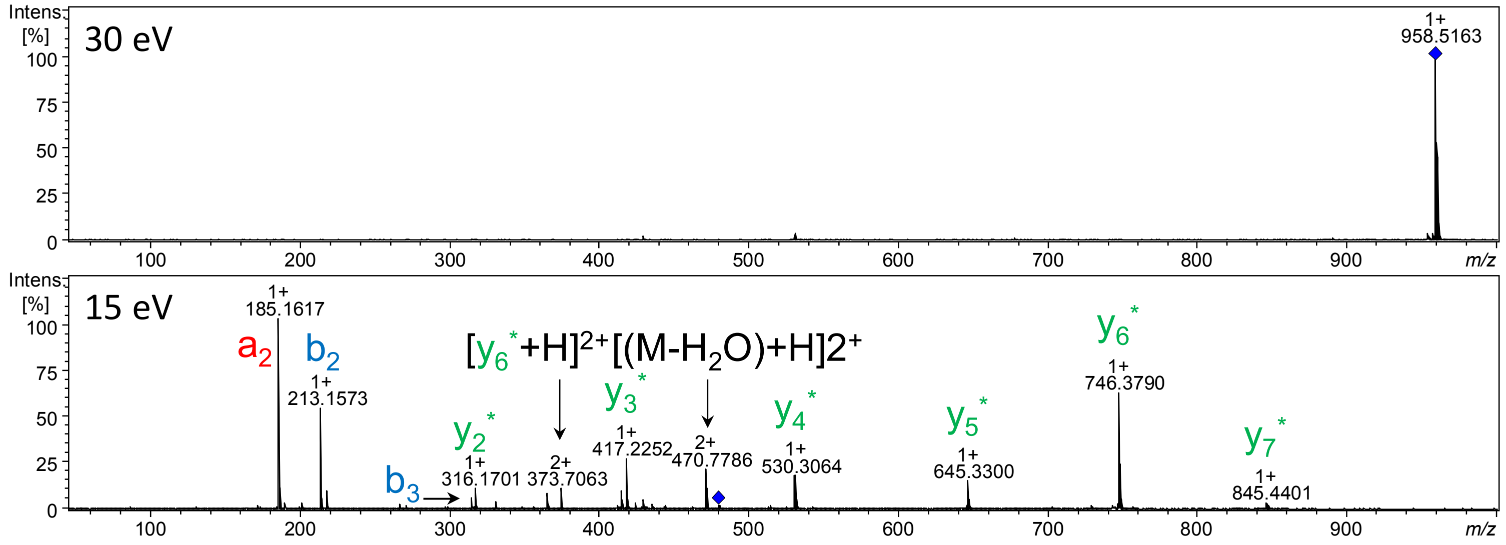

Fig. S9 (a) ESI-MS/MS spectrum of the ion at *m/z* 958.516. Collision energy 30V. (b) ESI-MS/MS spectrum of the ion at *m/z* 479.785. Collision energy 15 V

j. LVTDLTK[BASN^{Thz+}–CO–]

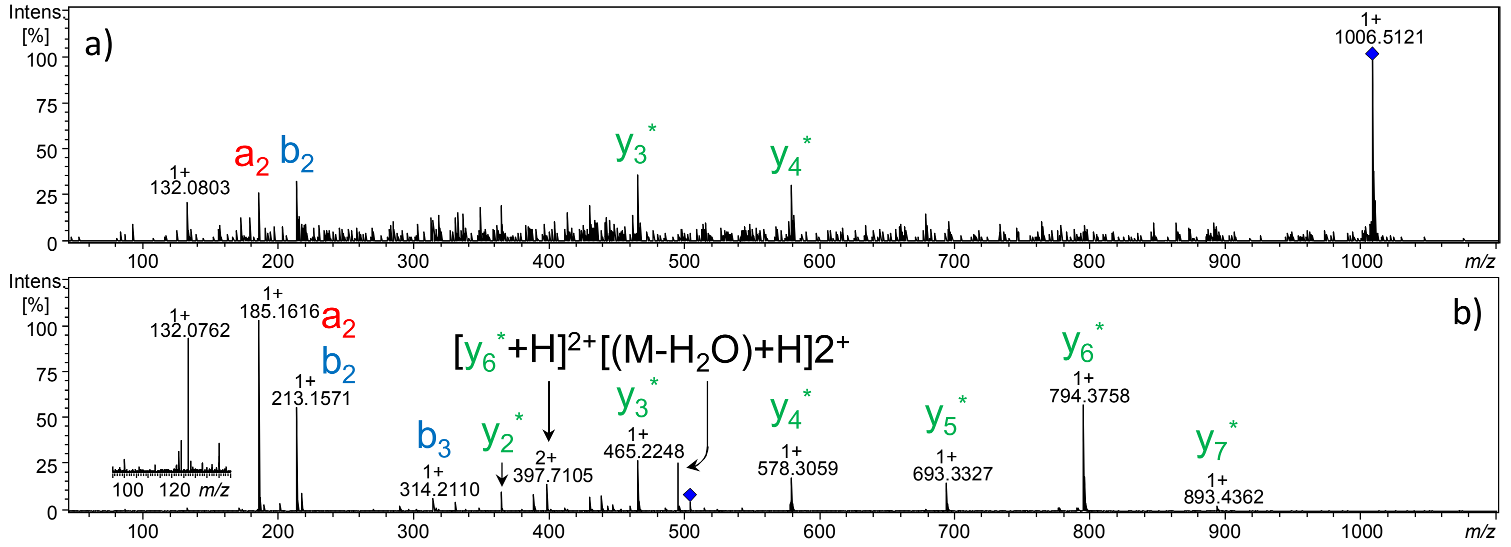

Fig. S10 (a) ESI-MS/MS spectrum of the ion at *m/z* 1006.512. Collision energy 40V. (b) ESI-MS/MS spectrum of the ion at *m/z* 503.787. Collision energy 15 V

k. LVTDLTK[ASN^{Oxa+}–CO–]

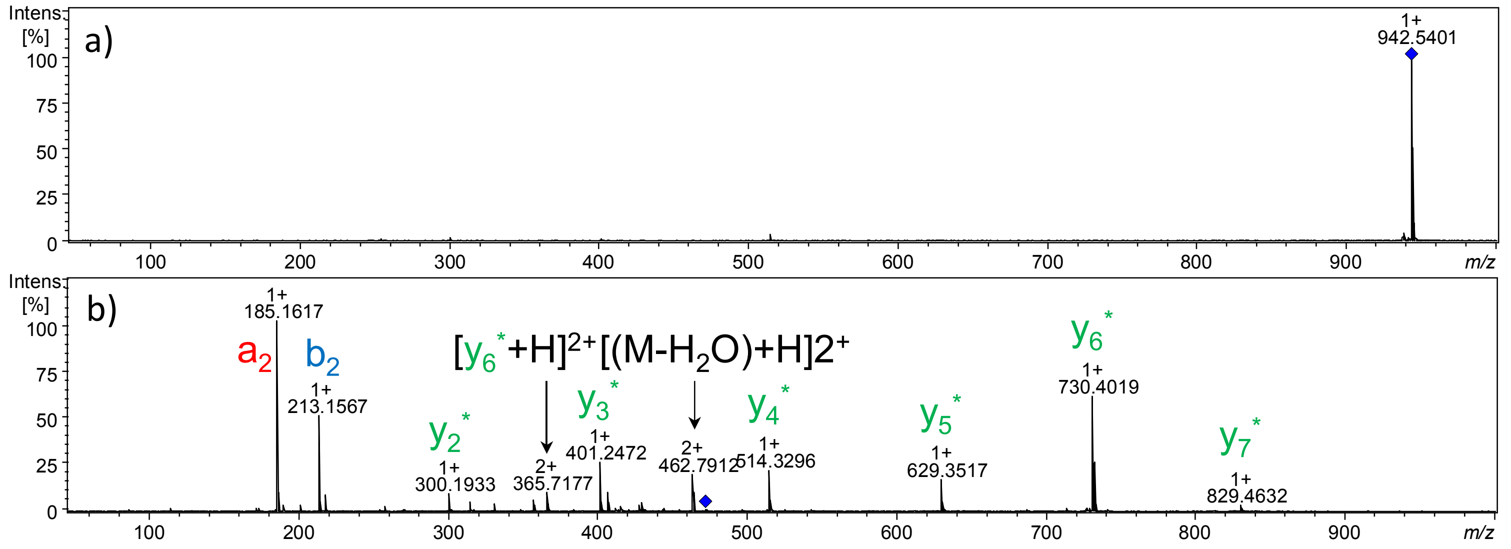

Fig. S11 (a) ESI-MS/MS spectrum of the ion at *m/z* 942.540. Collision energy 30V. (b) ESI-MS/MS spectrum of the ion at *m/z* 471.803. Collision energy 15 V

l. LVTDLTK[BASN^{Oxa+}–CO–]

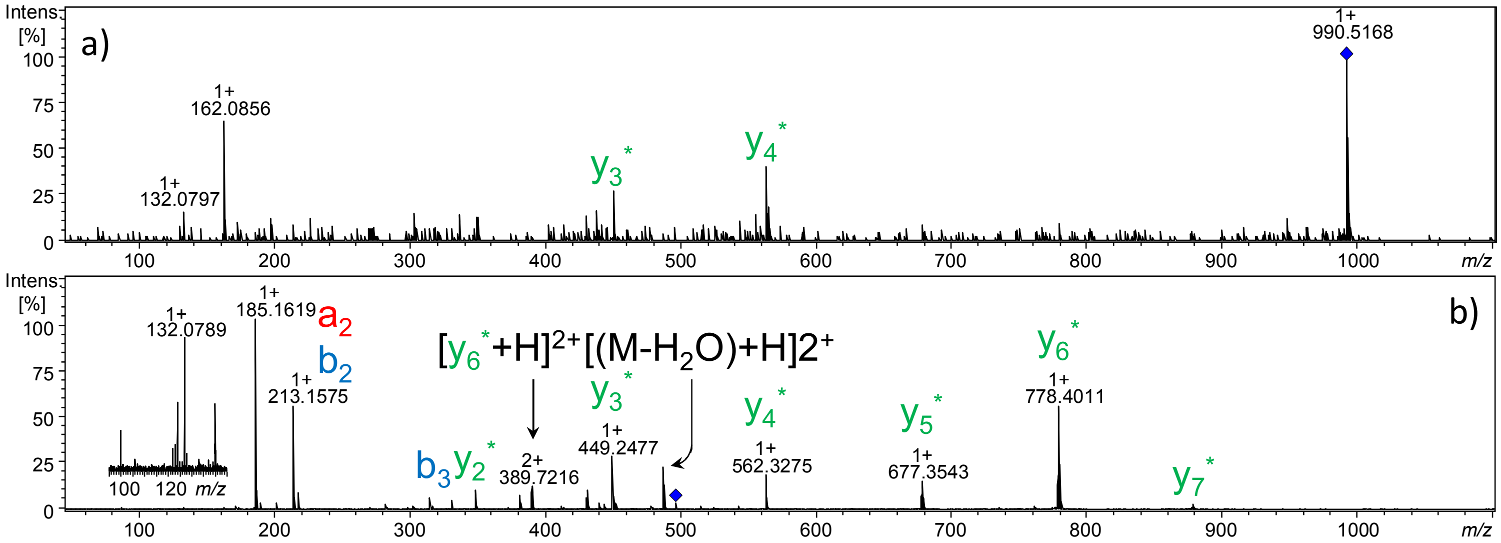

Fig. S12 (a) ESI-MS/MS spectrum of the ion at *m/z* 990.516. Collision energy 40V. (b) ESI-MS/MS spectrum of the ion at *m/z* 495.798. Collision energy 15 V

m. LVTDLTK[BASN^{Thz+}–CO–4Abz–]

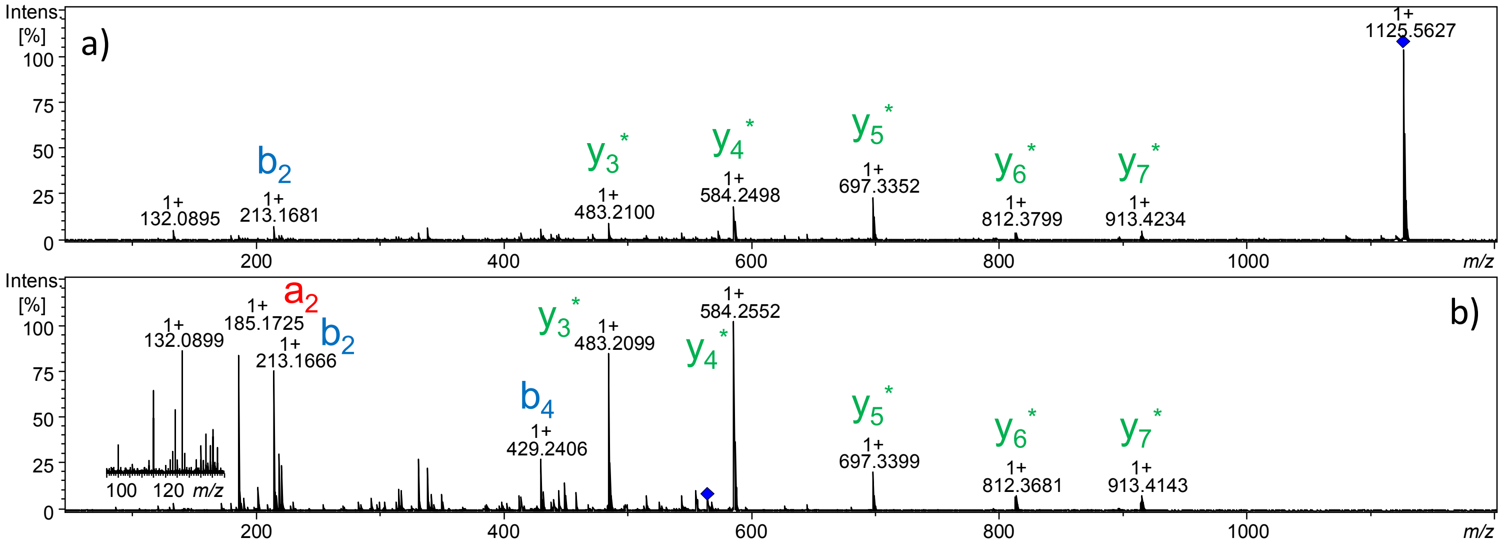

Fig. S13 (a) ESI-MS/MS spectrum of the ion at *m/z* 1125.562. Collision energy 40V. (b) ESI-MS/MS spectrum of the ion at *m/z* 563.297. Collision energy 20 V

n. LVTDLTK[BASN^{Oxa+}–CO–4Abz–]

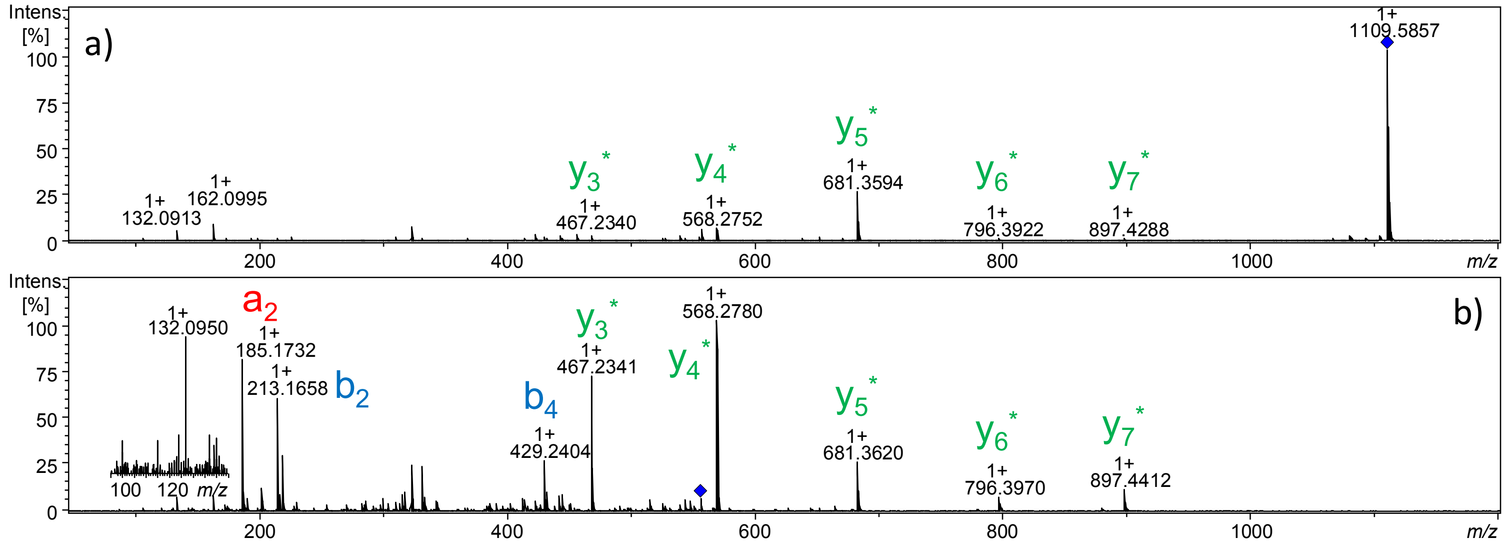

Fig. S14 (a) ESI-MS/MS spectrum of the ion at *m/z* 1109.585. Collision energy 40V. (b) ESI-MS/MS spectrum of the ion at *m/z* 555.293. Collision energy 20 V

2. **ESI-MS spectra of the 1:1:1 mixture of the model synthetic peptide LVTDLTK and its heterocyclic QAS derivatives**

   a. *LVTDLTK: LVTDLTK[ASN^{Thz+}–CO–]: LVTDLTK[BASN^{Thz+}–CO–] (1:1:1 mixture)*

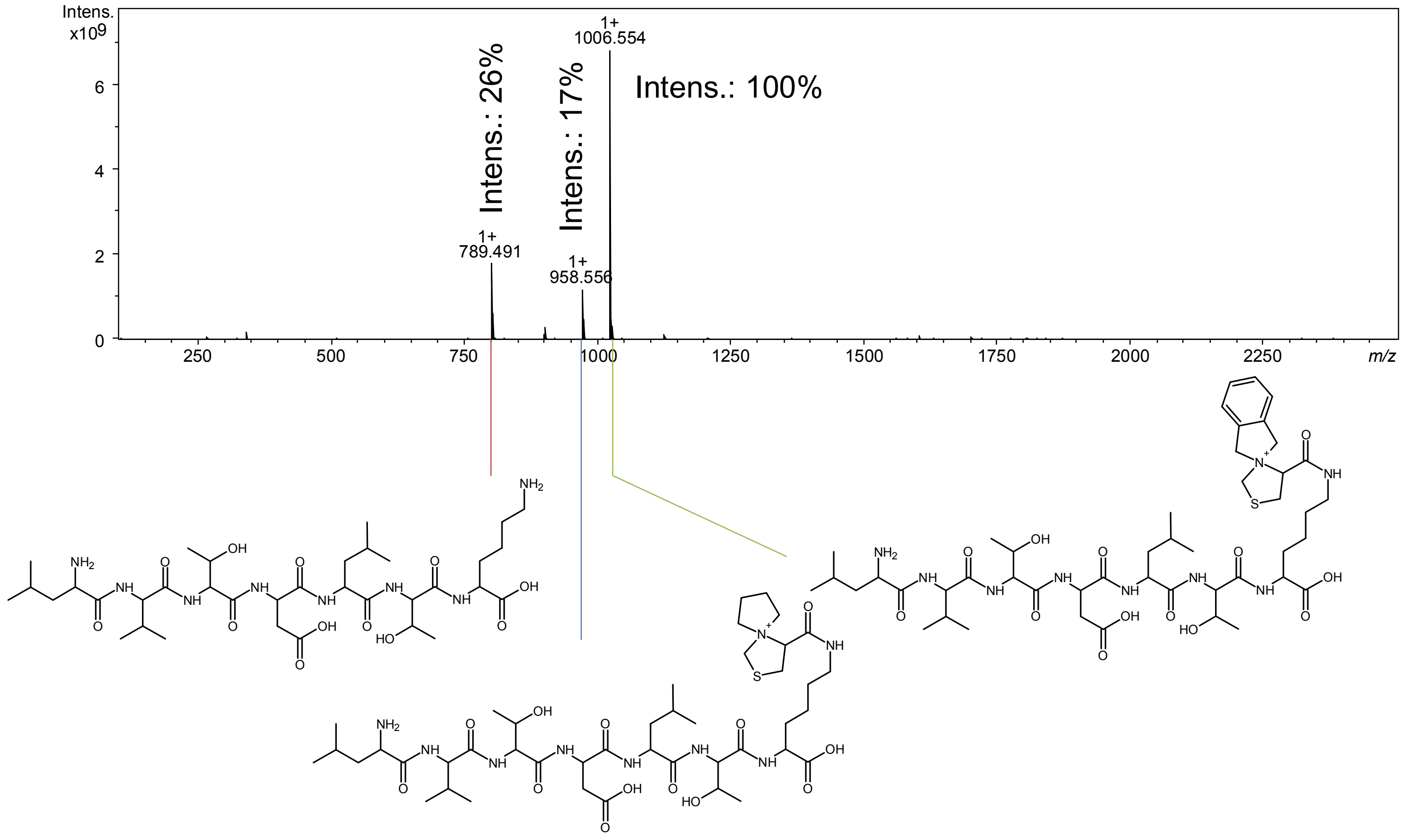

Fig. S15 ESI-MS spectrum of the 1:1:1 mixture of the model synthetic peptide LVTDLTK, LVTDLTK[ASN^{Thz+}–CO–] and LVTDLTK[BASN^{Thz+}–CO–]. Solvent: 10 mM NH_4HCOO[pH 3.2]:CH_3CN (1:1)

b. LVTDLTK: LVTDLTK[ASN^{Oxa+}–CO–]: LVTDLTK[BASN^{Oxa+}–CO–] (1:1:1 mixture)

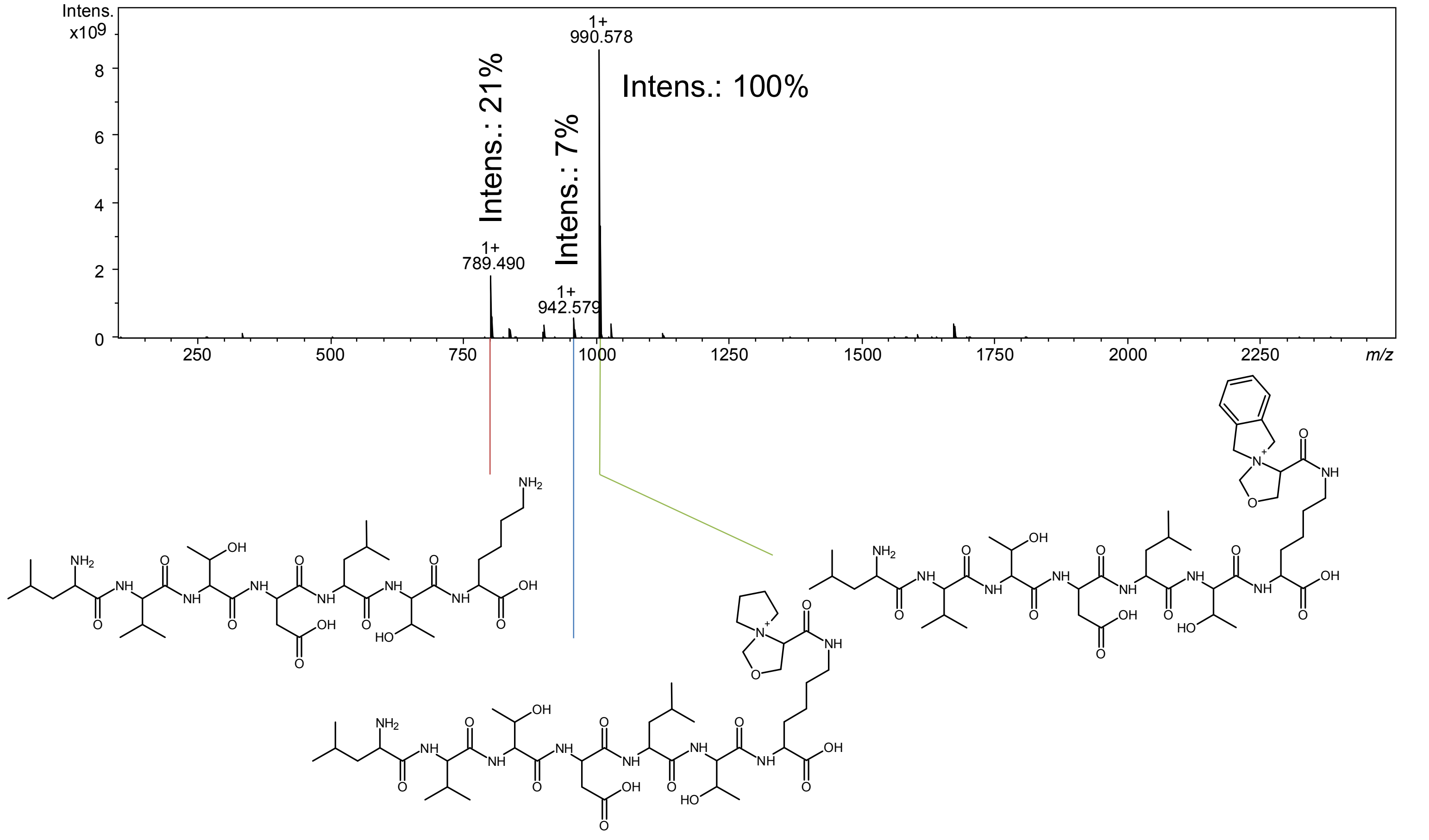

Fig. S16 ESI-MS spectrum of the 1:1:1 mixture of the model synthetic peptide LVTDLTK, LVTDLTK[ASN^{Oxa+}–CO–] and LVTDLTK[BASN^{Oxa+}–CO–]. Solvent: 10 mM NH_4HCOO[pH 3.2]:CH_3CN (1:1)

3. Synthesis and analysis of the heterocyclic quaternary ammonium ionization tag [BASN^{Oxa+}–CO–4Abz–OPfp]

a. Synthesis

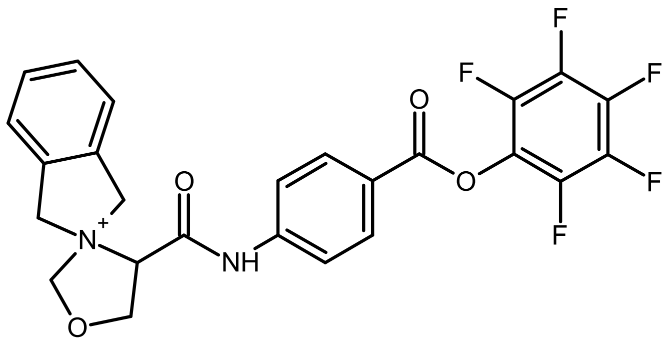

Preloaded 2-chlorotrityl resin (4–aminobenzoic acid–2CT resin) was used for the syntheses of the heterocyclic quaternary ammonium ionization tag (**Fehler! Verweisquelle konnte nicht gefunden werden.**). Briefly: the heterocyclic quaternary ammonium salt group was synthesized in three steps: (1) the Fmoc-oxazolidine-4-carboxylic acid was coupled to the free *N*–terminal amine group using HATU/Oxyma Pure/DIPEA mixture; (2) the Fmoc- group was removed from the *N*-terminal amino group with 25% solution of piperidine in DMF; (3) five-fold excess of α,α'–dibromo–*o*–xylene in DMF was added (reaction time: 24 h). The compound was cleaved from the resin using a solution of TFA/H_2O/TIS (95:2.5:2.5, v/v/v) at room temperature for 2 h.

Active ester was prepared in solution using pentafluorophenyl trifluoroacetate reagent according to procedure previously described in the literature.[1]

ESI-MS analysis:

BASN^{Oxa+}–CO–4Abz–OH *m/z* 339.135 [calc. for $C_{19}H_{19}N_2O_4$ 339.133]

BASN^{Oxa+}–CO–4Abz–OPfp *m/z* 505.119 [calc. for $C_{25}H_{18}F_5N_2O_4$ 505.118]

NMR analysis:

^{1}H NMR (500 MHz, CD_3CN, 300 K): δ: 4.55 (dd, *J* = 5.7 Hz, 1H); 4.76 (dd, *J* = 7.9 Hz, 1H); 4.99 (t, *J* = 5.0 Hz, 1H); 5.02-5.03 (m, 1H); 5.09 (dd, *J* = 14.5 Hz, 2H); 5.25 (d, *J* = 14.2 Hz, 1H); 5.50 (dd, *J* = 5.7 Hz, 1H); 7.36-7.42 (m, 4H); 7.77-7.79 (m, 2H); 7.97-8.00 (m, 2H); 11.80 (s, 1H).

^{13}C{H} NMR (125 MHz, CD_3CN, 300 K): δ: 62.4; 68.8; 70.2; 71.4; 93.7; 120.8; 124.3; 124.5; 127.6; 130.2; 130.2; 131.7; 133.3; 134.2; 143.0.

b. *ESI-MS spectra*

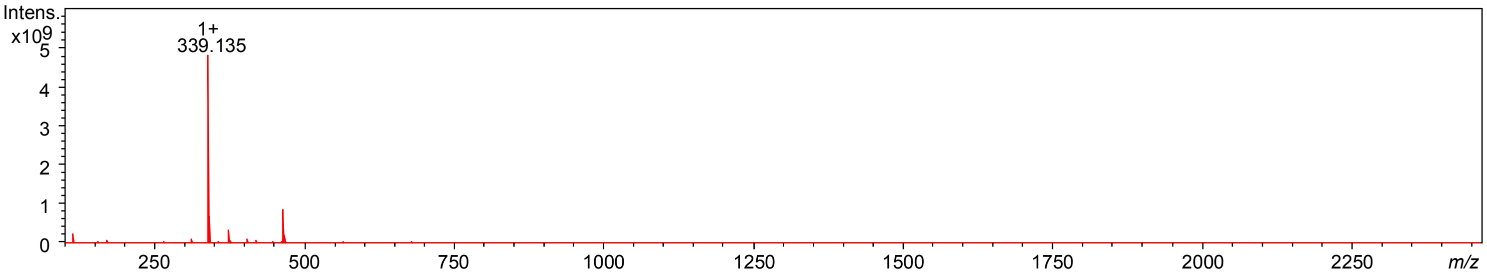

Fig. S17 ESI-MS spectrum of the heterocyclic quaternary ammonium ionization tag [BASN^{Oxa+}–CO–4Abz–OH]

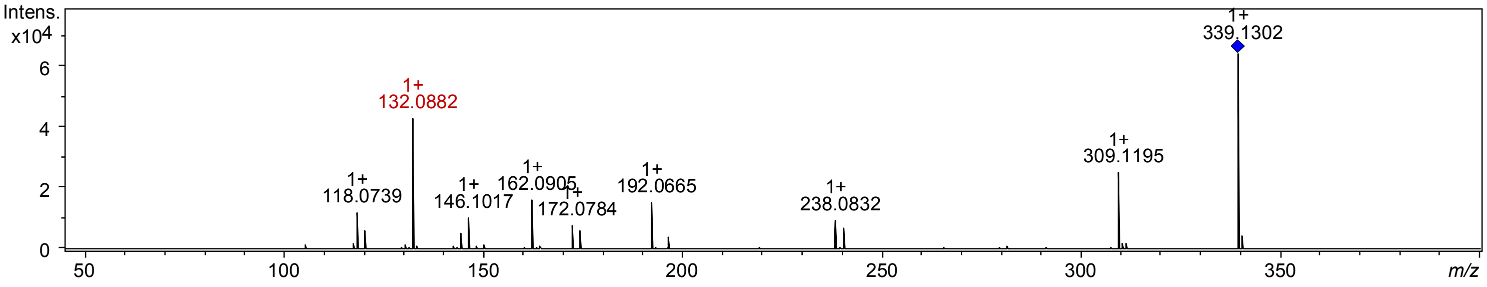

Fig. S18 ESI-MS/MS spectrum of the ion at *m/z* 339.130. Collision energy 20 V

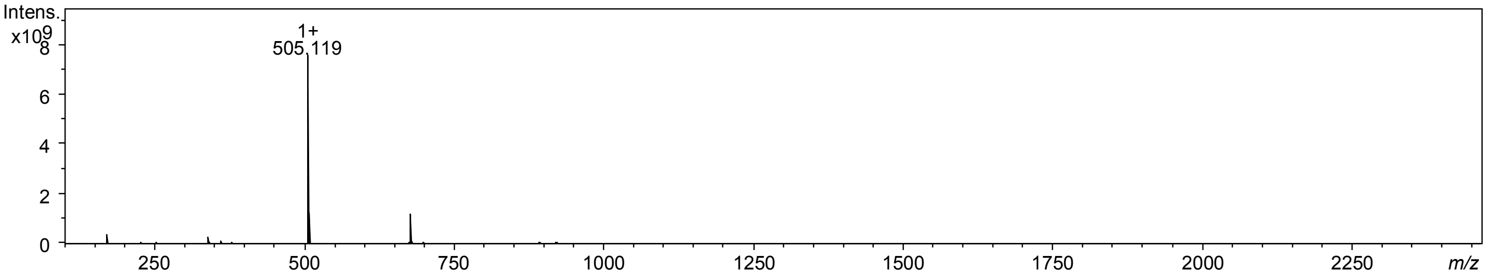

Fig. S19 ESI-MS spectrum of the heterocyclic quaternary ammonium ionization tag [BASN^{Oxa+}–CO–4Abz–OPfp]

c. NMR spectra

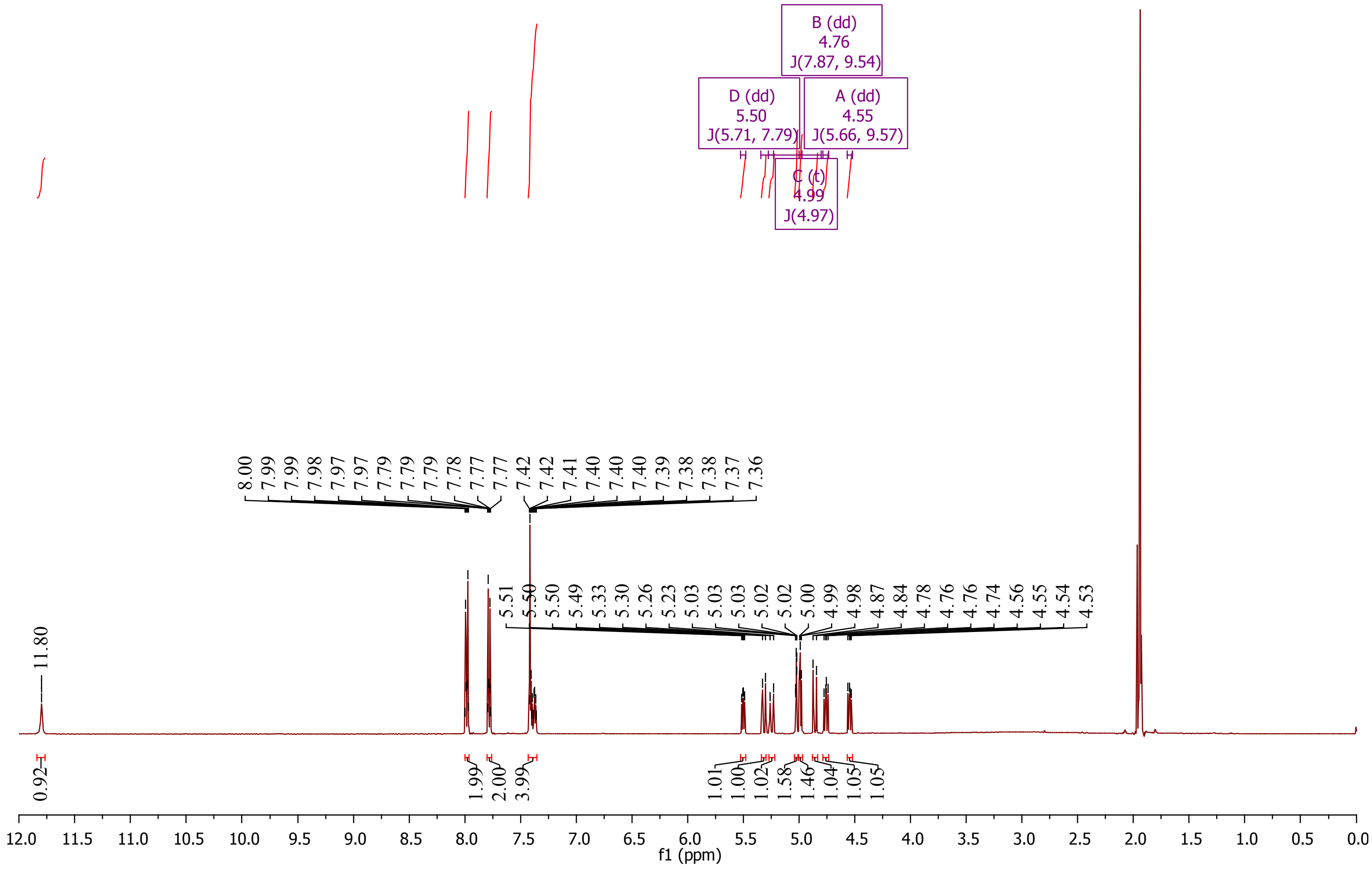

Fig. S20 ^{1}H NMR (500 MHz, CD_3CN, 300 K, σ) spectrum of the heterocyclic quaternary ammonium ionization tag [BASN^{Oxa+}–CO–4Abz–OH]

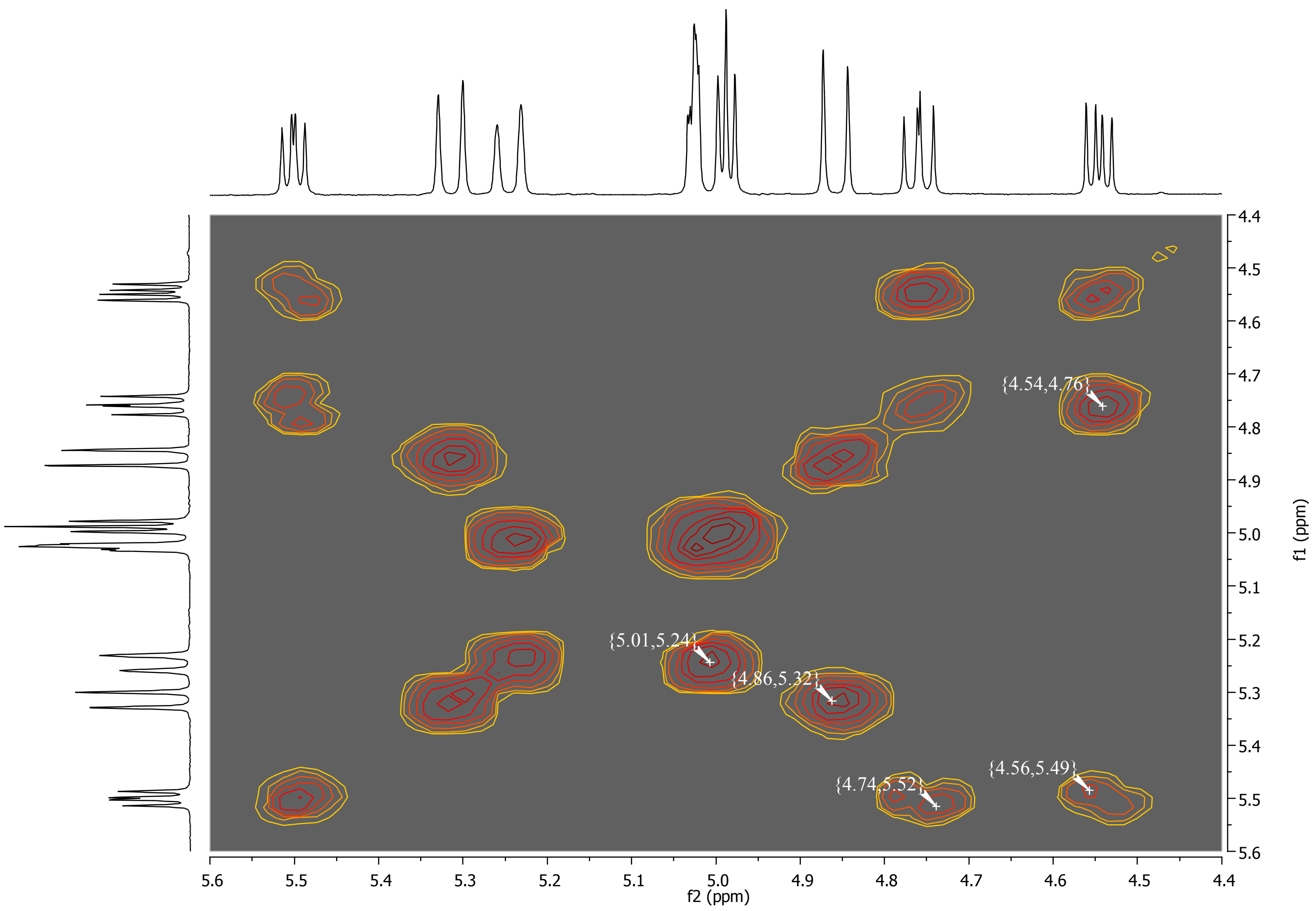

Fig. S21 COSY spectrum of the heterocyclic quaternary ammonium ionization tag [BASN^{Oxa+}–CO–4Abz–OH]

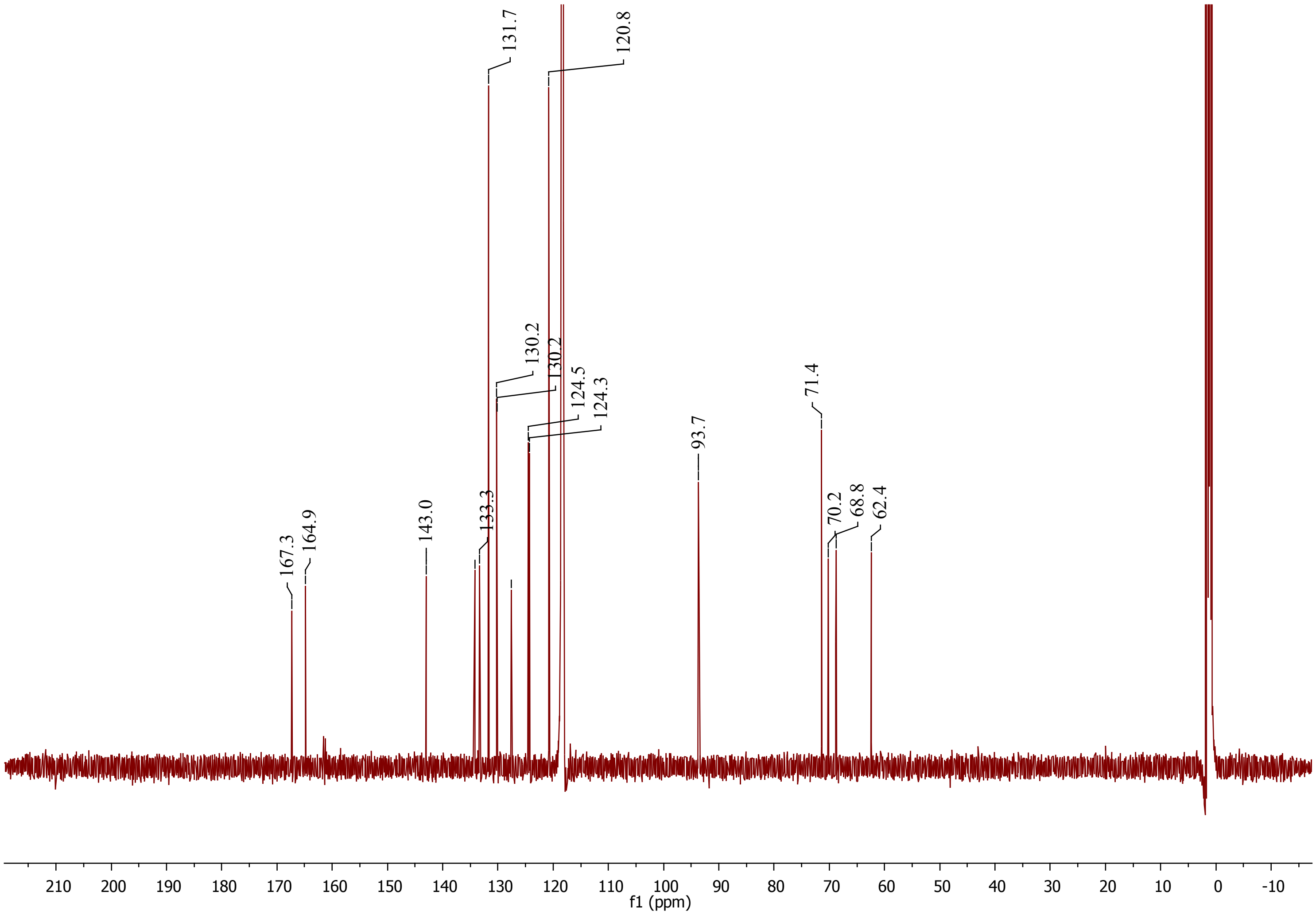

Fig. S22 ^{13}C NMR (125 MHz, CD_3CN, 300 K, σ) spectrum of the heterocyclic quaternary ammonium ionization tag [BASN^{Oxa+}–CO–4Abz–OH]

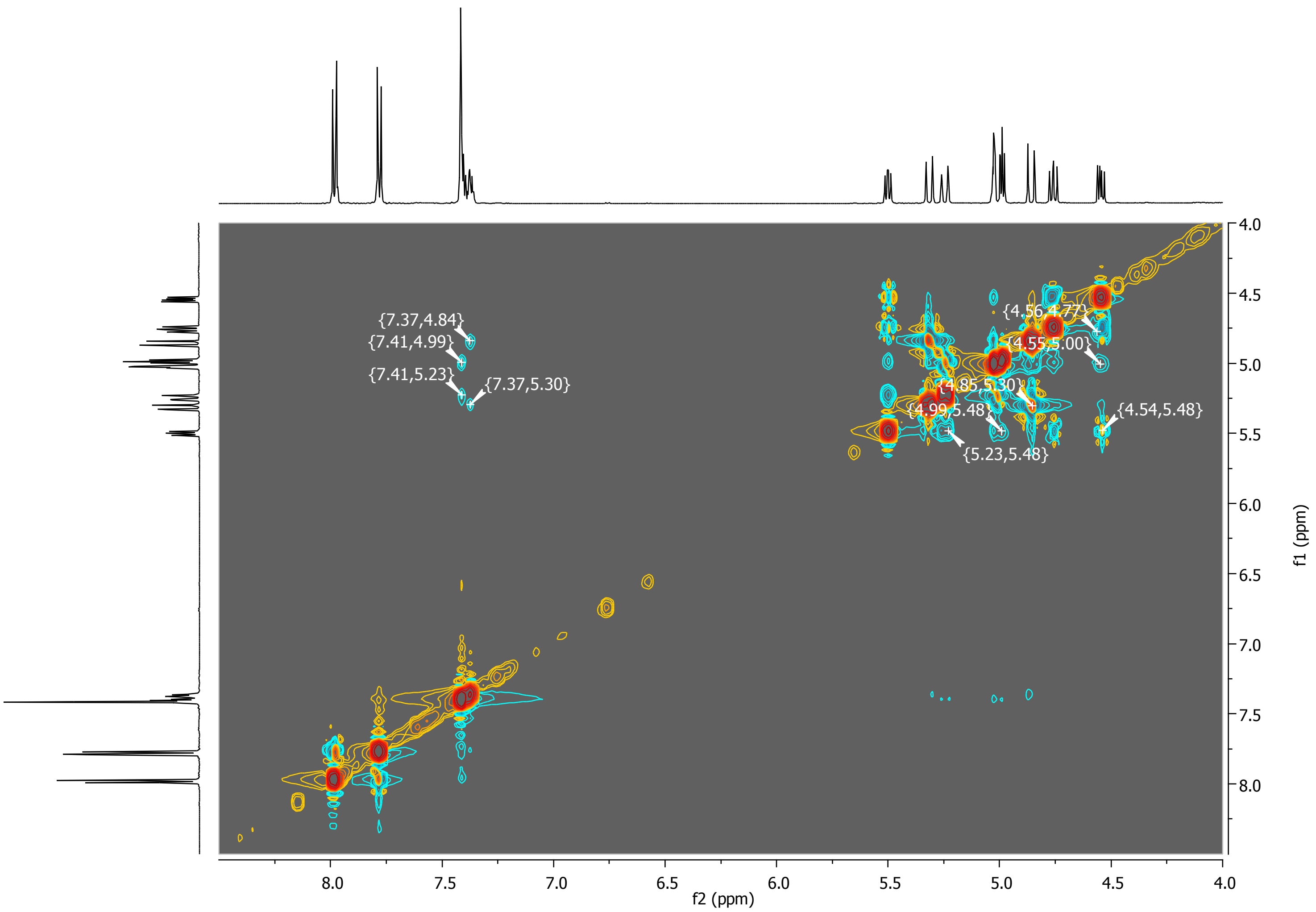

Fig. S23 NOESY spectrum of the heterocyclic quaternary ammonium ionization tag [BASN^{Oxa+}–CO–4Abz–OH]

4. ESI-MS and ESI-MS/MS spectra of the QAS derivatized model synthetic peptides examined in this study

a. LVTDLTK[BASN^{Oxa+}–CO–4Abz–]

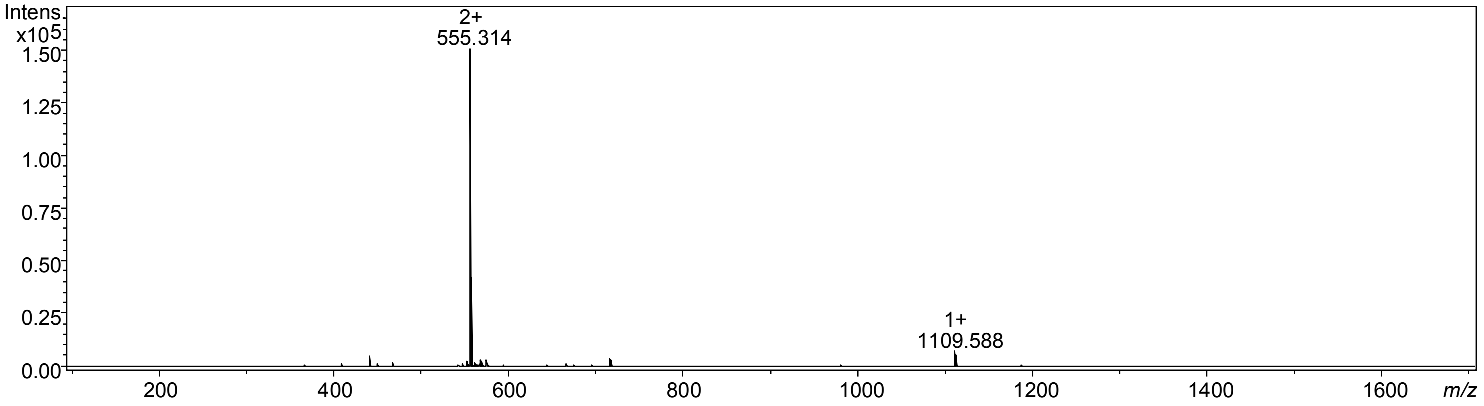

Fig. S24 ESI-MS spectrum of the model synthetic peptide LVTDLTK labeled by the heterocyclic quaternary ammonium ionization tag [BASN^{Oxa+}–CO–4Abz–OPfp]

b. LVNELTEFAK[BASN^{Oxa+}–CO–4Abz–]

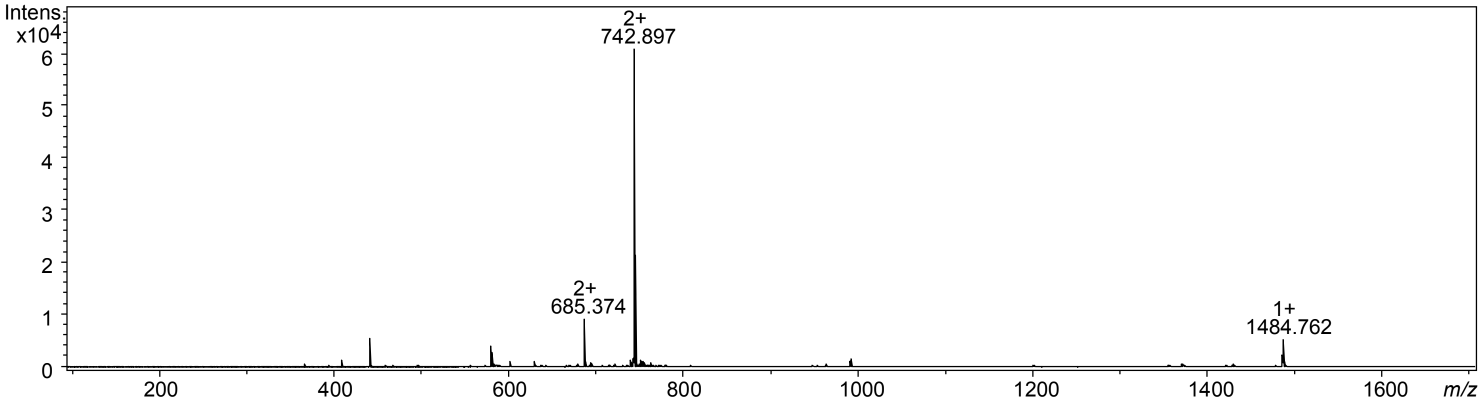

Fig. S25 ESI-MS spectrum of the model synthetic peptide LVNELTEFAK labeled by the heterocyclic quaternary ammonium ionization tag [BASN^{Oxa+}–CO–4Abz–OPfp]

c. HGTWLTALGGILK[BASN^{Oxa+}–CO–4Abz–]

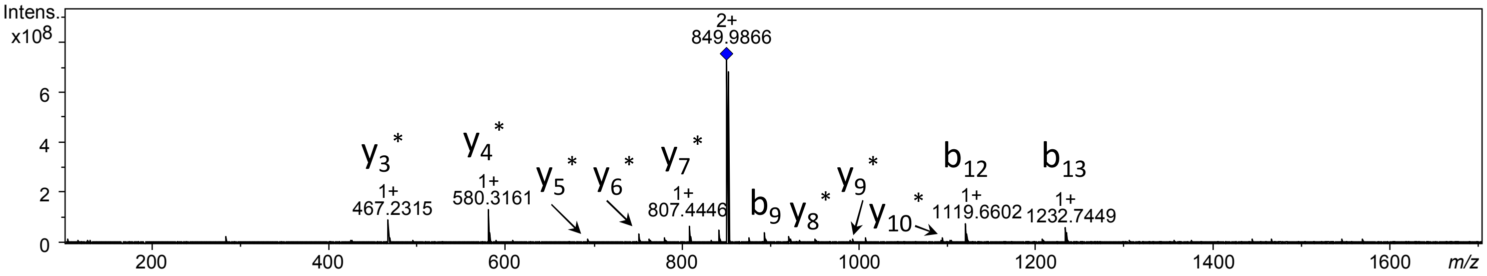

Fig. S26 ESI-MS/MS spectrum of the ion at *m/z* 849.986. Collision energy 20 V

d. LFTGHPETLEK[BASN^{Oxa+}–CO–4Abz–]

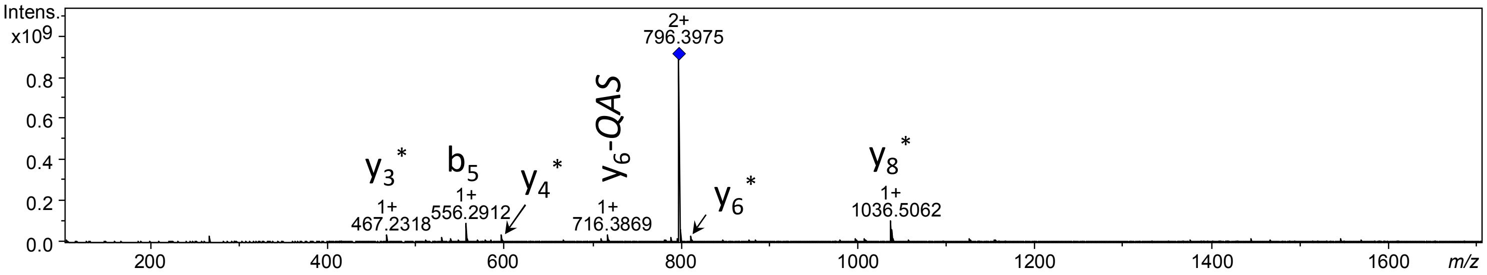

Fig. S27 ESI-MS/MS spectrum of the ion at *m/z* 796.397. Collision energy 20 V

5. SRM experiments – estimation of the detection limit of the synthetic model peptide LVTDLTK and the QAS derivatized model synthetic peptide LVTDLTK[BASN^{Oxa+}–CO–4Abz–]

a. Synthetic model peptide LVTDLTK

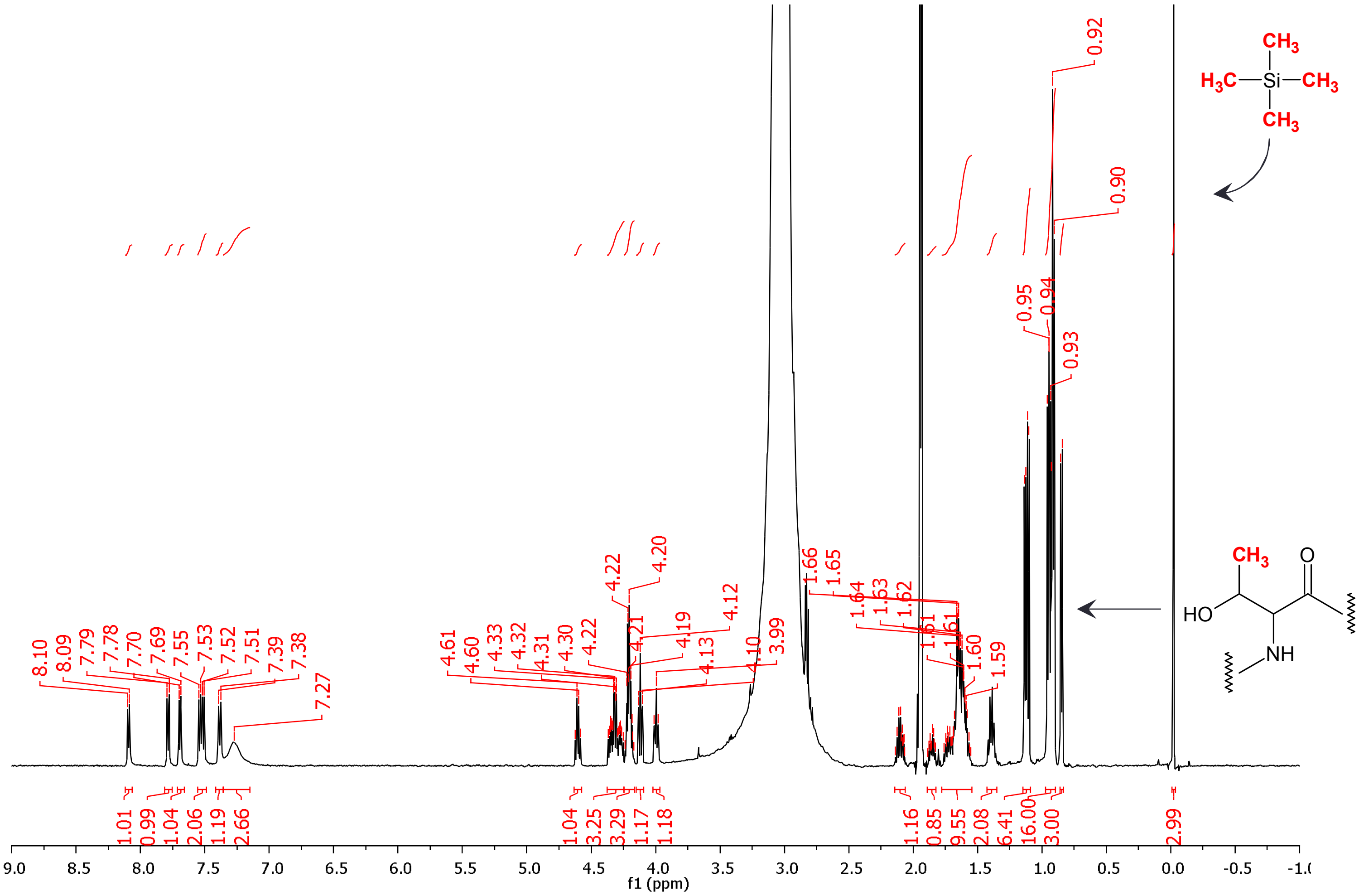

Fig. S28 ^{1}H NMR (500 MHz, CD_3CN/H_2O[9:1], 300 K, σ) spectrum of the 1:4 mixture of tetrametylsilane (TMS) and synthetic model peptide LVTDLTK

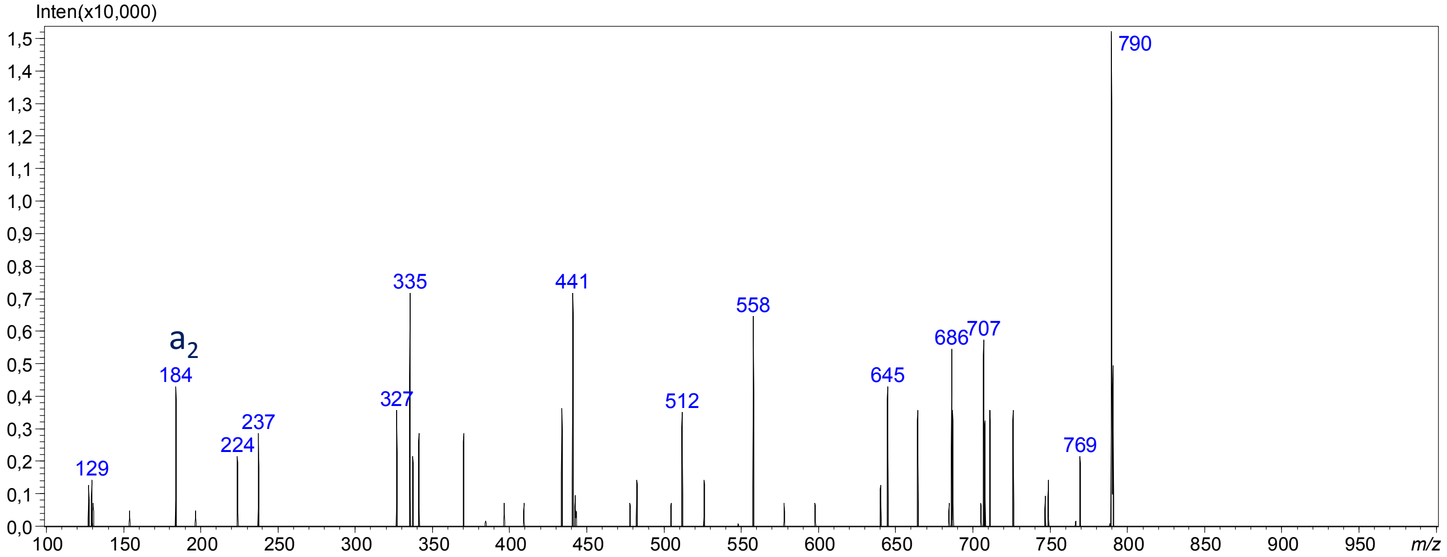

Fig. S29 ESI-MS/MS spectrum of the ion at *m/z* 790. Collision energy 25 V

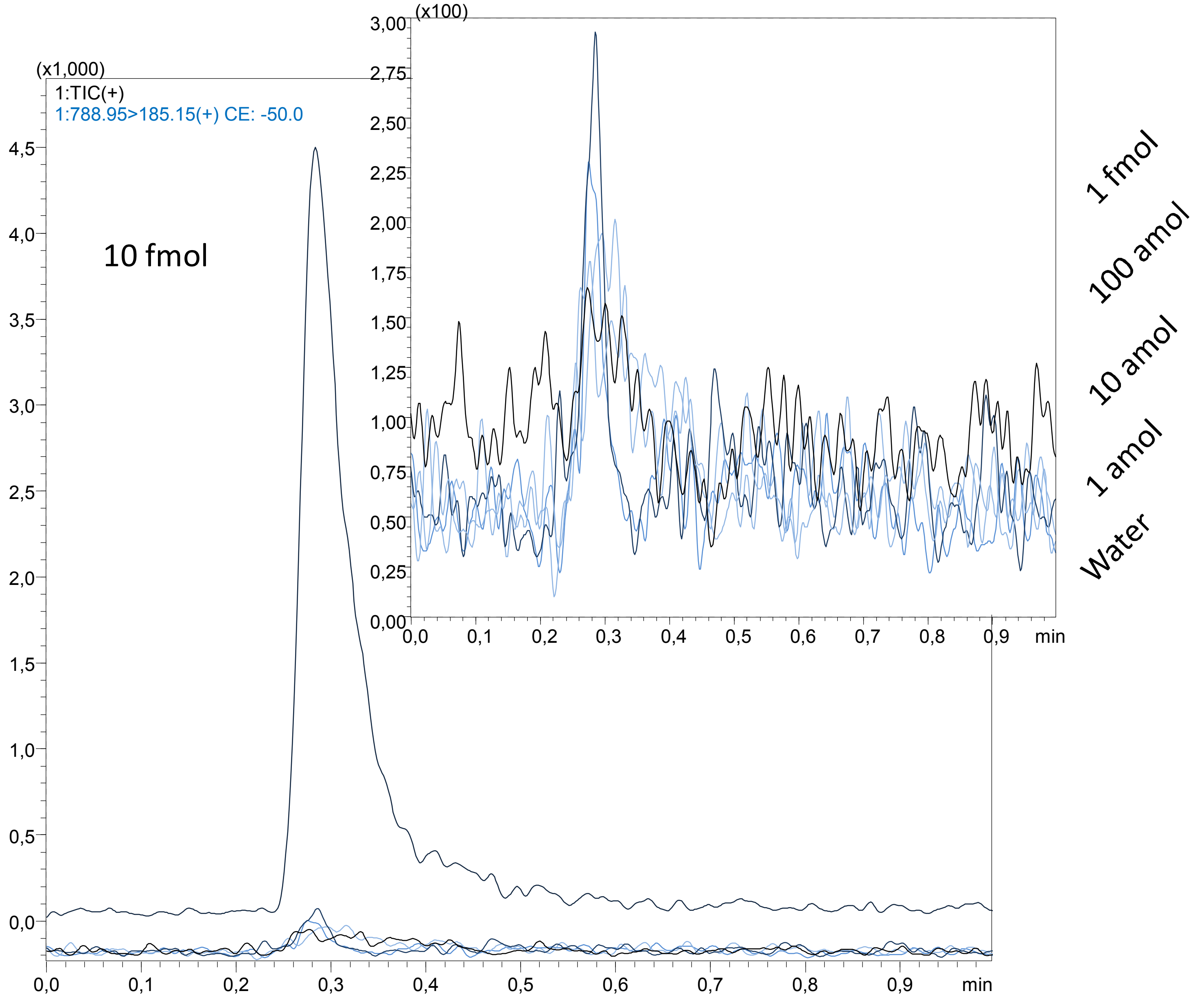

Fig. S30 SRM chromatograms of the synthetic model peptide LVTDLTK. SRM transition: *m/z* 788.95 to *m/z* 185.15(+), CE: -50.0 V

b. QAS derivatized model peptide LVTDLTK[BASN^{Oxa+}–CO–4Abz–]

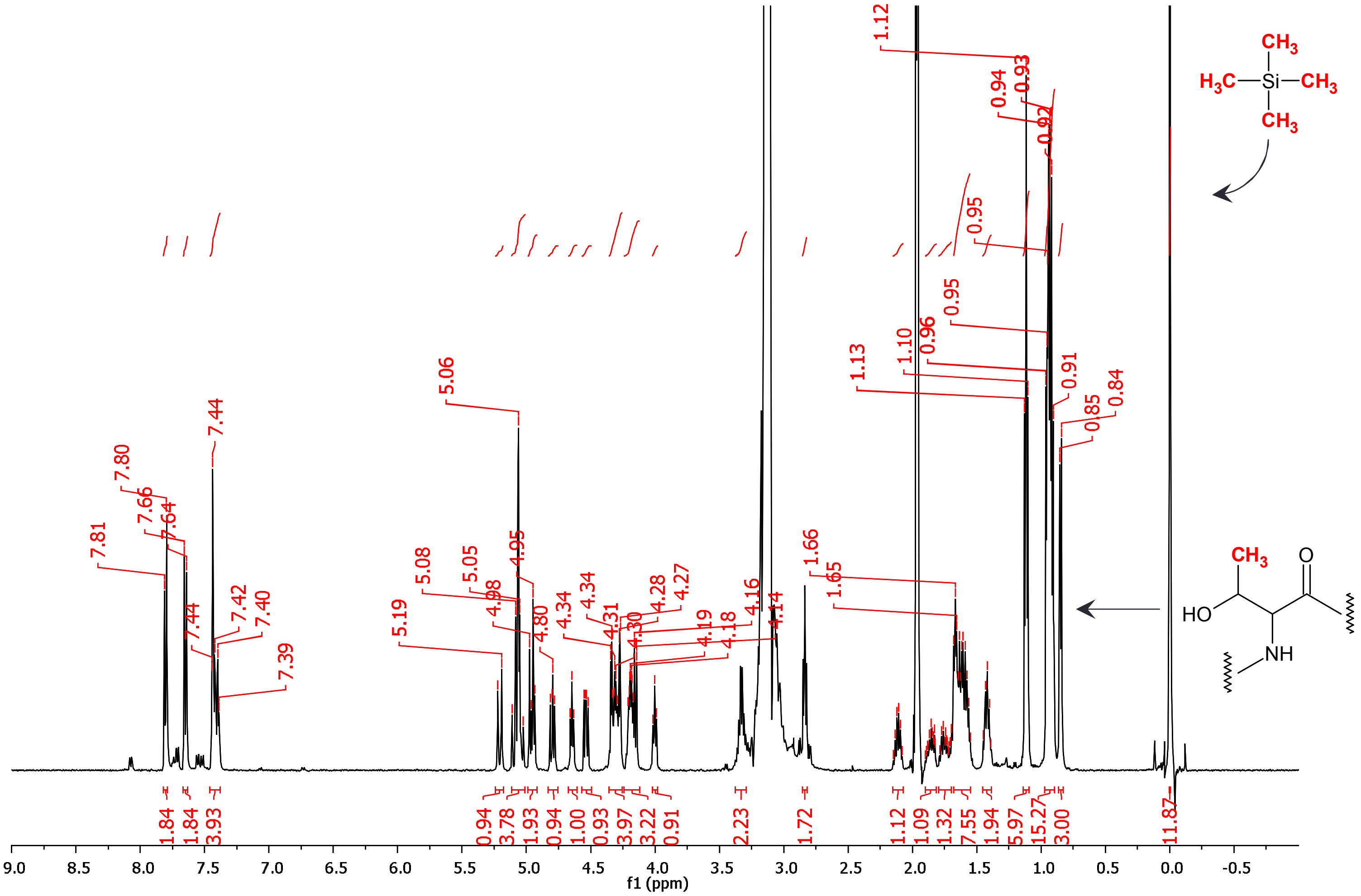

Fig. S31 ^{1}H NMR (500 MHz, CD_3CN, 300 K, σ) spectrum of the 1:1 mixture of tetrametylsilane (TMS) and the QAS derivatized model peptide LVTDLTK[BASN^{Oxa+}–CO–4Abz–]

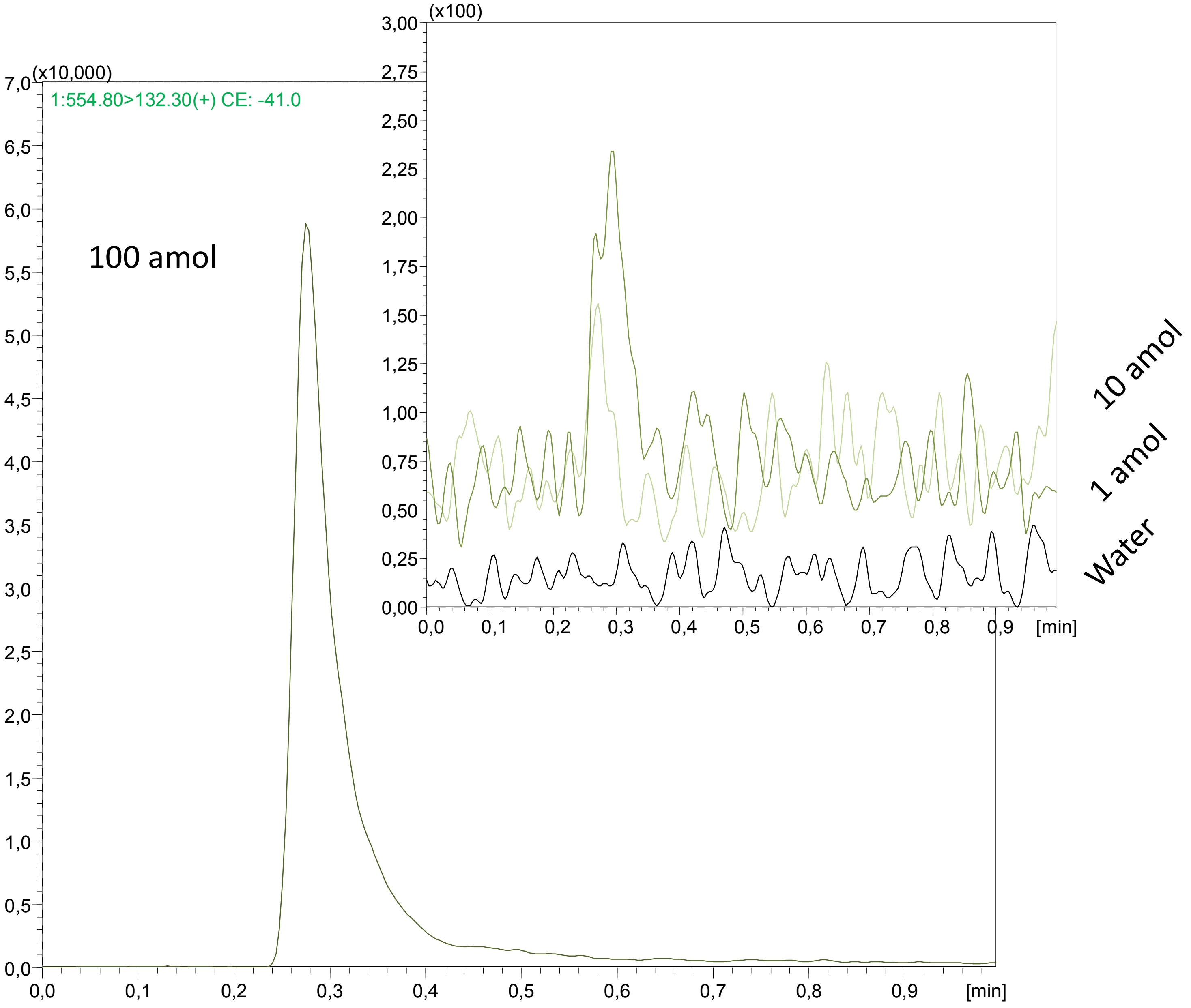

Fig. S32 SRM chromatograms of the QAS derivatized model peptide LVTDLTK[BASN^{Oxa+}–CO–4Abz–]. SRM transition: *m/z* 554.80 to *m/z* 132.30(+), CE: -41.0 V

6. Synthesis and analysis of the α,α'-dibromo-*o*-xylene[d_4]

a. Synthesis

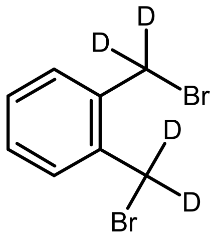

The deuterated isotopologue of the α,α'-dibromo–*o*–xylene was synthesized according to the modified procedure described by Shaw and co-workers.[2] To 50 mL roundbottom flask equipped with a magnetic stirrer 500 μL (4.2 mmole) of the *o*–xylene–(dimethyl–d_6) in 10 mL of trifluorotoluene was added. Next 215 μL (4.2 mmol) of Br_2 was added dropwise over 5 minutes. The mixture was then irradiated with a 300 W halogen bulb about 15 cm away from the flask for 30 min (until the intense orange color faded). After cooling the solution to room temperature, 25 mL of trifluorotoluene was added. Extraction of the organic phase was done with 3×10 mL of water

followed by Na_2SO_4 drying of the organic fraction. Trifluorotoluene was evaporate under reduced pressure. Crystallization from *n*-hexane yield 1.01 g (87%) of product as a white solid.

NMR analysis:

^{1}H NMR (600 MHz, $CDCl_3$, 300 K): δ: 7.30–7.32 (m); 7.36–7.38 (m).

^{13}C{H} NMR (150 MHz, $CDCl_3$, 300 K): δ: 29.4–30.0 (m); 129.6; 131.2; 136.6.

b. NMR spectra

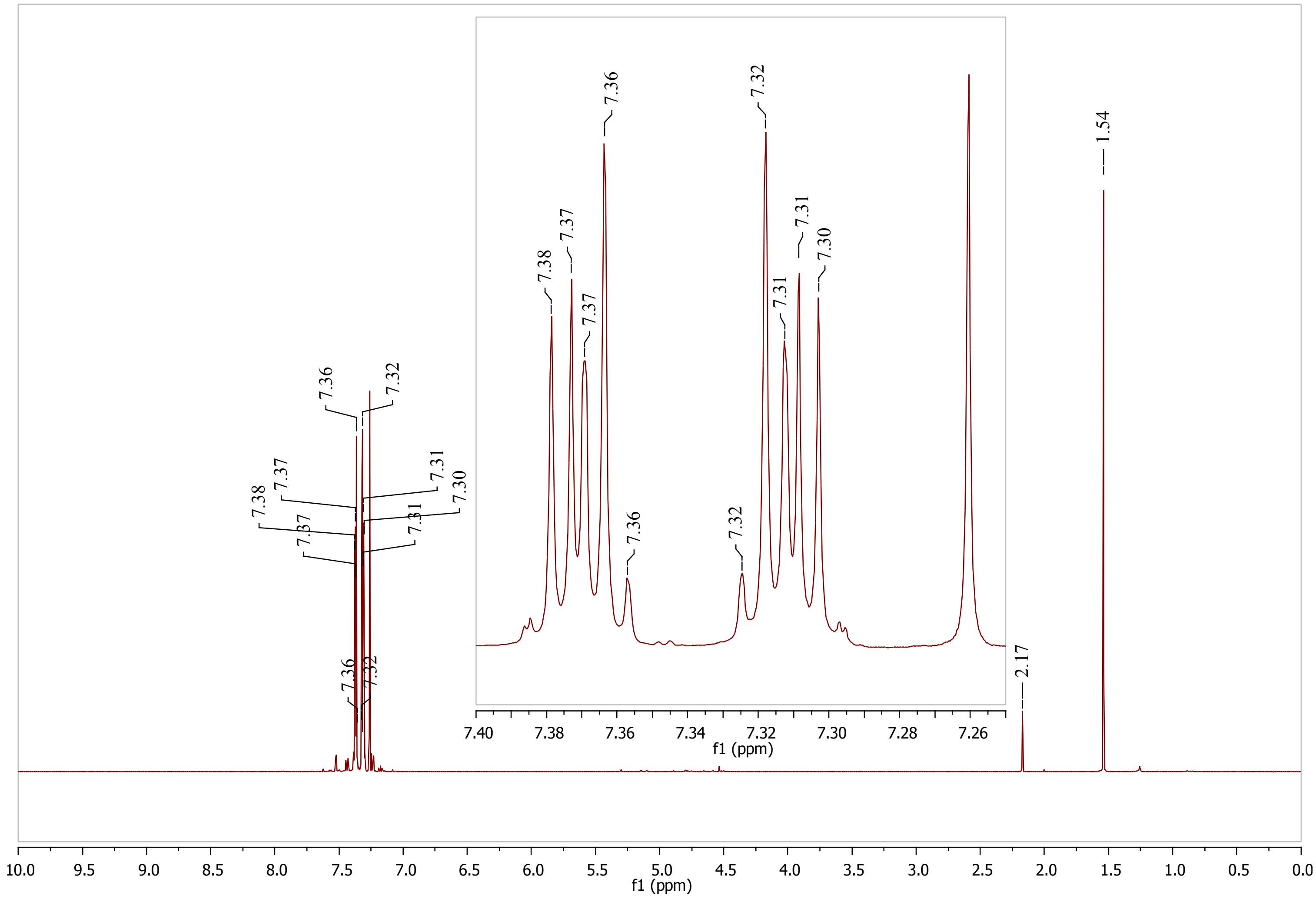

Fig. S33 ^{1}H NMR (600 MHz, $CDCl_3$, 300 K, σ) spectrum of the α,α'-dibromo-o-xylene[d_4]. 2.17 ppm – acetone; 1.54 ppm – water

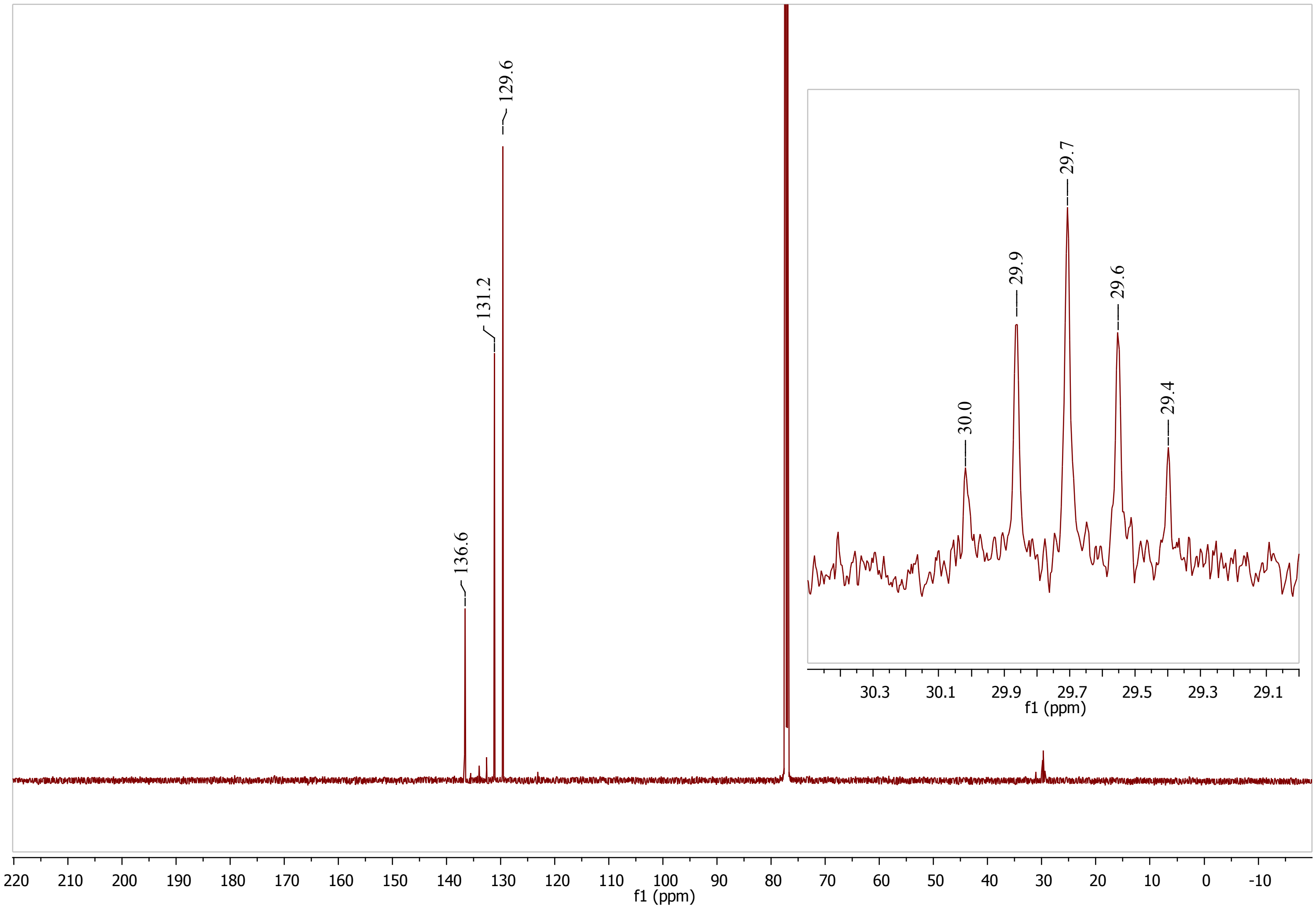

Fig. S34 ^{13}C NMR (150 MHz, $CDCl_3$, 300 K, σ) spectrum of the α,α'-dibromo-o-xylene[d_4]

7. Synthesis and analysis of the Fmoc–Oxa–OH[d_2]

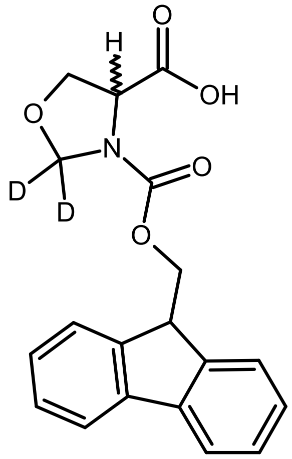

a. Synthesis

The deuterated isotopologue of the Fmoc-Oxa-OH was synthesized according to the modified procedure described by Falorni and co-workers.[3] A solution of 315 mg (3 mmol) of L-serine and 500 mL of formaldehyde[d_2] (~ 20 wt. % in D_2O; 98 atom % D) in 2N NaOH (50 mL) was allowed to stand overnight at 0°C. After this time 317 mg (3 mmol) of Na_2CO_3 and 50 mL of 1,4-dioxane were added at 0°C. Then 789 mg (3.05 mmol) of Fmoc-Cl in 10 mL of 1,4-dioxane was added dropwise (over the 1 h) under vigorous stirring. The reaction mixture was warm to room temperature and left for 23 hours. Next the reaction mixture was diluted with water, extracted with ether and the ether extracts were discarded. Ether extraction of acidified (3N HCl) aqueous phase, drying with Na_2SO_4 and evaporation of the solvent yield 820 mg (80%) of the pure compound as a colorless oil.

ESI-MS analysis:

Fmoc–Oxa–OH *m/z* 342.131 [calc. for $C_{19}H_{16}D_2NO_5$ 342.130] and *m/z* 364.114 [calc. for $C_{19}H_{15}D_2NNaO_5$ 364.112]

NMR analysis:

^{1}H NMR (500 MHz, CD_3CN, 300 K): δ: 4.10-4.12 (m, 1H); 4.19 (b, 1H); 4.26 (b, 1H); 4.36-4.41 (m, 1H); 7.34 (td, J = 1.0 Hz, 2H); 7.42 (t, J = 7.5 Hz, 2H); 7.63 (d, J = 7.4 Hz, 2H); 7.83 (d, J = 7.5 Hz, 2H).

^{13}C{H} NMR (125 MHz, CD_3CN, 300 K): δ: 48.0; 57.8; 57.8; 68.3; 71.1; 121.0; 126.1; 128.2; 128.8; 142,2; 145.0; 154.0; 171.9.

b. ESI-MS analysis

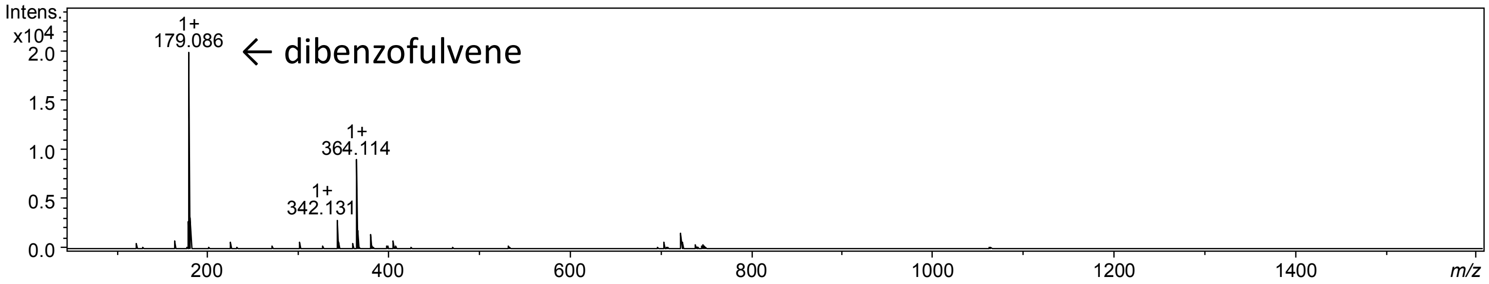

Fig. S35 ESI-MS spectrum of the Fmoc-Oxa-OH[d_2]

c. NMR analysis

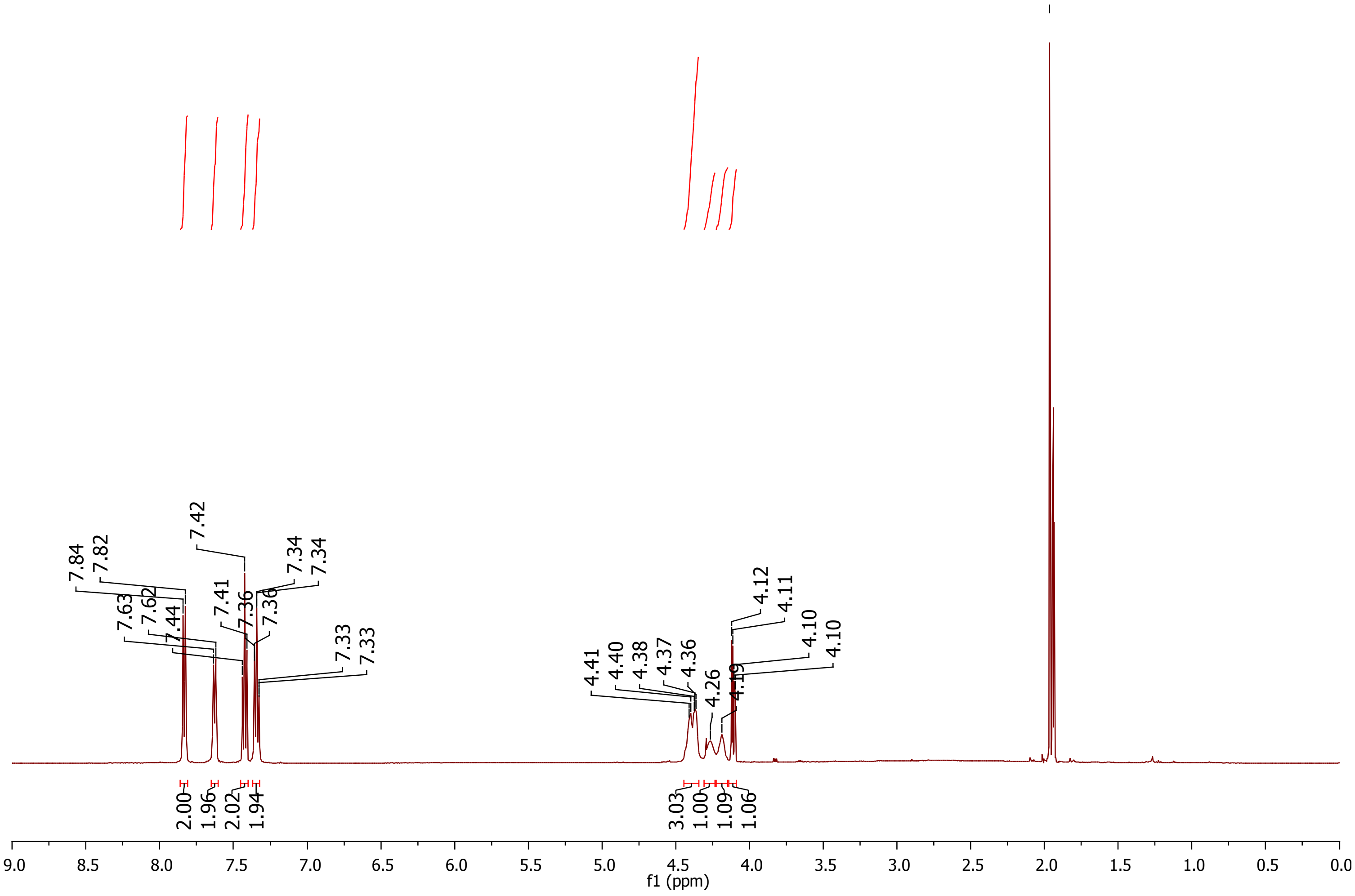

Fig. S36 ^{1}H NMR (500 MHz, CD_3CN, 300 K, σ) spectrum of the Fmoc-Oxa-OH[d_2]

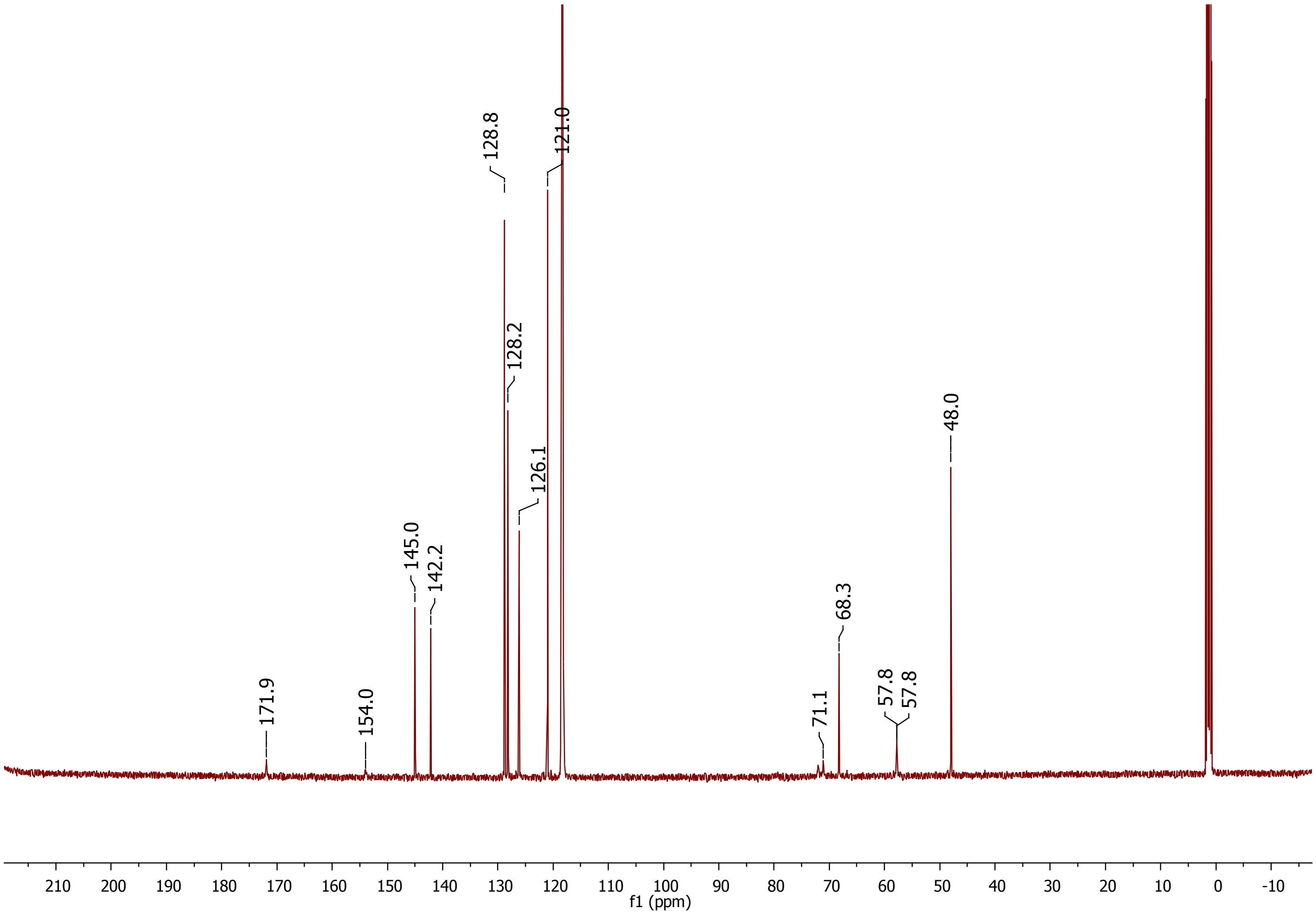

Fig. S37 ^{13}C NMR (125 MHz, CD_3CN, 300 K, σ) spectrum of the Fmoc-Oxa-OH[d_2]

8. Synthesis, ESI-MS and ESI-MS/MS analysis of deuterium labeled heterocyclic quaternary ammonium ionization tags

a. $BASN^{Oxa+}$–CO–4Abz–OH[d_1]

i. Synthesis

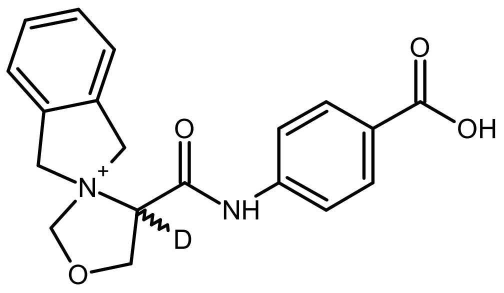

The synthesis of the $BASN^{Oxa+}$–CO–4Abz–OH[d_1] was performed on the preloaded 2-CT resin (4-aminobenzoic acid–2CT resin) according to established procedure (3.a). Single deuterium atom was introduced using hydrogen–deuterium exchange (HDX) reaction at the α-carbon atom[4,5]. Briefly: HDX was initiated by dissolving 0.1 mg of the tag in 200 μL of D_2O at room temperature. After 10 min, all protons from the amide bond as well as

carboxylic acid were exchanged by deuterons, as judged from ESI-MS analysis. Then, 2 μL of Et_3N was added at room temperature. After 15 min the mixture was analyzed by ESI-MS.

ESI-MS analysis:

BASN^{Oxa+}–CO–4Abz–OH[d_1] *m/z* 340.139 [calc. for $C_{19}H_{18}DN_2O_4$ 340.140]

ii. ESI-MS and ESI-MS/MS analysis

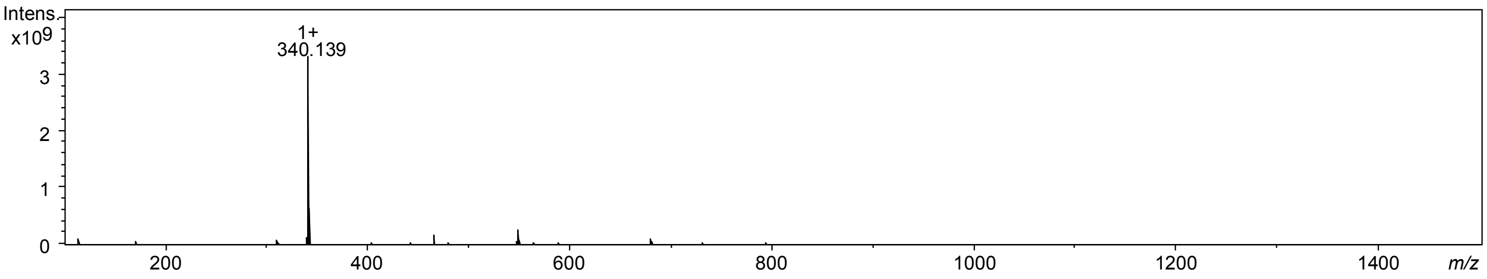

Fig. S38 ESI-MS spectrum of the BASN^{Oxa+}–CO–4Abz–OH[d_1]

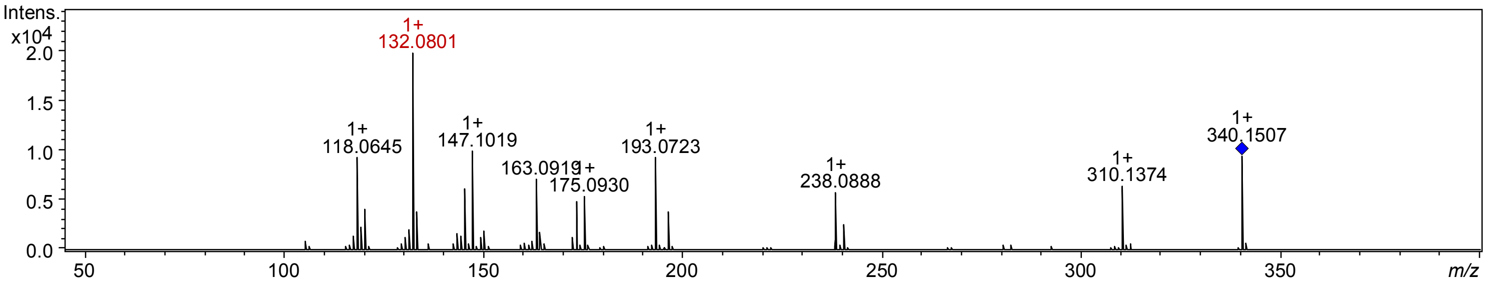

Fig. S39 ESI-MS/MS spectrum of the ion at *m/z* 340.150. Collision energy 20 V

b. BASN^{Oxa+}–CO–4Abz–OH[d_2]

i. Synthesis

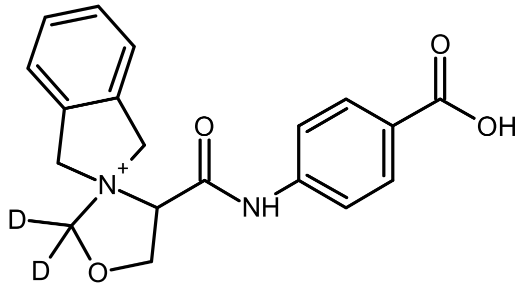

The synthesis of the BASN^{Oxa+}–CO–4Abz–OH[d_2] was performed on the preloaded 2-CT resin (4–aminobenzoic acid–2CT resin) according to established procedure (3.a). The deuterium atoms were introduced using previously prepared Fmoc–Oxa–OH[d_2] (7).

ESI-MS analysis:

BASN^{Oxa+}–CO–4Abz–OH[d_2] *m/z* 341.146 [calc. for $C_{19}H_{17}D_2N_2O_4$ 341.146]

ii. ESI-MS and ESI-MS/MS analysis

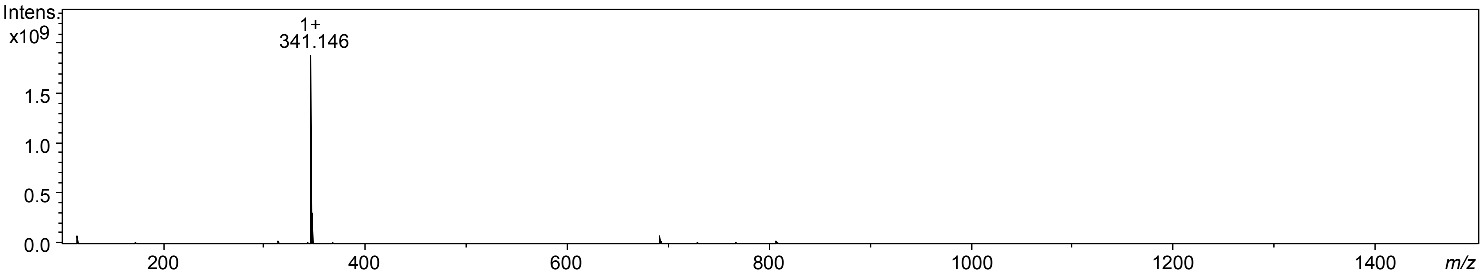

Fig. S40 ESI-MS spectrum of the BASN^{Oxa+}–CO–4Abz–OH[d_2]

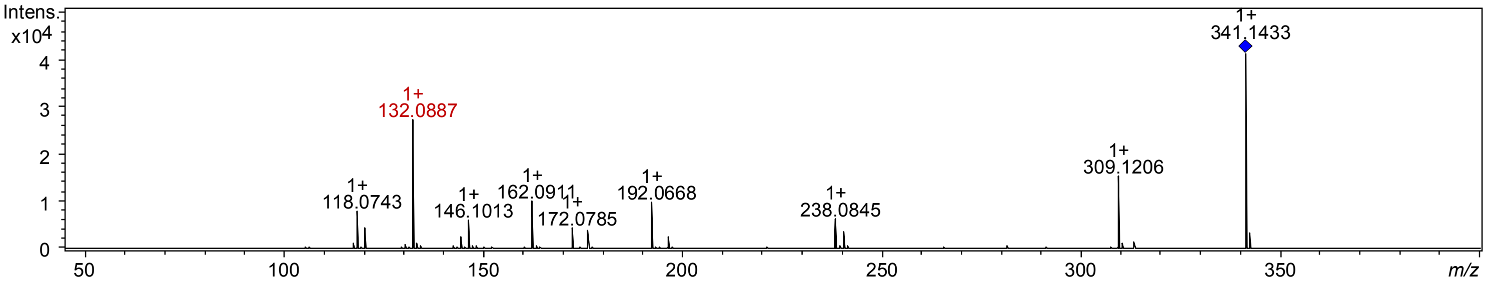

Fig. S41 ESI-MS/MS spectrum of the ion at *m/z* 341.143. Collision energy 15 V

c. BASN^{Oxa+}–CO–4Abz–OH[d_3]

i. Synthesis

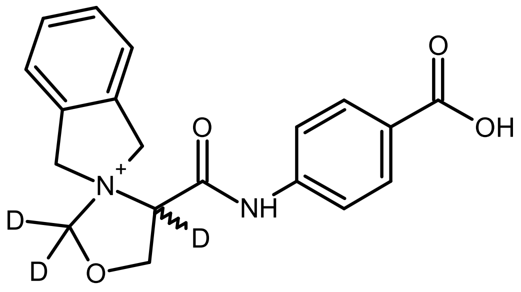

The synthesis of the BASN^{Oxa+}–CO–4Abz–OH[d_3] was performed on the preloaded 2-CT resin (4–aminobenzoic acid–2CT resin) according to established procedure (3.a) using previously prepared Fmoc–Oxa–OH[d_2] (7). Next compound BASN^{Oxa+}–CO–4Abz–OH[d_2] (b) was subjected to hydrogen–deuterium exchange (HDX) reaction at the α–carbon atom according to previously described protocol (a.i).

ESI-MS analysis:

BASN^{Oxa+}–CO–4Abz–OH[d_3] *m/z* 342.153 [calc. for $C_{19}H_{16}D_3N_2O_4$ 342.152]

ii. ESI-MS and ESI-MS/MS analysis

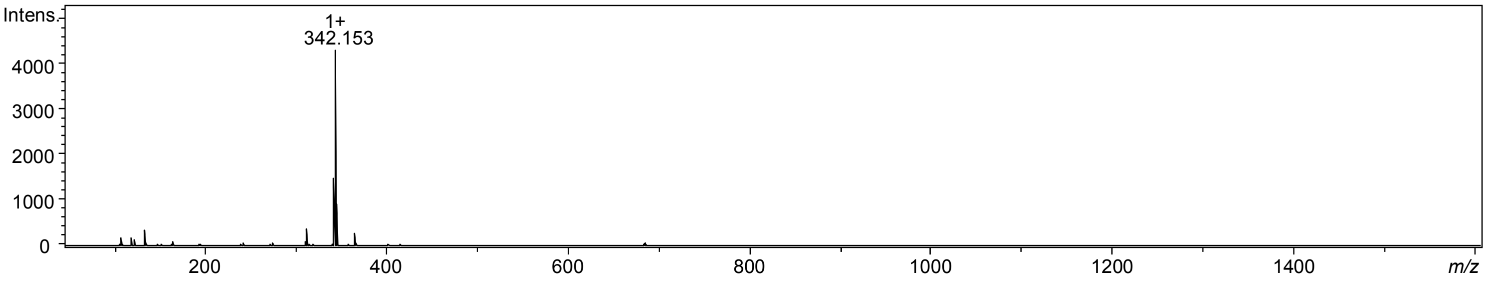

Fig. S42 ESI-MS spectrum of the BASN^{Oxa+}–CO–4Abz–OH[d_3]

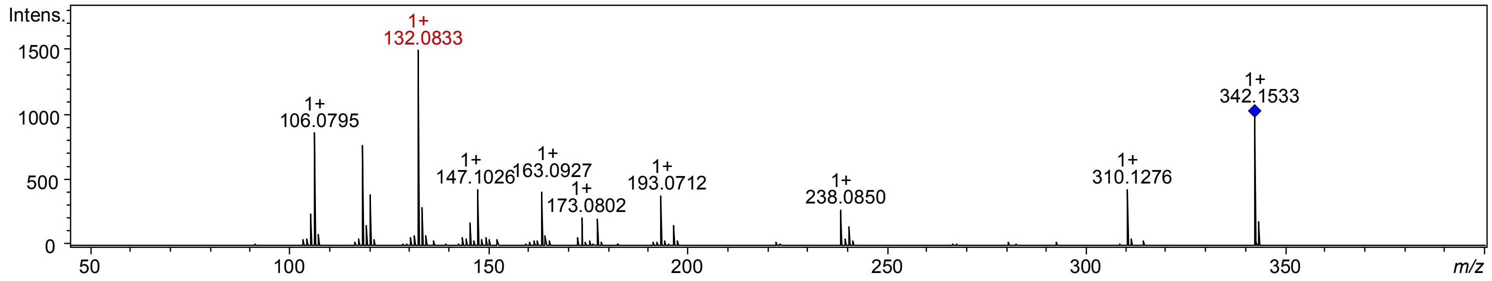

Fig. S43 ESI-MS/MS spectrum of the ion at *m/z* 342.153. Collision energy 20 V

d. BASN^{Oxa+}–CO–4Abz–OH[d$_4$]

i. Synthesis

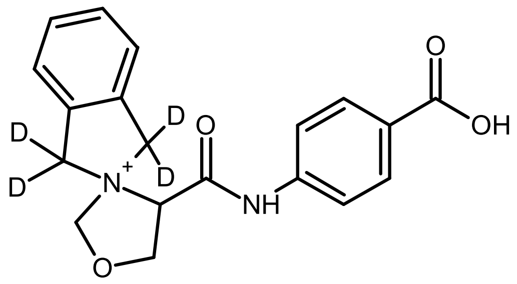

The synthesis of the BASN^{Oxa+}–CO–4Abz–OH[d$_4$] was performed on the preloaded 2-CT resin (4–aminobenzoic acid–2CT resin) according to established procedure (3.a). The deuterium atoms were introduced using previously prepared α,α'–dibromo–*o*–xylene[d$_4$] (6).

ESI-MS analysis:

BASN^{Oxa+}–CO–4Abz–OH[d$_4$] *m/z* 343.159 [calc. for $C_{19}H_{15}D_4N_2O_4$ 343.159]

NMR analysis:

^{1}H NMR (500 MHz, CD_3CN, 300 K): δ: 4.54 (dd, *J* = 5.7 Hz, 1H); 4.77 (dd, *J* = 7.9 Hz, 1H); 5.01 (dd, *J* = 5.3 Hz, 1H); 5.40 (dd, *J* = 5.8 Hz, 1H); 7.36-7.42 (m, 4H); 7.74-7.76 (m, 2H); 7.95-7.98 (m, 2H); 11.47 (s, 1H).

^{13}C{H} NMR (125 MHz, CD_3CN, 300 K): δ: 69.8; 71.0; 93.2; 120.4; 123.9; 124.1; 127.3; 129.8; 131.2; 132.8; 133.7; 142.4; 164.3; 167.1.

ii. ESI-MS and ESI-MS/MS analysis

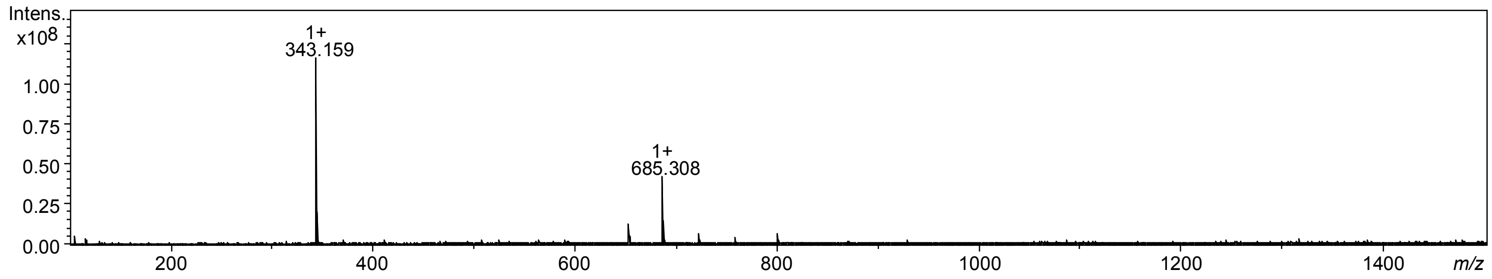

Fig. S44 ESI-MS spectrum of the BASN^{Oxa+}–CO–4Abz–OH[d$_4$]

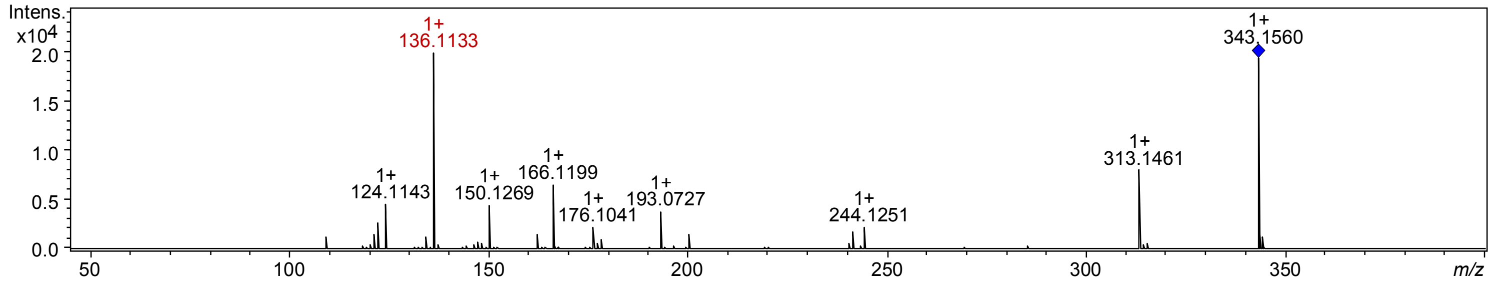

Fig. S45 ESI-MS/MS spectrum of the ion at *m/z* 343.156. Collision energy 15 V

iii. NMR analysis

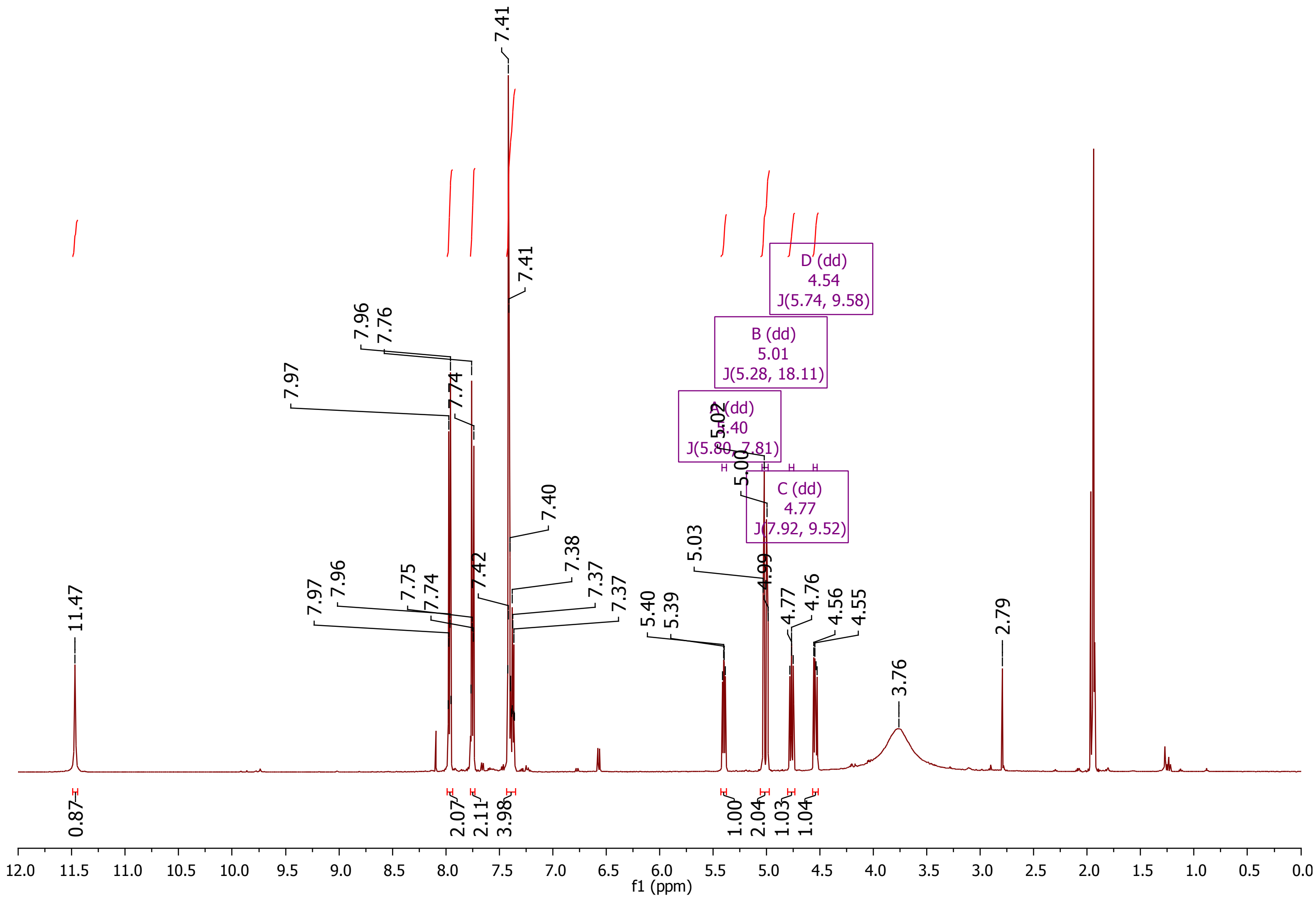

Fig. S46 ^{1}H NMR (500 MHz, CD_3CN, 300 K, σ) spectrum of the BASN^{Oxa+}–CO–4Abz–OH[d_4]

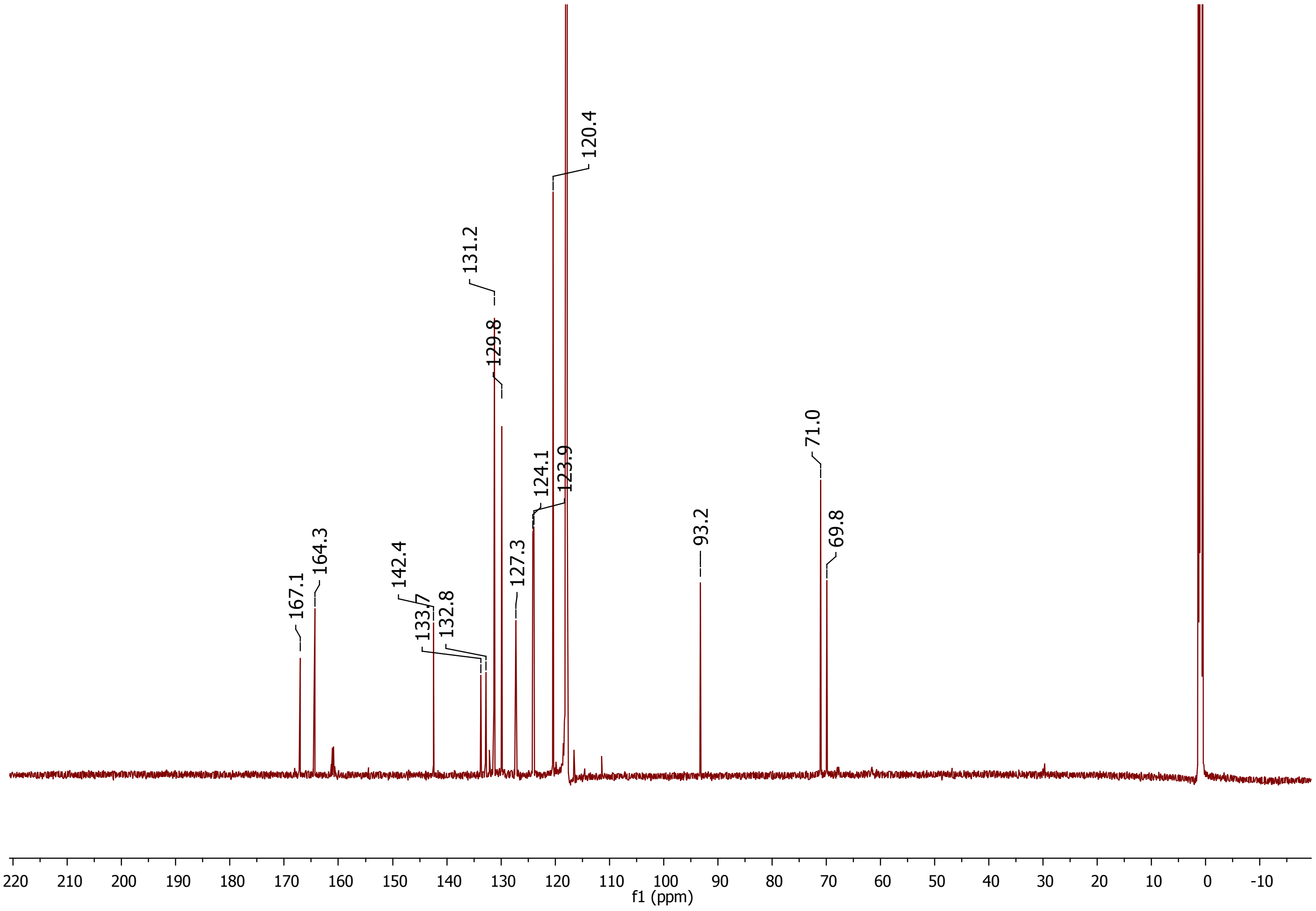

Fig. S47 ^{13}C NMR (125 MHz, CD_3CN, 300 K, σ) spectrum of the BASN^{Oxa+}–CO–4Abz–OH[d_4]

e. BASN^{Oxa+}–CO–4Abz–OPfp[d_4]

i. Synthesis

Active ester was prepared in solution using pentafluorophenyl trifluoroacetate reagent according to procedure previously described in the literature.[1]

ESI-MS analysis:

BASN^{Oxa+}–CO–4Abz–OPfp[d_4] *m/z* 509.148 [calc. for $C_{25}H_{14}D_4F_5N_2O_4$ 509.143]

ii. ESI-MS analysis

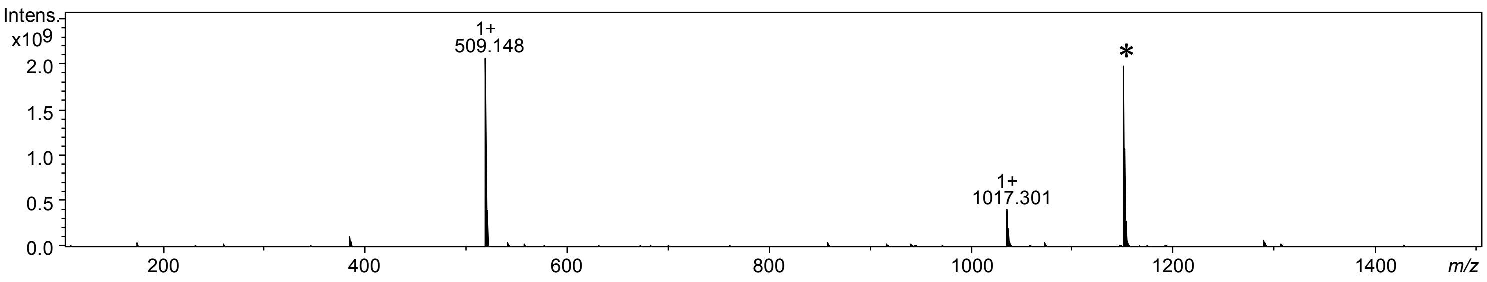

Fig. S48 ESI-MS spectrum of the BASN^{Oxa+}–CO–4Abz–OPfp[d$_4$]. * – impurity

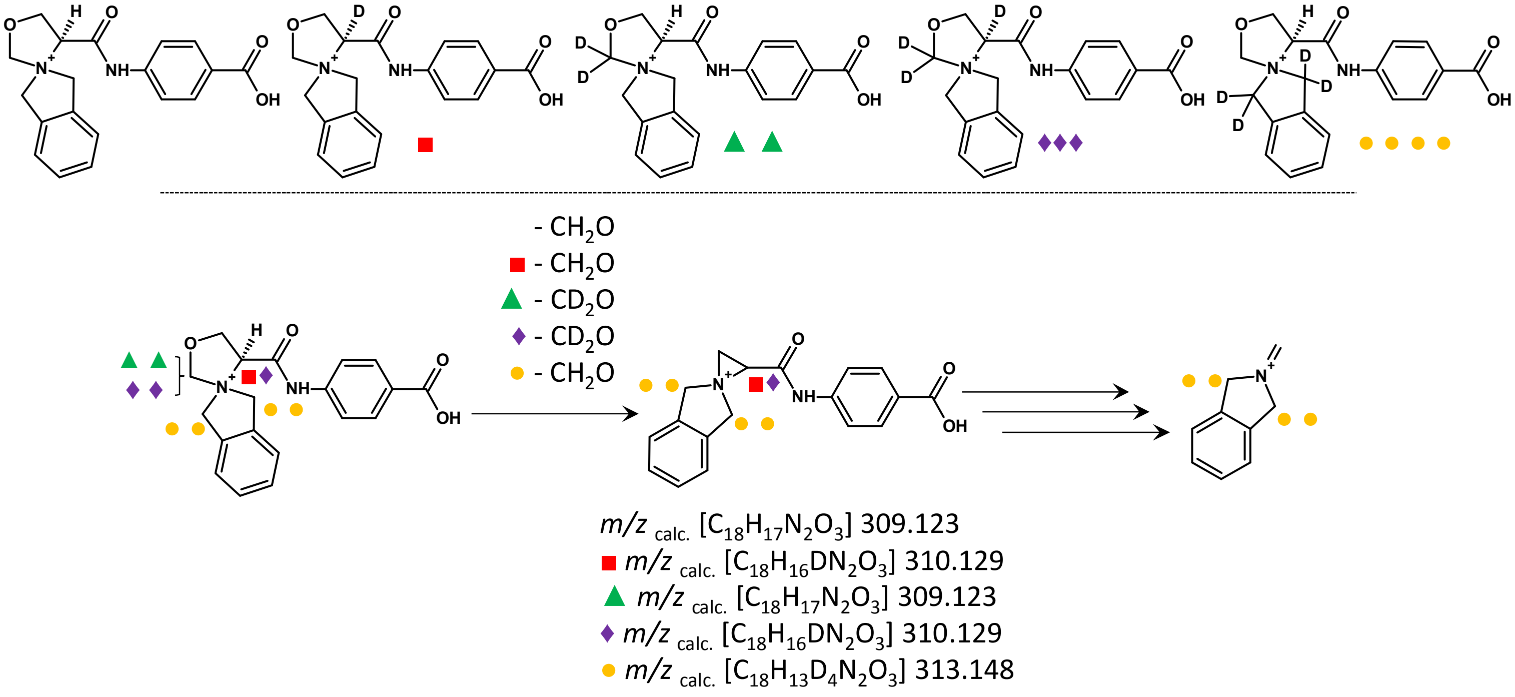

Scheme S5 Proposed fragmentation mechanism of the derivatization agent based on the MS/MS experiments (Figs. 18S., 39S., 41S., 43S., 45S.) of the obtained isotopomers

9. ESI-MS/MS analysis of the heterocyclic QAS derivatized model synthetic peptide LFTGHPETLEK[BASN^{Oxa+}{d$_4$}–CO–4Abz–]

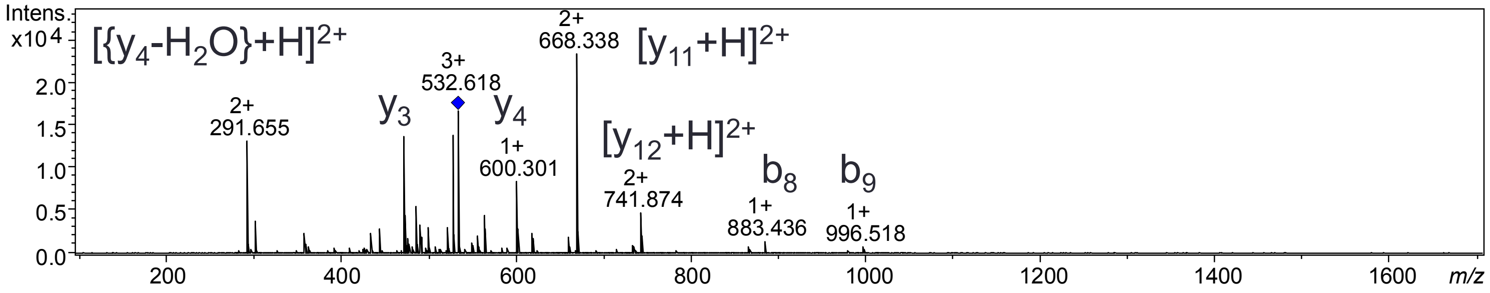

Fig. S49 ESI-MS/MS spectrum of the ion at *m/z* 532.618. Collision energy 15 V

10. LC-MS chromatogram of myoglobin tryptic digest derivatized with heterocyclic QAS

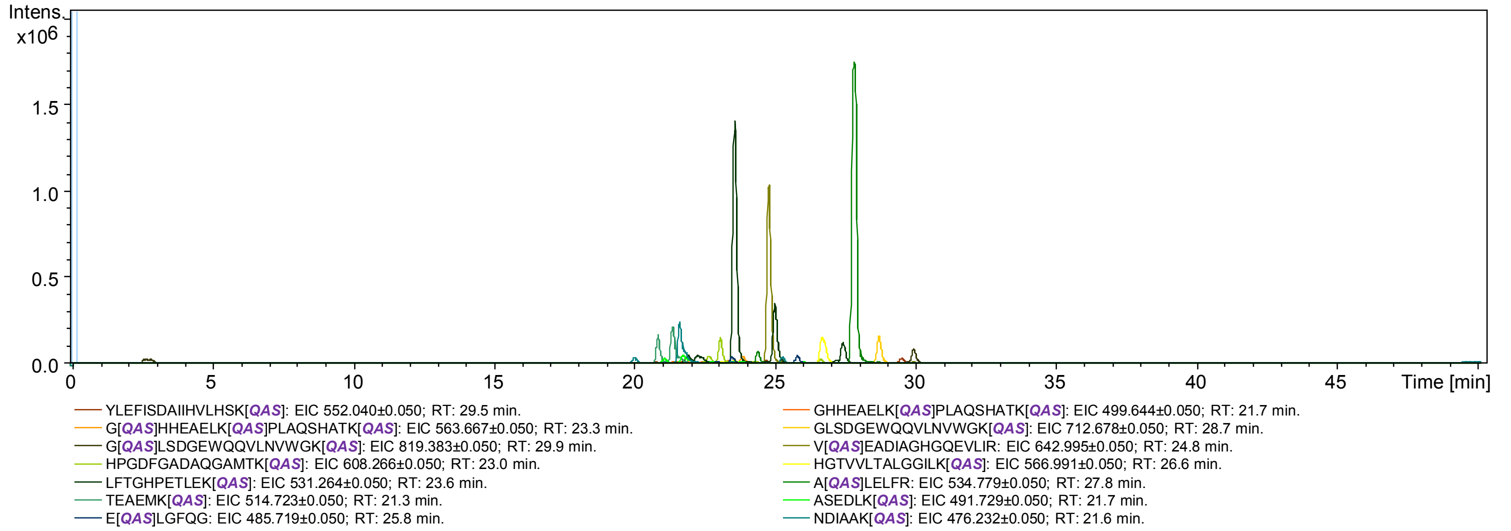

Fig. S50 LC-MS chromatogram of myoglobin tryptic digest derivatized with heterocyclic QA tag

Table S2 List of labeled peptides from myoglobin tryptic digest

n.o. = not observed

	Peptide sequence	Charge 1+	2+	3+	4+	5+	6+	RT [min]
		m/z found [*calculated*]						
1.	YLEFISDAIIHVLHSK[***QAS***]	*n.o.*	1103.124 [1103.072]	735.752 [735.717]	552.066 [552.040]	*n.o.*	*n.o.*	29.5
2.	GHHEAELK[***QAS***]PLAQSHATK[***QAS***]	*n.o.*	*n.o.*	*n.o.*	624.337 [624.304]	499.671 [499.644]	*n.o.*	21.7
	G[***QAS***]HHEAELK[***QAS***]PLAQSHATK[***QAS***]	*n.o.*	*n.o.*	*n.o.*	704.365 [704.332]	563.694 [563.667]	469.914 [469.891]	23.3
3.	GLSDGEWQQVLNVWGK[***QAS***]	*n.o.*	1068.568 [1068.513]	712.713 [712.678]	534.793 [534.760]			28.7
	G[***QAS***]LSDGEWQQVLNVWGK[***QAS***]	*n.o.*	1228.635 [1228.570]	819.424 [819.383]	614.818 [614.789]			29.9
4.	V[***QAS***]EADIAGHGQEVLIR	*n.o.*	964.039 [963.989]	643.028 [642.995]	482.534 [482.498]			24.8
5.	HPGDFGADAQGAMTK[***QAS***]	*n.o.*	911.944 [911.896]	608.298 [608.266]				23.0
6.	HGTVVLTALGGILK[***QAS***]	*n.o.*	850.026 [849.982]	567.019 [566.991]				26.6
7.	LFTGHPETLEK[***QAS***]	1591.865 [1591.778]	796.433 [796.393]	531.292 [531.264]				23.6
8.	A[***QAS***]LELFR	1068.608 [1068.550]	534.809 [534.779]	*n.o.*				27.8
9.	TEAEMK[***QAS***]	1028.496 [1028.439]	514.752 [514.723]	*n.o.*				21.3
10.	ASEDLK[***QAS***]	982.504 [982.451]	491.755 [491.729]	*n.o.*				21.7
11.	E[***QAS***]LGFQG	970.483 [970.430]	485.745 [485.719]	*n.o.*				25.8

12.	NDIAAK[***QAS***]	951.510 [951.456]	476.258 [476.232]	*n.o.*		21.6

11. SRM – quantification experiment using model synthetic peptide LFTGHPETLEK[BASN^{Oxa+}{d$_4$}–CO–4Abz–] and myoglobin tryptic digest

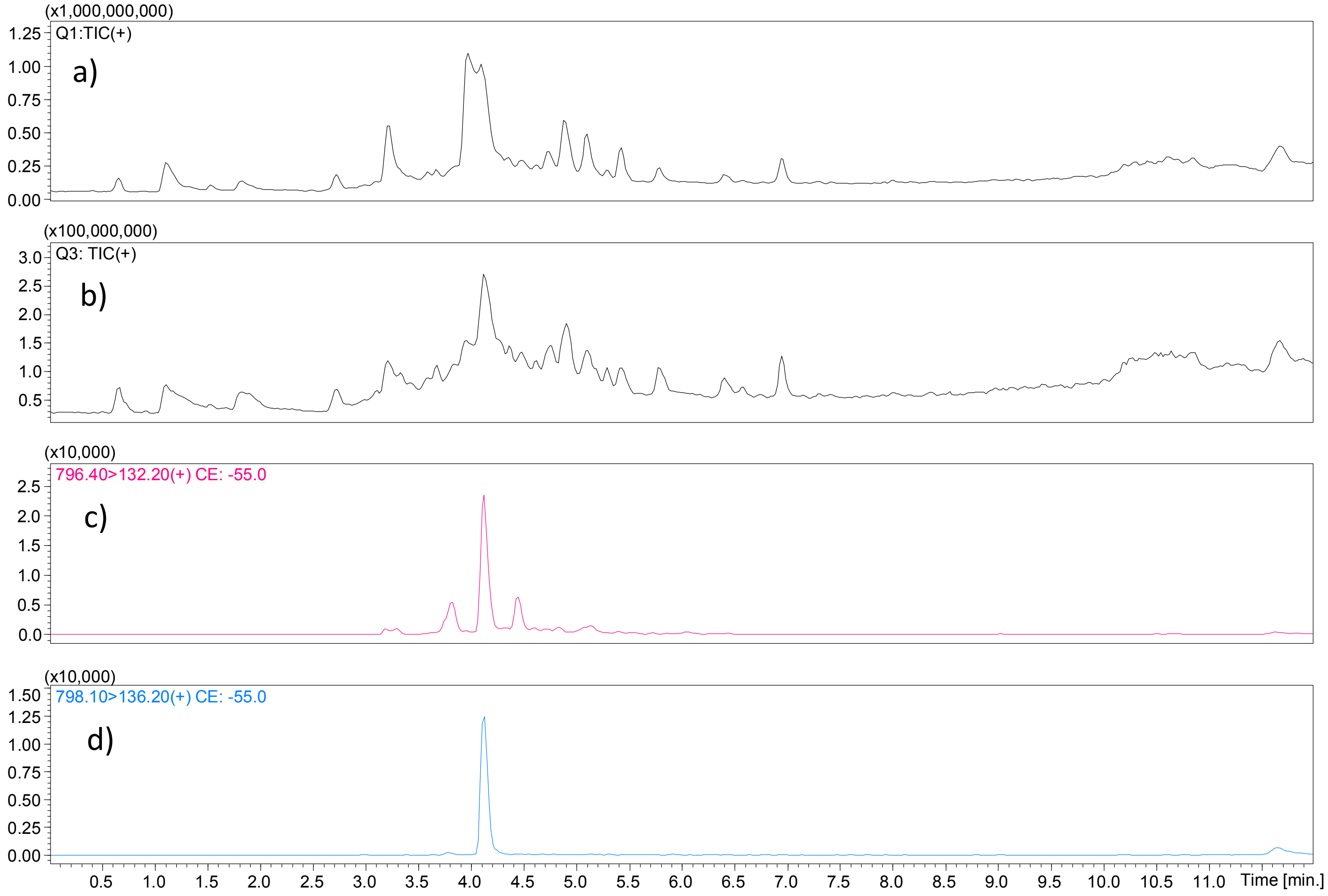

Fig. S51 (a) **Q1** chromatogram of the myoglobin tryptic digest mixed with model synthetic peptide LFTGHPETLEK[BASN^{Oxa+}{d$_4$}–CO–4Abz–]; (b) **Q3** chromatogram of the myoglobin tryptic digest mixed with the model synthetic peptide LFTGHPETLEK[BASN^{Oxa+}{d$_4$}–CO–4Abz–]; (c) LC-SRM chromatogram [*m/z* 796.40 to *m/z* 132.20(+) CE: -55.0]; (d) LC-SRM chromatogram [*m/z* 798.10 to *m/z* 136.20(+) CE: -55.0]

12. NMR spectra

a. ASN^{Thz+}–CO–DVYT–NH$_2$

^{1}H NMR (600 MHz, D$_2$O, 300 K) δ: 0,88 (dd, *J* = 3,4 Hz, 6H, Val, H$_\gamma$); 1.18 (d, *J* = 6,3 Hz, 3H, Thr, H$_\gamma$); 1.99–2.06 (m, 1H, Val, H$_\beta$); 2.27–2.29 (m, 4H, ASN$^{(Thz)+}$, H$_{\beta'}$ and H$_{\gamma'}$); 2.87 (ddd, *J* = 7,0 Hz, 2H, Tyr, H$_\beta$); 3.04 (ddd, *J* = 7,9 Hz, 2H, Asp, H$_\beta$); 3.41 (dd, *J* = 4,4 Hz, 1H, ASN$^{(Thz)+}$, H$_\beta$); 3.64–3.71 (m, 2H, ASN$^{(Thz)+}$, H$_\beta$ and H$_{\delta'}$); 3.82–3.96 (m, 3H, ASN$^{(Thz)+}$, H$_{\alpha'}$ and H$_{\delta'}$); 4.13 (d, *J* = 7,5 Hz, 1H, Val, H$_\alpha$); 4.22–4.25 (m, 1H, Thr, H$_\beta$); 4.28 (d, 1H, Thr, H$_\alpha$); 4.67–4.72 (m, 2H, Asp and ASN$^{(Thz)+}$, H$_\alpha$); 4.77 (dd, *J* = 10,0 Hz, 2H, ASN$^{(Thz)+}$, H$_\delta$); 4.83 (dd, *J* = 5,3 Hz, 1H, Tyr, H$_\alpha$); 6.85 (d, *J* = 8,3 Hz, 2H, Tyr, H$_\varepsilon$); 7.17 (d, *J* = 8,3 Hz, 2H, Tyr, H$_\delta$).

^{13}C{H} NMR (150 MHz, D_2O, 300 K) δ: 17.5 (Val, C_γ); 18.3 (Val, C_γ); 18.7 (Thr, C_γ); 21.6 (ASN$^{(Thz)+}$, $C_{\gamma'}$); 22.5 (ASN$^{(Thz)+}$, $C_{\beta'}$); 30.3 (Val, C_β); 30.3 (ASN$^{(Thz)+}$, C_β); 35.3 (Asp, C_β); 36.1 (Tyr, C_β); 50.4 (Tyr, C_α); 55.1 (Asp, C_α); 58.5 (Thr, C_β); 59.5 (Val, C_α); 60.5 (ASN$^{(Thz)+}$, $C_{\delta'}$); 63.8 (ASN$^{(Thz)+}$, C_β); 64.9 (Thr, C_α); 66.9 (ASN$^{(Thz)+}$, $C_{\alpha'}$); 73.2 (ASN$^{(Thz)+}$, C_α); 115.6 (Tyr, C_ε); 128.0 (Tyr, C_γ); 130.6 (Tyr, C_δ); 154.5 (Tyr, C_ζ); 165.7 (ASN$^{(Thz)+}$, CO); 171.3 (Asp, CO); 172.7 (Val, CO); 173.2 (Tyr, CO); 173.5 (Thr ,CO);174.0 (Asp, C_γO).

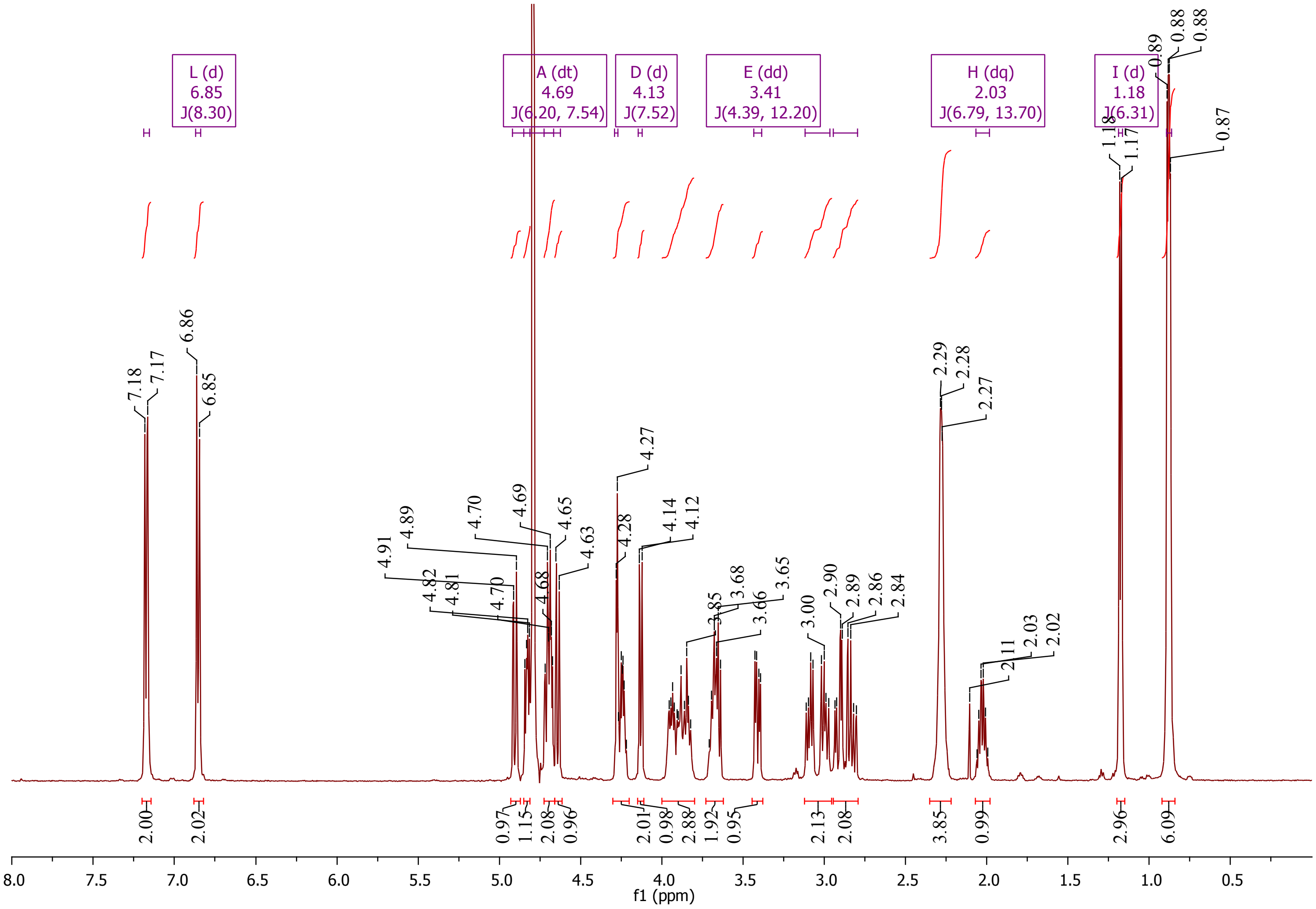

Fig. S52 ^{1}H NMR (600 MHz, D_2O, 300 K, σ) spectrum of the ASN^{Thz+}–CO–DVYT–NH_2

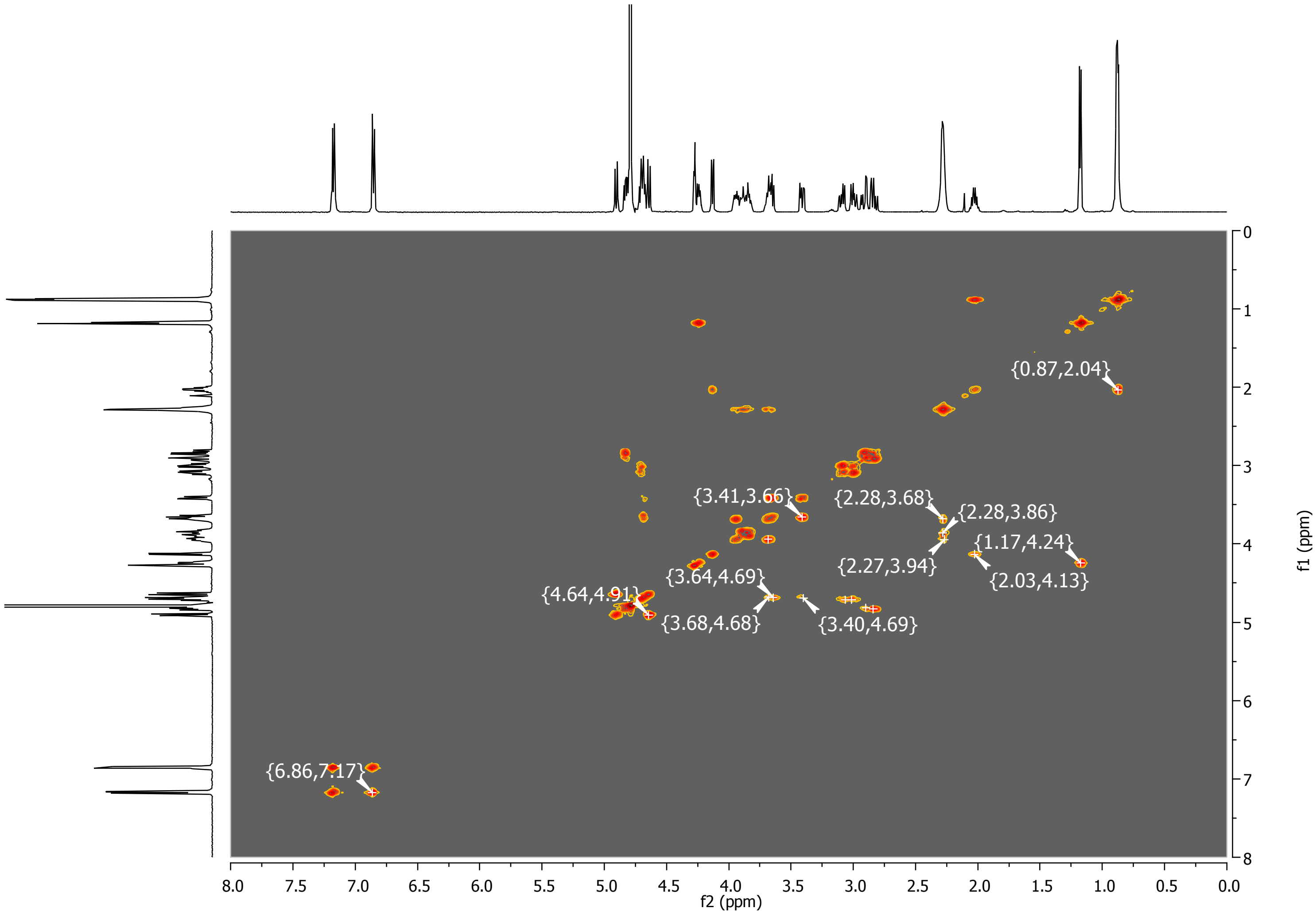

Fig. S53 COSY spectrum of the ASN^{Oxa+}–CO–DVYT–NH_2

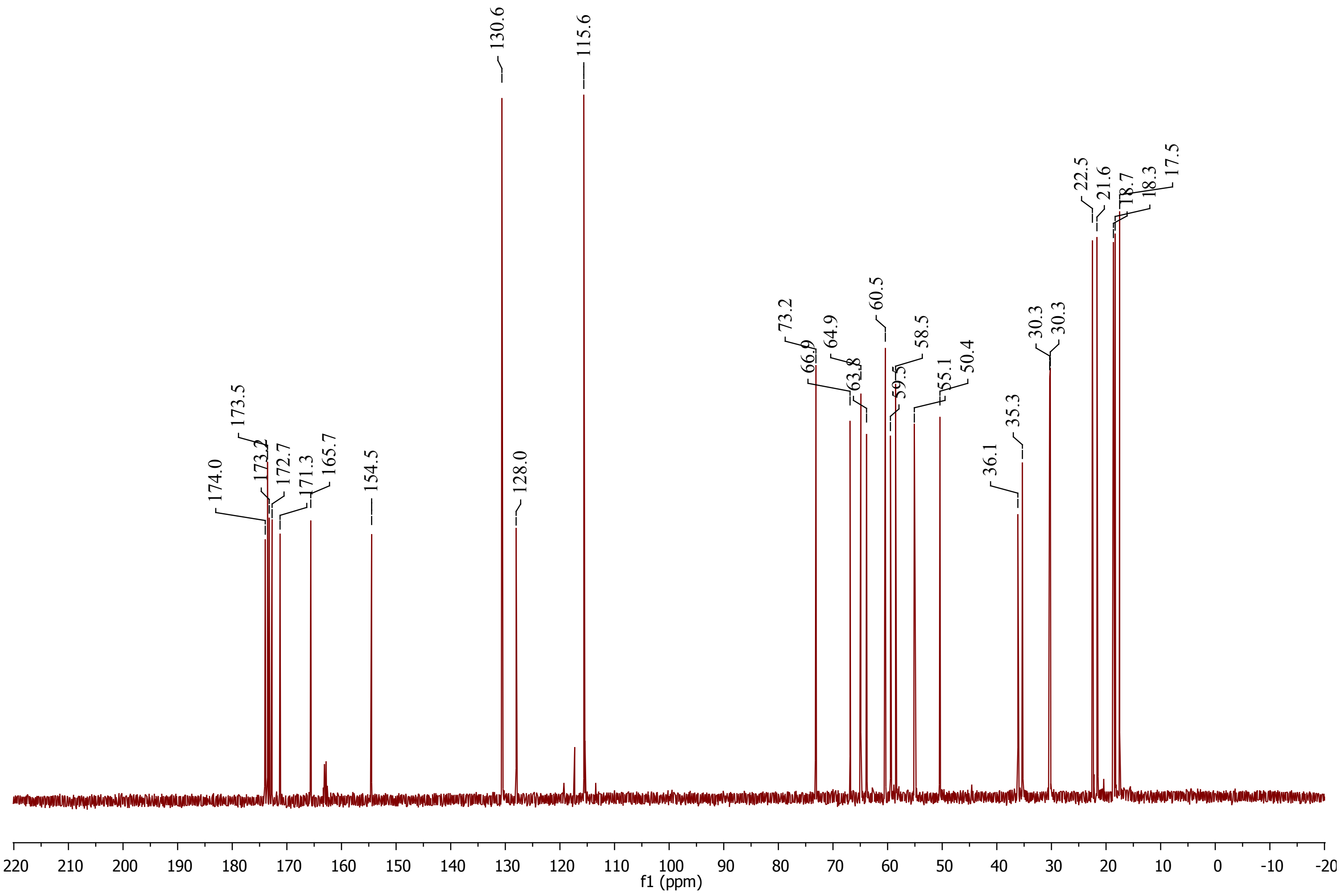

Fig. S54 ^{13}C NMR (150 MHz, D_2O, 300 K, σ) spectrum of the ASN^{Thz+}–CO–DVYT–NH_2

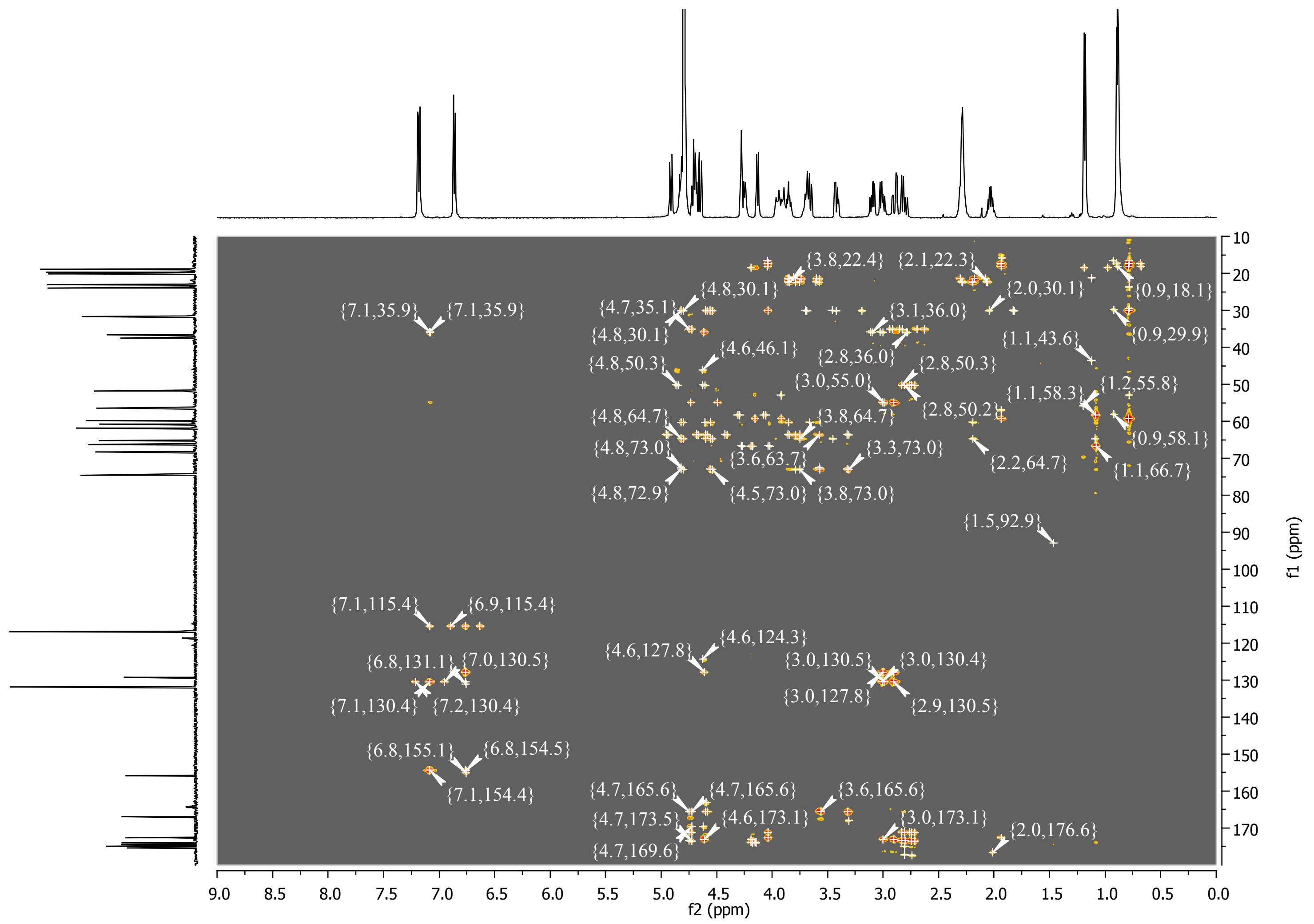

Fig. S55 HMBC spectrum of the ASN^{Oxa+}–CO–DVYT–NH$_2$

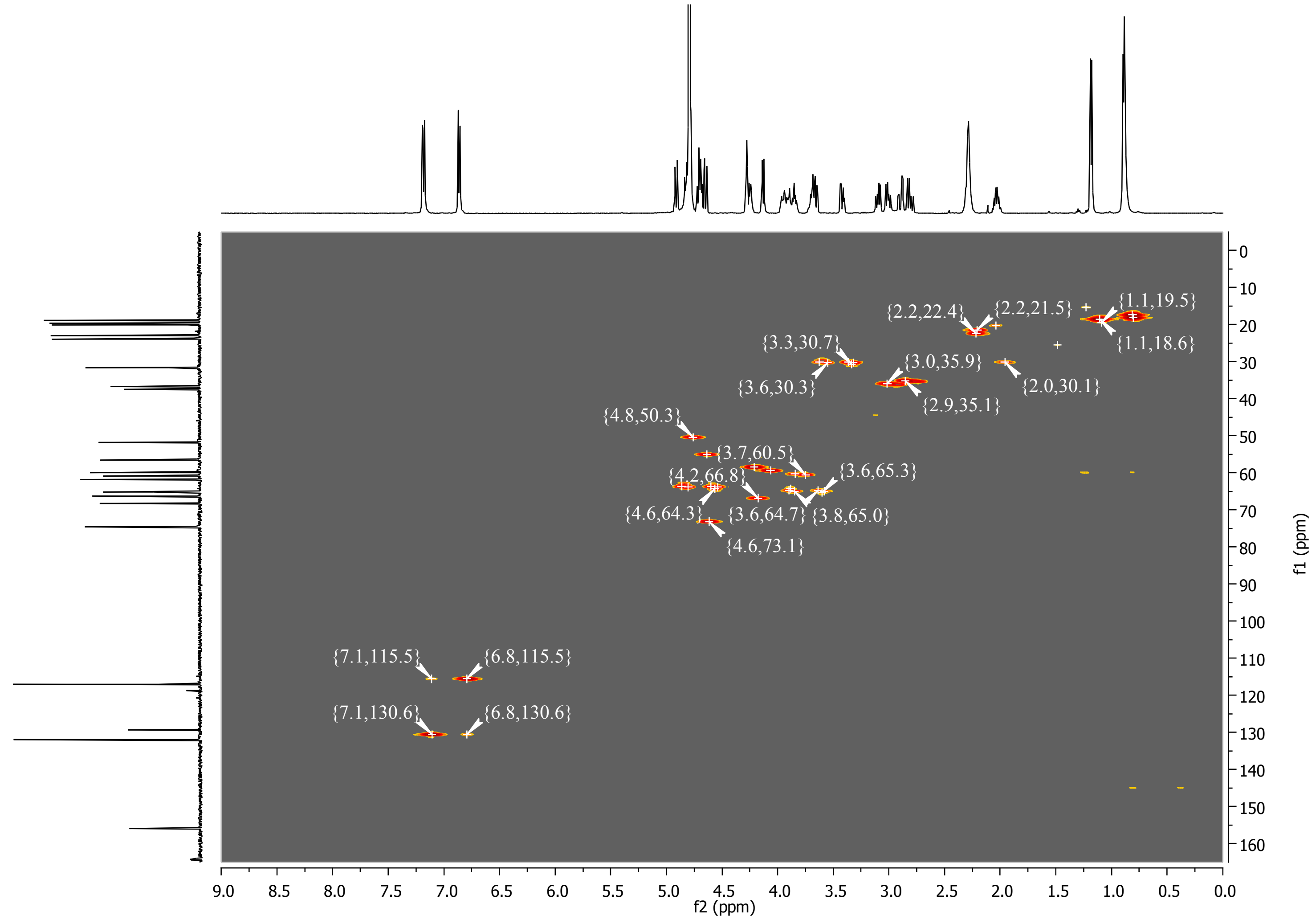

Fig. S56 HMQC spectrum of the ASN^{Thz+}–CO–DVYT–NH$_2$

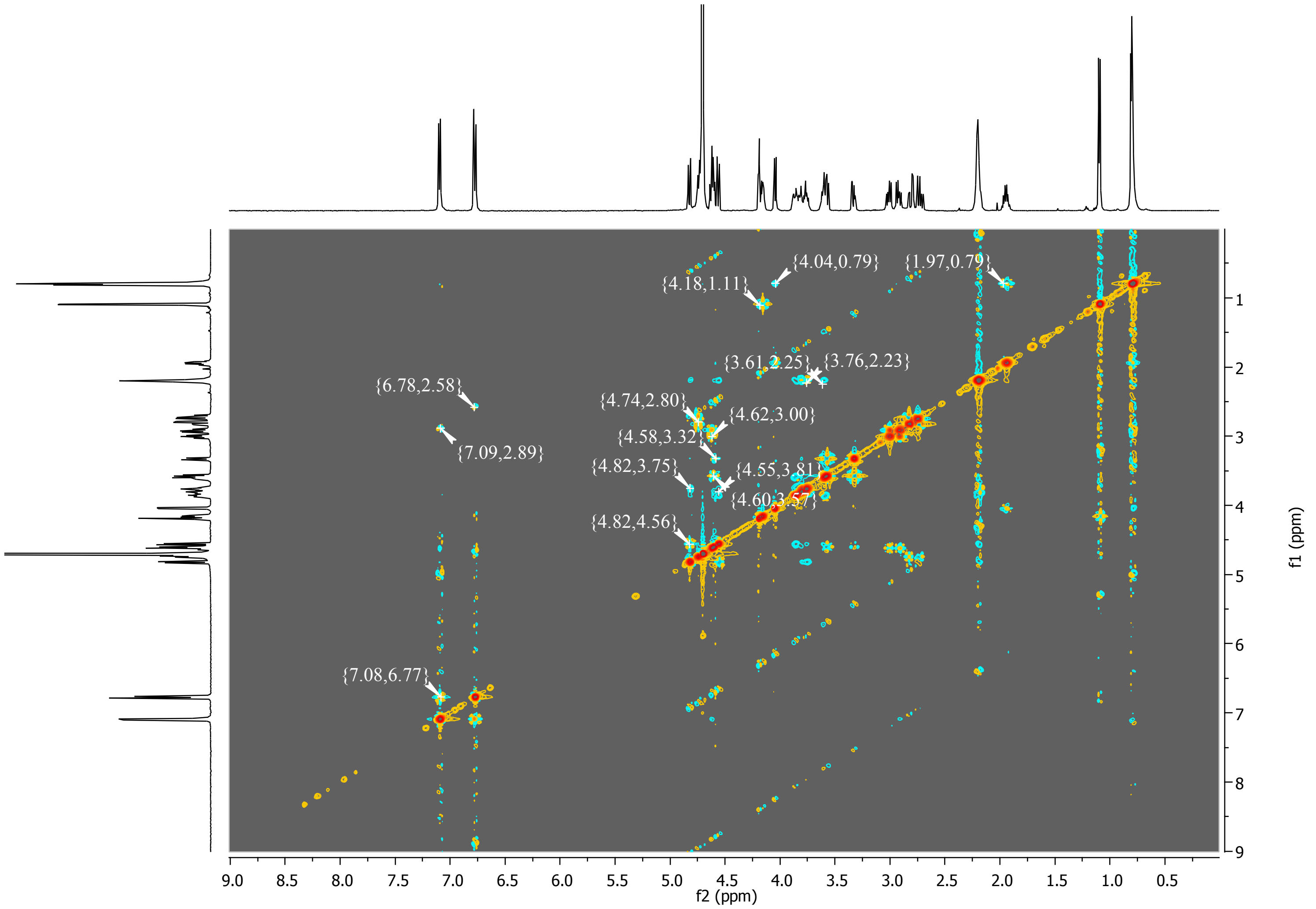

Fig. S57 NOESY spectrum of the ASN^{Thz+}–CO–DVYT–NH$_2$

b. BASN^{Thz+}–CO–DVYT–NH$_2$

^{1}H NMR (600 MHz, CD$_3$CN, 300 K) δ: 0.71 (dd, *J* = 6,9 Hz, 6H, Val, H$_\gamma$); 1.05 (d, *J* = 6,2 Hz, 3H, Thr, H$_\gamma$); 1.97–1.99 (m, 1H, Val, H$_\beta$); 2.71–2.87 (m, 3H, Asp, 2×H$_\beta$ and Tyr, 1×H$_\beta$); 3.06 (dd, *J* = 5,5 Hz, 1H, Tyr, 1×H$_\beta$); 3.56 (ddd, *J* = 6,7 Hz, 2H, BASN^{Thz+}, H$_\beta$); 3.98 (d, *J* = 5,9 Hz, 1H, Val, H$_\alpha$); 4.17–4.20 (m, 2H, Thr, H$_\beta$, H$_\alpha$); 4.48 (dd, *J* = 5,5 Hz, 1H, Tyr, H$_\alpha$); 4.65–4.68 (m, 2H, Asp, 1×H$_\alpha$, BASN^{Thz+}, 1×H$_\delta$); 4.88 (dd, *J* = 5,7 Hz, 1H, BASN^{Thz+}, H$_\alpha$); 4.95–5.04 (m, 3H, BASN^{Thz+}, 1×H$_\delta$, 1×H$_{\alpha'}$, 1×H$_{\delta'}$); 5.12 (d, *J* = 14,6 Hz, BASN^{Thz+}, H$_{\alpha'}$); 5.31 (d, *J* = 14,7 Hz, BASN^{Thz+}, H$_{\delta'}$); 6.68–6.71 (m, 2H, Tyr, H$_\varepsilon$); 7.03–7.07 (m, 2H, Tyr, H$_\delta$); 7.42–7.46 (m, 4H, BASN^{Oxa+}, Ar^o and Ar^m).

^{13}C{H} NMR (150 MHz, CD$_3$CN, 300 K) δ: 17.7 (Val, C$_\gamma$); 18.9 (Val, C$_\gamma$); 19.4 (Thr, C$_\gamma$); 30.4 (Val, C$_\beta$); 30.9 (BASN^{Thz+}, C$_\beta$); 35.8 (Asp, C$_\beta$); 36.4 (Tyr, C$_\beta$); 50.7 (Asp, C$_\alpha$); 56.1 (Tyr, C$_\alpha$); 58.9 (Thr, C$_\beta$); 60.5 (Val, C$_\alpha$); 65.3 (BASN^{Thz+}, C$_{\delta'}$); 66.0 (Thr, C$_\alpha$); 67.3 (BASN^{Thz+}, C$_{\alpha'}$); 69.3 (BASN^{Thz+}, C$_\alpha$); 73.8 (BASN^{Thz+}, C$_\delta$); 115.9 (Tyr, C$_\varepsilon$); 123.9 (BASN^{Thz+}, Ar^o); 124.0 (BASN^{Thz+}, Ar^o); 128.6 (Tyr, C$_\gamma$); 129.9 (BASN^{Thz+}, Ar^m); 130.0 (BASN^{Thz+}, Ar^m); 130.9 (Tyr, C$_\delta$); 132.5 (BASN^{Thz+}, C$_{\beta'}$); 133.2 (BASN^{Thz+}, C$_{\gamma'}$); 156.3 (Tyr, C$_\zeta$); 165.0 (BASN^{Thz+}, CO); 171.5 (Asp, CO); 172.5 (Val, CO); 172.9 (Tyr, CO), 173.5 (Thr ,CO); 173.9 (Asp, C$_\gamma$O).

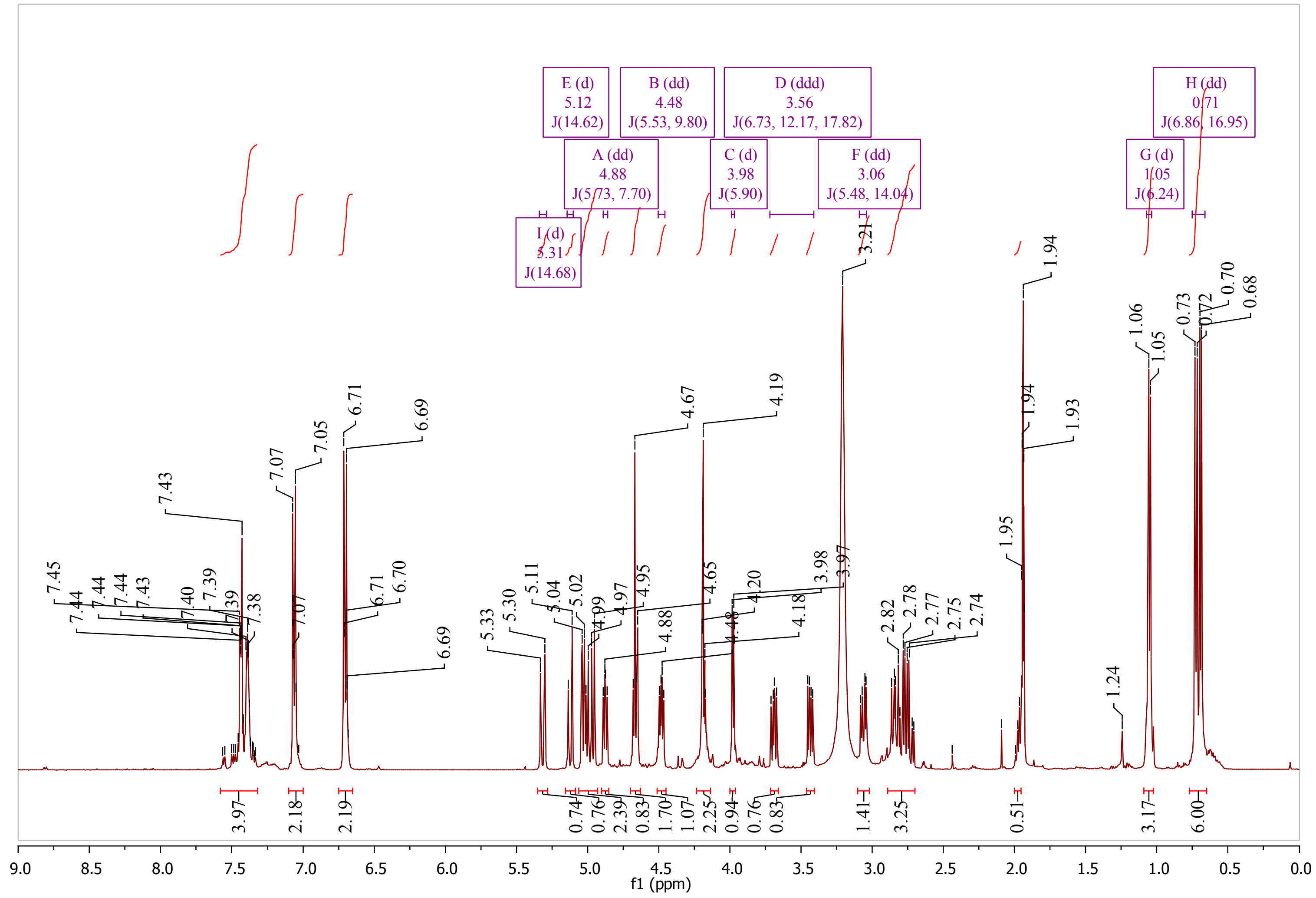

Fig. S58 ^{1}H NMR (600 MHz, CD_3CN, 300 K, σ) spectrum of the BASN^{Thz+}–CO–DVYT–NH_2

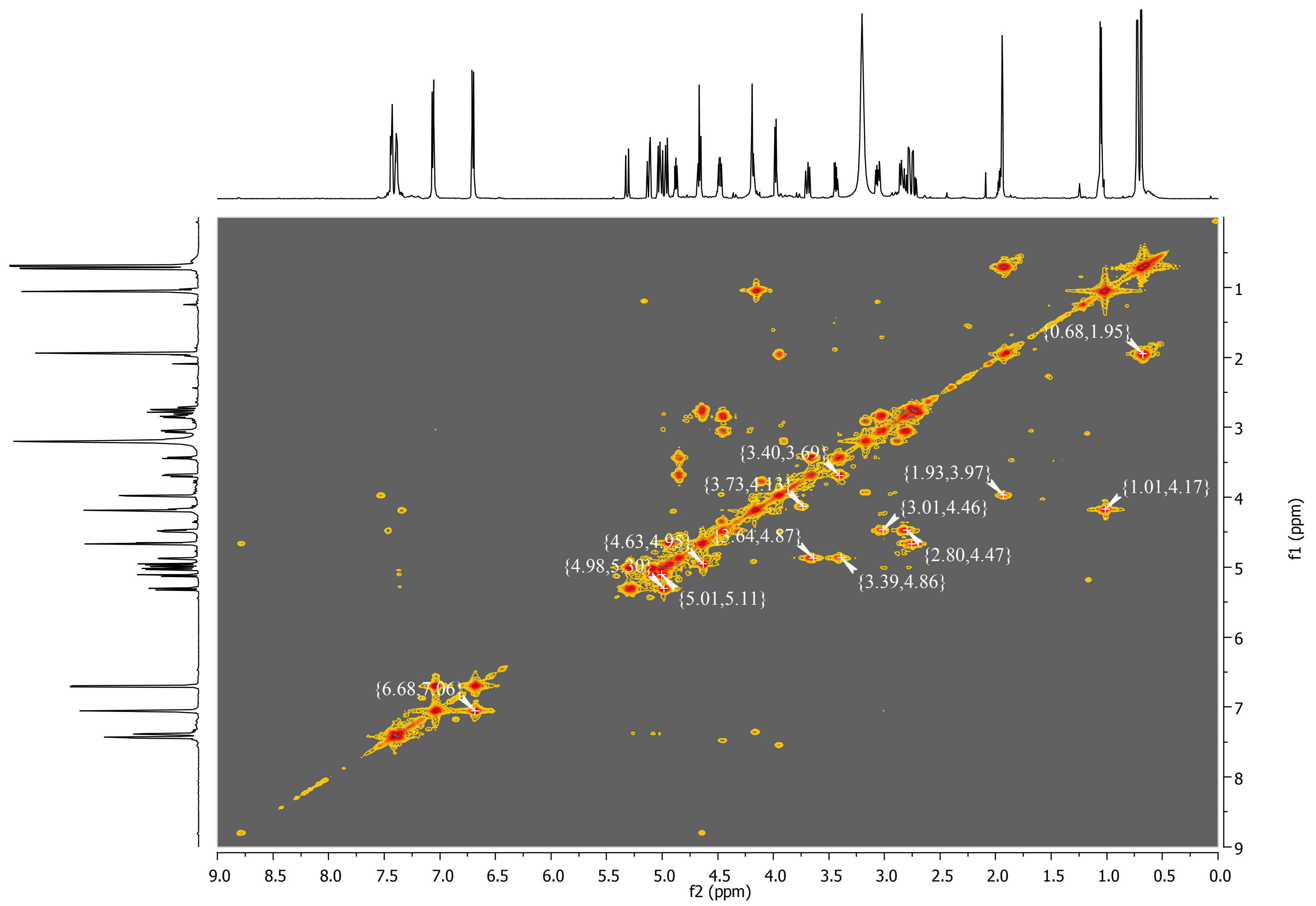

Fig. S59 COSY spectrum of the $BASN^{Thz+}$–CO–DVYT–NH_2

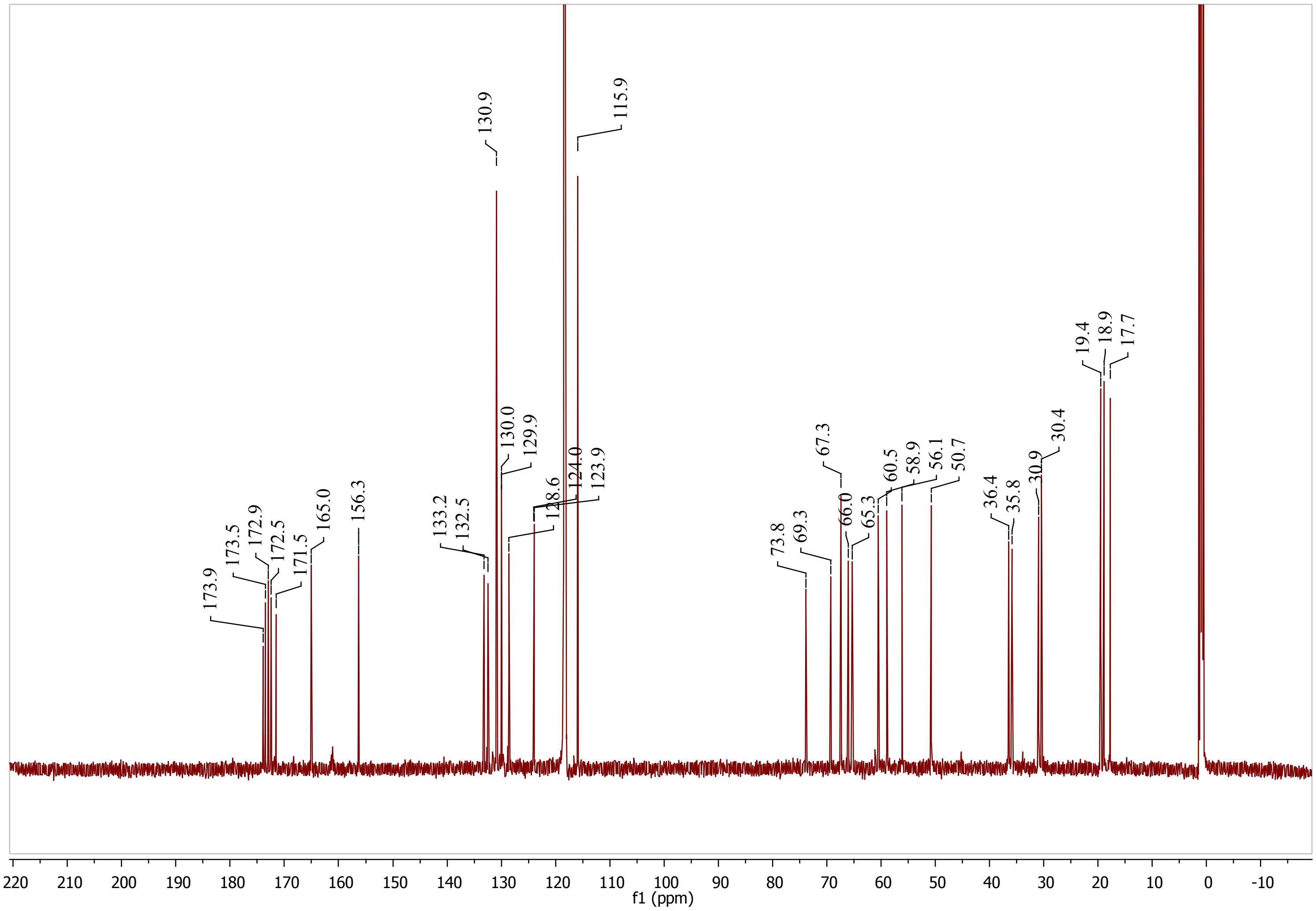

Fig. S60 ^{13}C NMR (150 MHz, CD_3CN, 300 K, σ) spectrum of the BASN^{Thz+}–CO–DVYT–NH_2

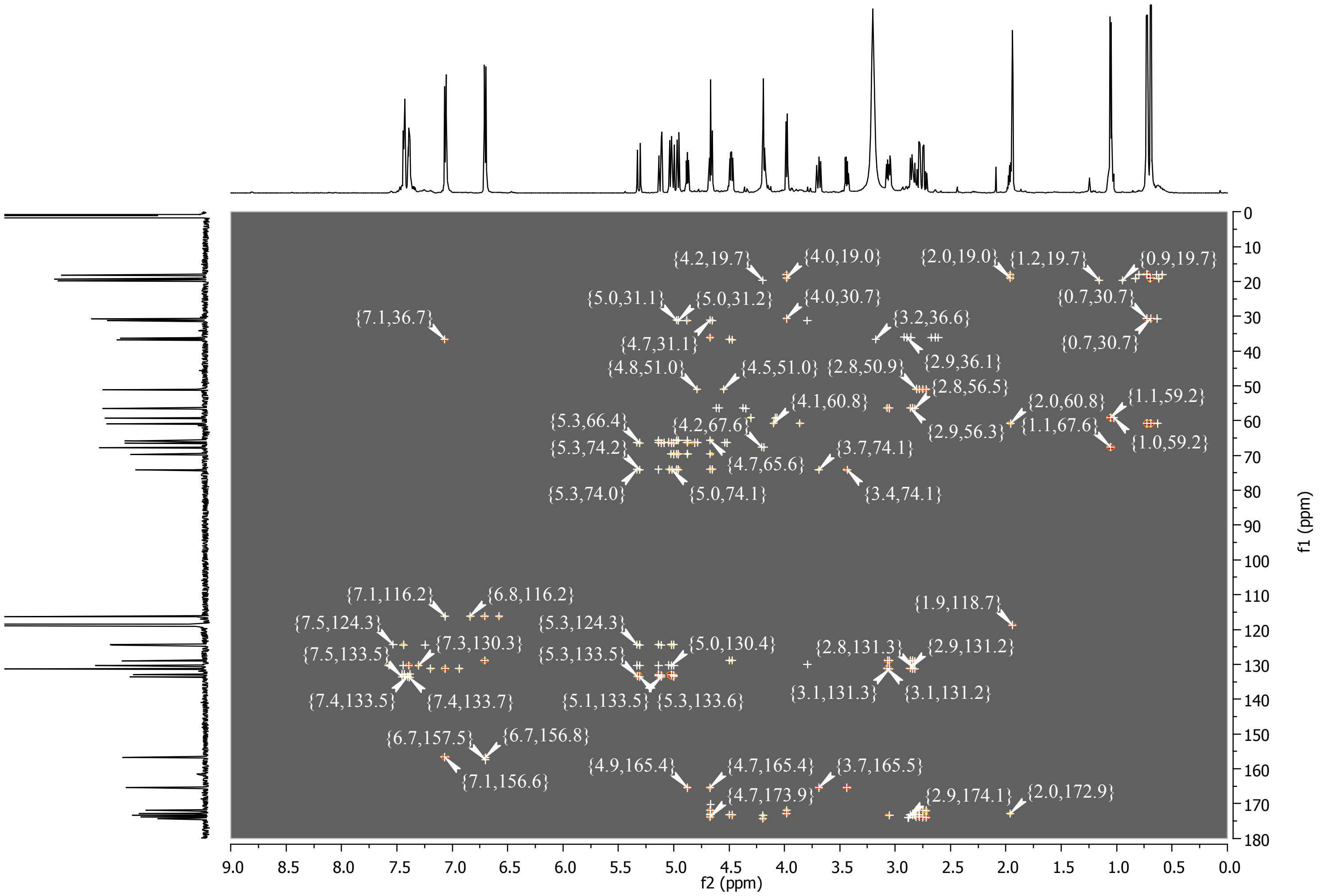

Fig. S61 HMBC spectrum of the BASN^{Thz+}–CO–DVYT–NH_2

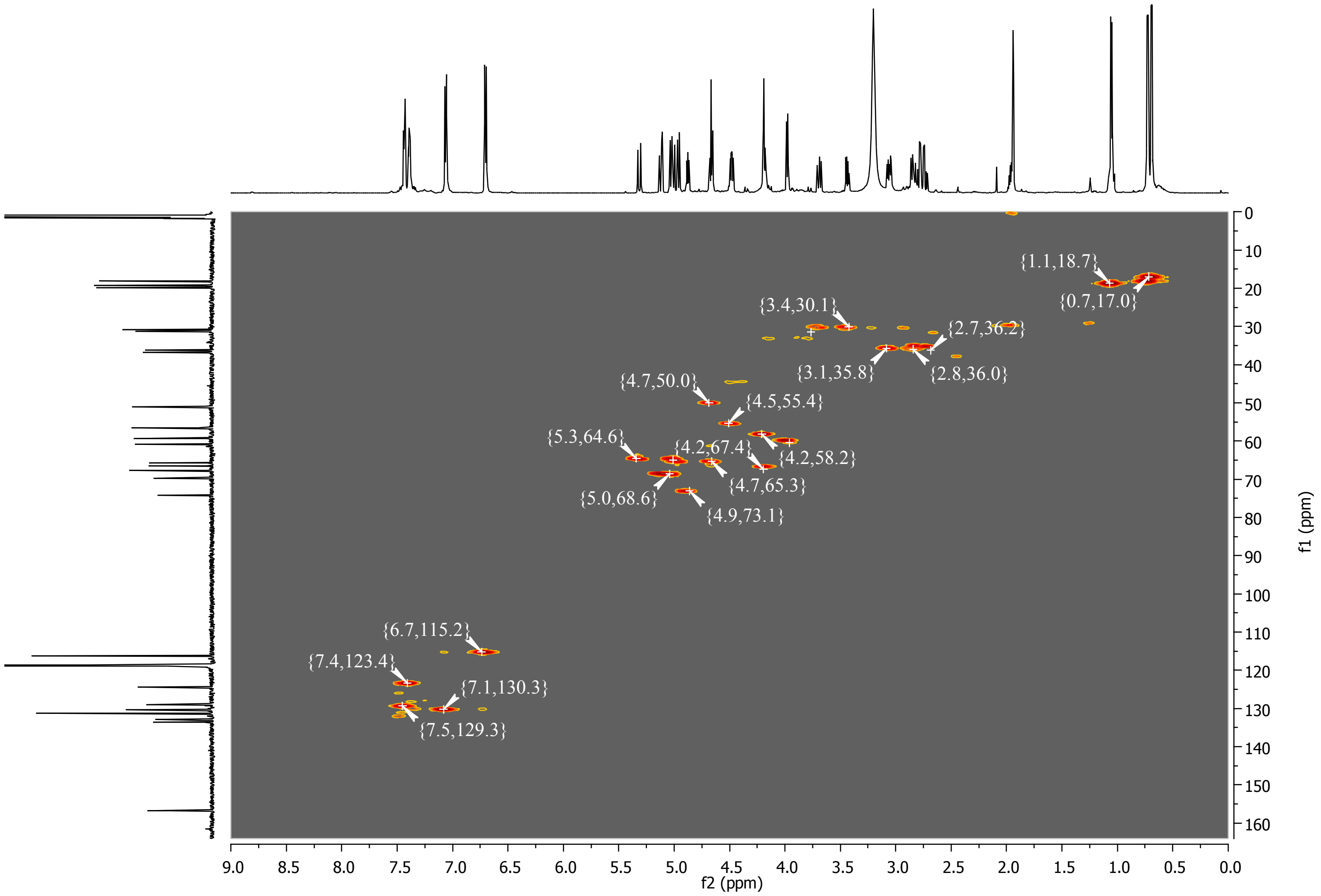

Fig. S62 HMQC spectrum of the BASN^{Thz+}–CO–DVYT–NH$_2$

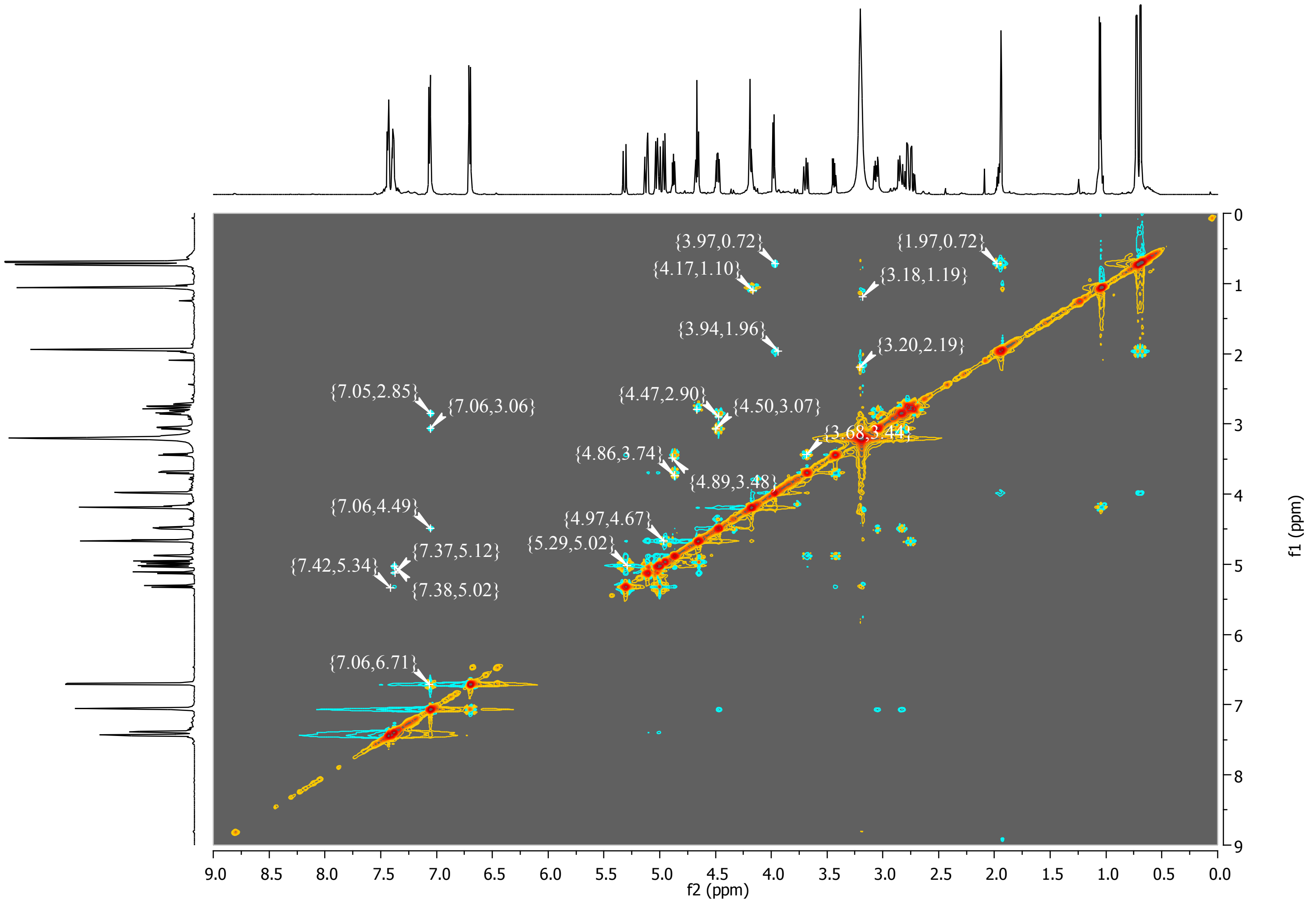

Fig. S63 NOESY spectrum of the BASN^{Thz+}–CO–DVYT–NH$_2$

c. *ASN^{Oxa+}–CO–DVYT–NH$_2$*

^{1}H NMR (600 MHz, CD_3CN/D_2O (9:1), 300 K) δ: 0.72 (dd, J = 6,9 Hz, 6H, Val, H_γ); 1.07 (d, J = 6,4 Hz, 3H, Thr, H_γ); 1.96–2.00 (m, 1H, Val, H_β); 2.01–2.24 (m, 4H, ASN$^{(Oxa)+}$, $H_{\beta'}$ and $H_{\gamma'}$); 2.85–2.95 (m, 2H, Tyr, H_β); 2.95 (ddd, J = 4,1 Hz, 2H, Asp, H_β); 3.50–3.54 (m, 1H, ASN$^{(Oxa)+}$, $H_{\alpha'}$); 3.56–3.62 (m, 2H, ASN$^{(Oxa)+}$, $H_{\delta'}$); 3.70–3.75 (m, 1H, ASN$^{(Oxa)+}$, $H_{\alpha'}$); 3.97 (d, J = 6,0 Hz, 1H, Val, H_α); 4.17–4.21 (m, 2H, Thr, H_α and H_β); 4.42 (ddd, J = 6,4 Hz, 2H, ASN$^{(Oxa)+}$, H_β); 4.47–4.48 (m, 1H, ASN$^{(Oxa)+}$, H_α); 4.49–4.50 (m, 1H, Tyr, H_α); 4.77 (t, J = 6,5 Hz, 1H, Asp, H_α); 4.87 (dd, J = 5,1 Hz, 2H, ASN$^{(Oxa)+}$, H_δ); 6.71 (d, J = 8,5 Hz, 2H, Tyr, H_ε); 7.07 (d, J = 8,5 Hz, 2H, Tyr, H_δ).

^{13}C{H} NMR (150 MHz, CD_3CN/D_2O (9:1), 300 K) δ: 17.7 (Val, C_γ); 18.7 (Val, C_γ); 19.3 (Thr, C_γ); 21.5 (ASN$^{(Oxa)+}$, $C_{\gamma'}$); 22.3 (ASN$^{(Oxa)+}$, $C_{\beta'}$); 30.3 (Val, C_β); 35.9 (Asp, C_β); 36.3 (Tyr, C_β); 50.8 (Asp, C_α); 55.9 (Tyr, C_α); 57.1 (Thr, C_β); 59.7 (Val, C_α); 60.4 (ASN$^{(Oxa)+}$, $C_{\delta'}$); 64.5 (ASN$^{(Oxa)+}$, $C_{\alpha'}$); 67.2 (Thr, C_α); 69.2 (ASN$^{(Oxa)+}$, C_β); 70.3 (ASN$^{(Oxa)+}$, C_α); 91.6 (ASN$^{(Oxa)+}$, C_δ); 115.7 (Tyr, C_ε); 128.5 (Tyr, C_γ); 130.8 (Tyr, C_δ); 156.2 (Tyr, C_ζ); 165.3 (ASN$^{(Oxa)+}$, CO); 171.3 (Asp, CO); 172.3 (Val, CO); 172.7 (Tyr, CO); 173.3 (Thr ,CO);173.7 (Asp, C_γO).

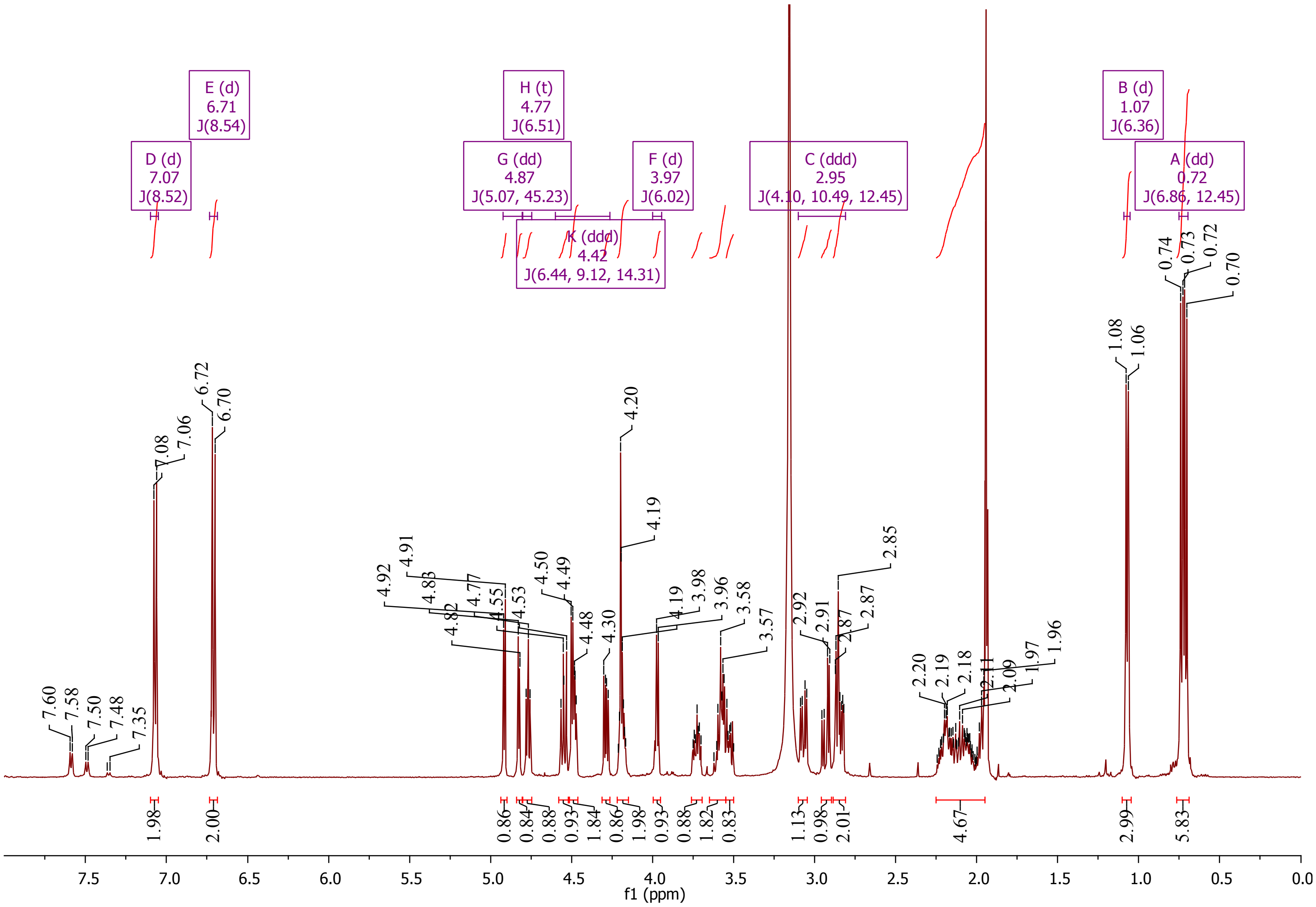

Fig. S64 ^{1}H NMR (600 MHz, CD_3CN, 300 K, σ) spectrum of the ASN^{Oxa+}–CO–DVYT–NH_2

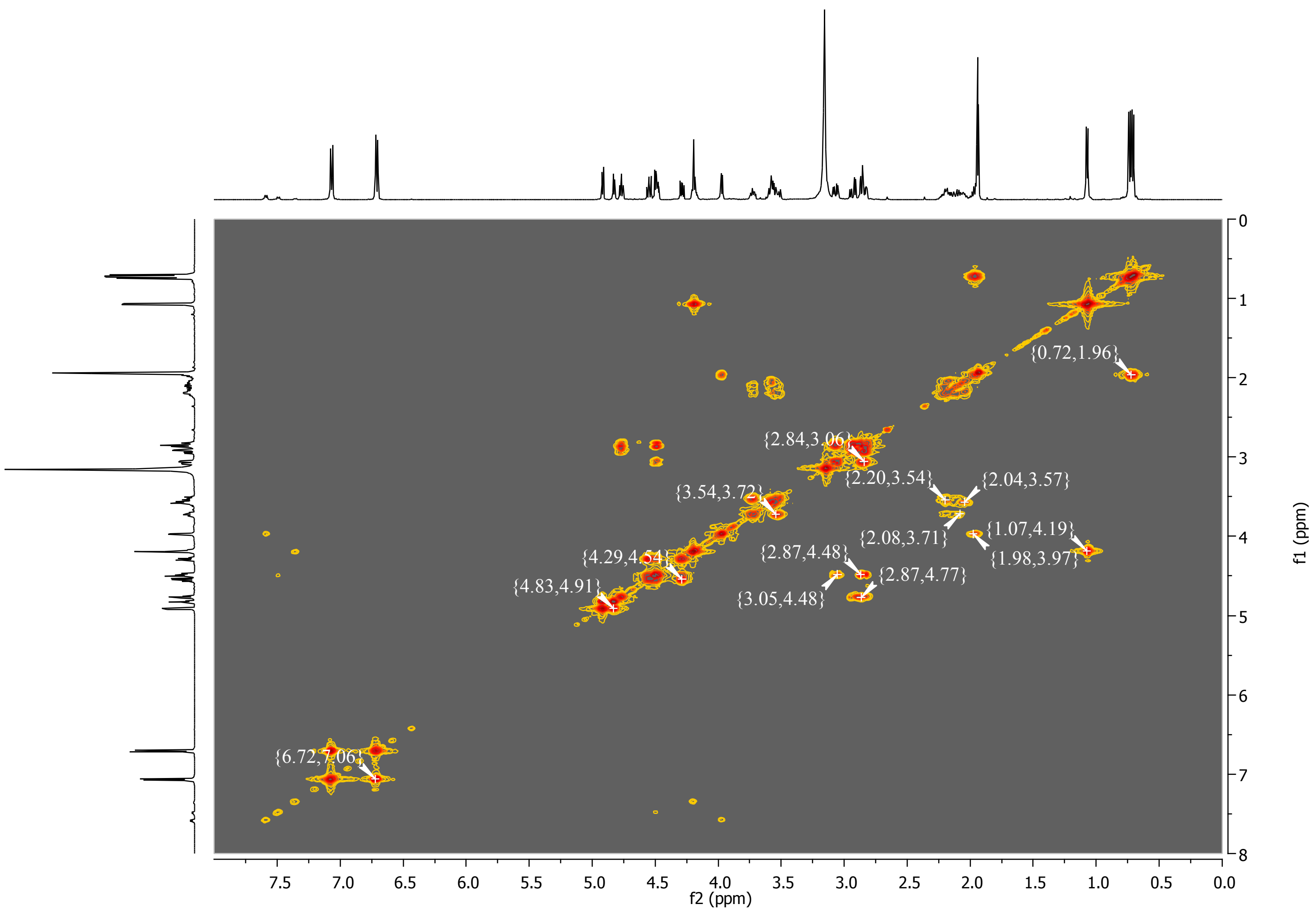

Fig. S65 COSY spectrum of the ASN^{Oxa+}–CO–DVYT–NH$_2$

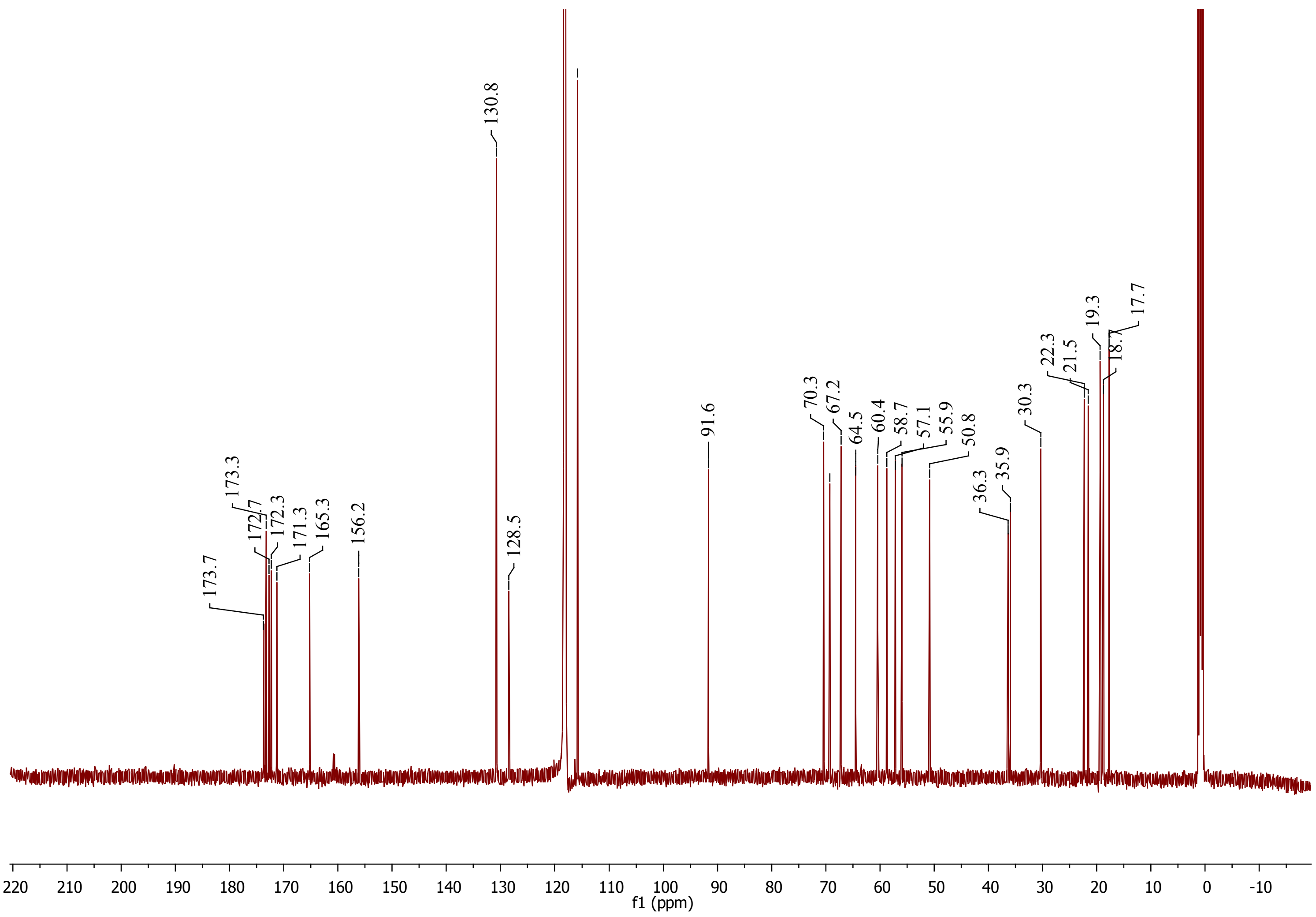

Fig. S66 ^{13}C NMR (150 MHz, CD_3CN, 300 K, σ) spectrum of the ASN^{Oxa+}–CO–DVYT–NH_2

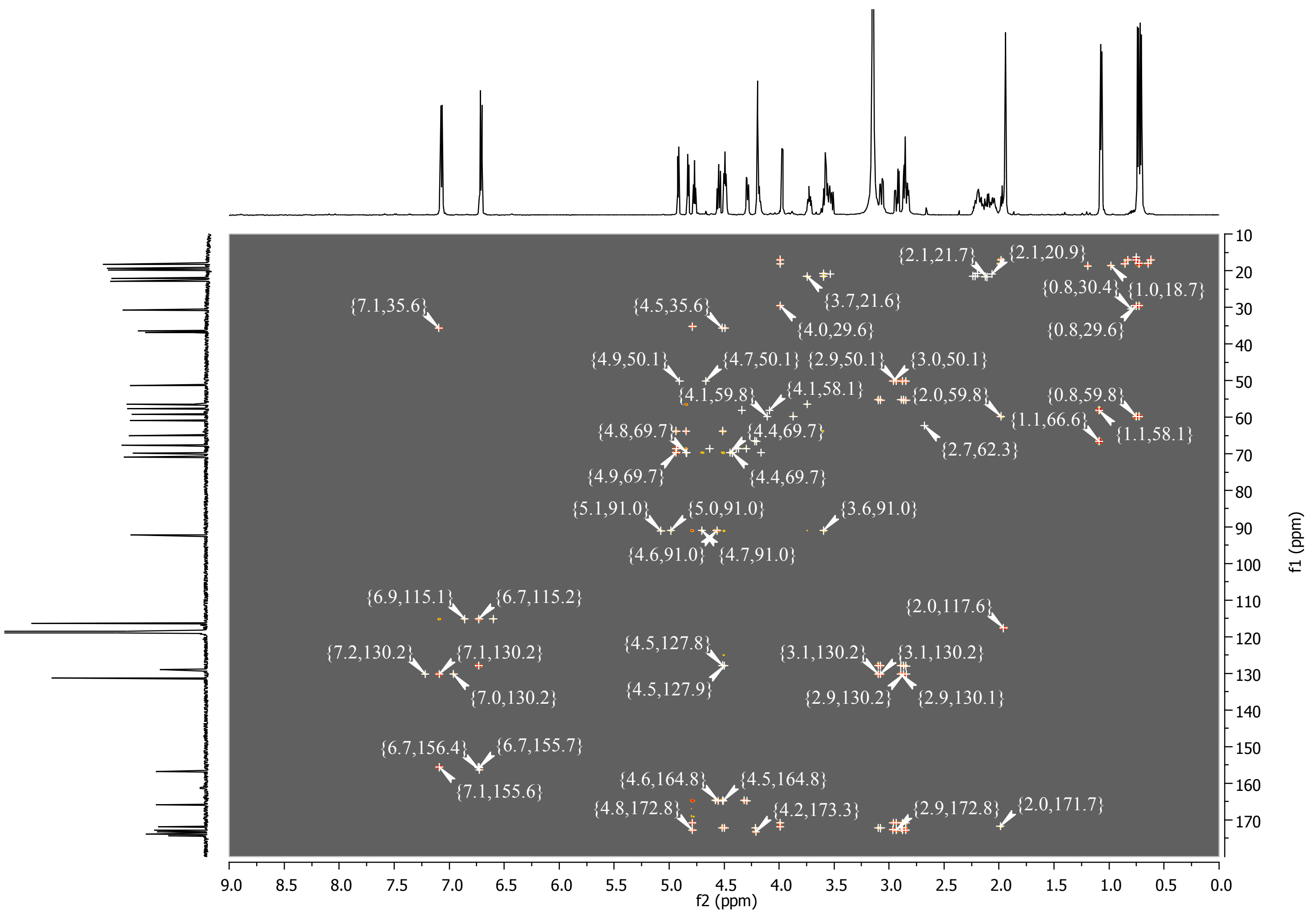

Fig. S67 HMBC spectrum of the ASN^{Oxa+}–CO–DVYT–NH$_2$

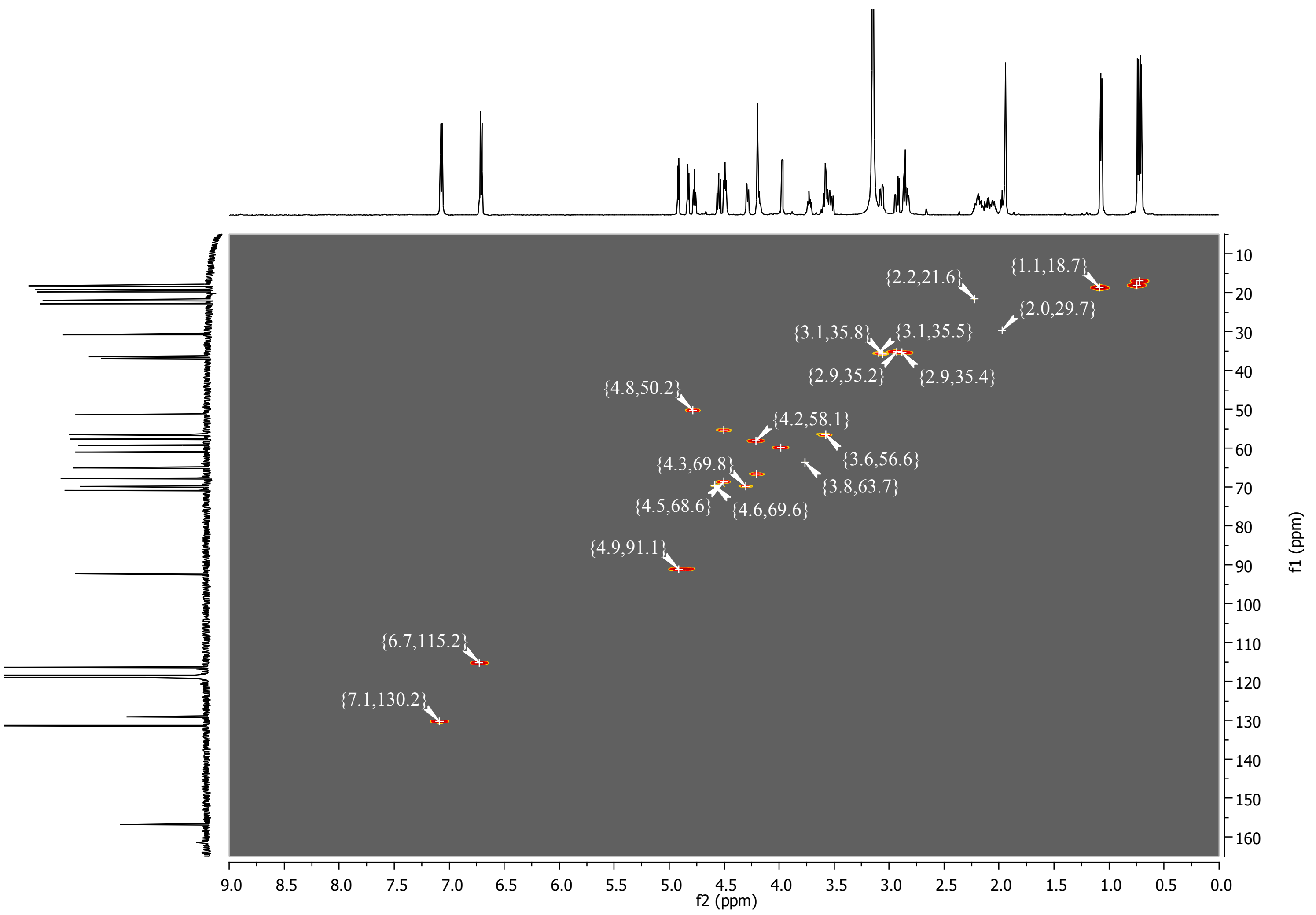

Fig. S68 HMQC spectrum of the ASN^{Oxa+}–CO–DVYT–NH_2

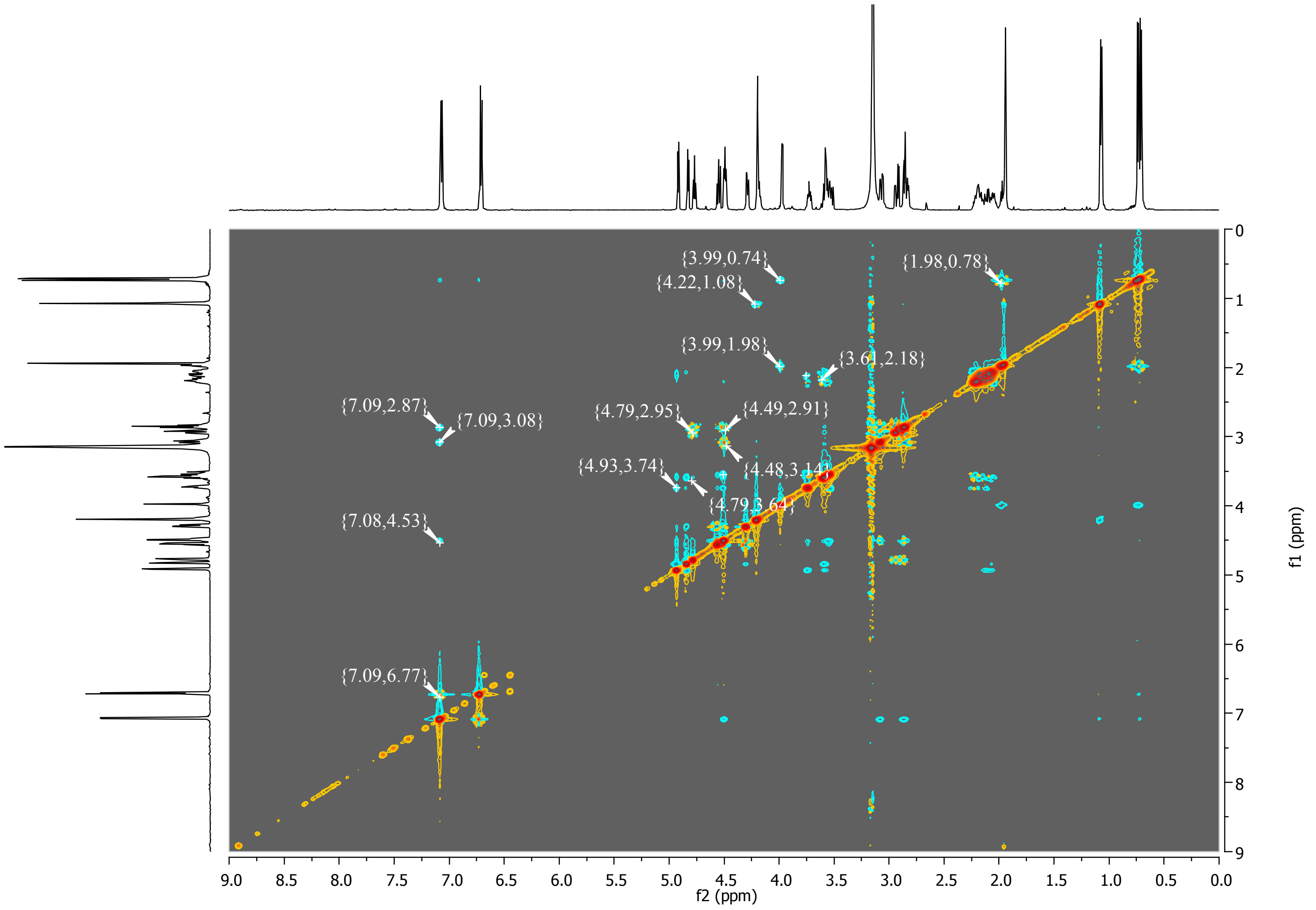

Fig. S69 NOESY spectrum of the ASN^{Oxa+}–CO–DVYT–NH$_2$

d. BASN^{Oxa+}–CO–DVYT–NH$_2$

^{1}H NMR (600 MHz, CD$_3$CN, 300 K) δ: 0.71 (dd, *J* = 6,9 Hz, 6H, Val, H$_\gamma$); 1.03 (d, J = 6,3 Hz, 3H, Thr, H$_\gamma$); 1.91–1.98 (m, 1H, Val, H$_\beta$); 2.69–2.86 (m, 3H, Asp, 2×H$_\beta$ and Tyr, 1×H$_\beta$); 3.04 (dd, *J* = 5,8 Hz, 1H, Tyr, H$_\beta$); 3.98 (d, *J* = 6,3 Hz, 1H, Val, H$_\alpha$); 4.14–4.18 (m, 2H, Thr, H$_\beta$, H$_\alpha$); 4.43 (ddd, *J* = 5,8 Hz, 2H, BASN^{Oxa+}, H$_\delta$); 4.66–5.06 (m, 9H, Asp, 1×H$_\alpha$, Tyr 1×H$_\alpha$ and BASN^{Oxa+}, 1×H$_\alpha$, 2×H$_\beta$, 2×H$_{\alpha'}$, 2×H$_{\delta'}$); 6.09–6.12 (m, 2H, Tyr, H$_\varepsilon$); 7.04–7.06 (m, 2H, Tyr, H$_\delta$); 7.38–7.44 (m, 4H, BASN^{Oxa+}, Ar^{o} and Ar^{m}).

^{13}C{H} NMR (150 MHz, CD$_3$CN, 300 K) δ: 18.2 (Val, C$_\gamma$); 19.3 (Val, C$_\gamma$); 19.8 (Thr, C$_\gamma$); 30.9 (Val, C$_\beta$); 36.3 (Asp, C$_\beta$); 36.9 (Tyr, C$_\beta$); 51.2 (Asp, C$_\alpha$); 56.4 (Tyr, C$_\alpha$); 59.3 (Thr, C$_\beta$); 60.8 (Val, C$_\alpha$); 60.8 (BASN^{Oxa+}, C$_{\delta'}$); 67.7 (Thr, C$_\alpha$); 69.0 (BASN^{Oxa+}, C$_{\alpha'}$); 69.9 (BASN^{Oxa+}, C$_\alpha$); 71.0 (BASN^{Oxa+}, C$_\delta$); 93.7 (BASN^{Oxa+}, C$_\beta$); 116.3 (Tyr, C$_\varepsilon$); 124.3 (BASN^{Oxa+}, Ar^{o}); 124.5 (BASN^{Oxa+}, Ar^{o}); 128.9 (Tyr, C$_\gamma$); 130.4 (BASN^{Oxa+}, Ar^{m}); 130.4 (BASN^{Oxa+}, Ar^{m}); 131.3 (Tyr, C$_\delta$); 133.0 (BASN^{Oxa+}, C$_{\beta'}$); 133.8 (BASN^{Oxa+}, C$_{\gamma'}$); 156.6 (Tyr, C$_\zeta$); 165.7 (BASN^{Oxa+}, CO); 171.9 (Asp, CO); 173.0 (Val, CO); 173.4 (Tyr, CO); 173.9 (Thr ,CO); 174.5 (Asp, C$_\gamma$O).

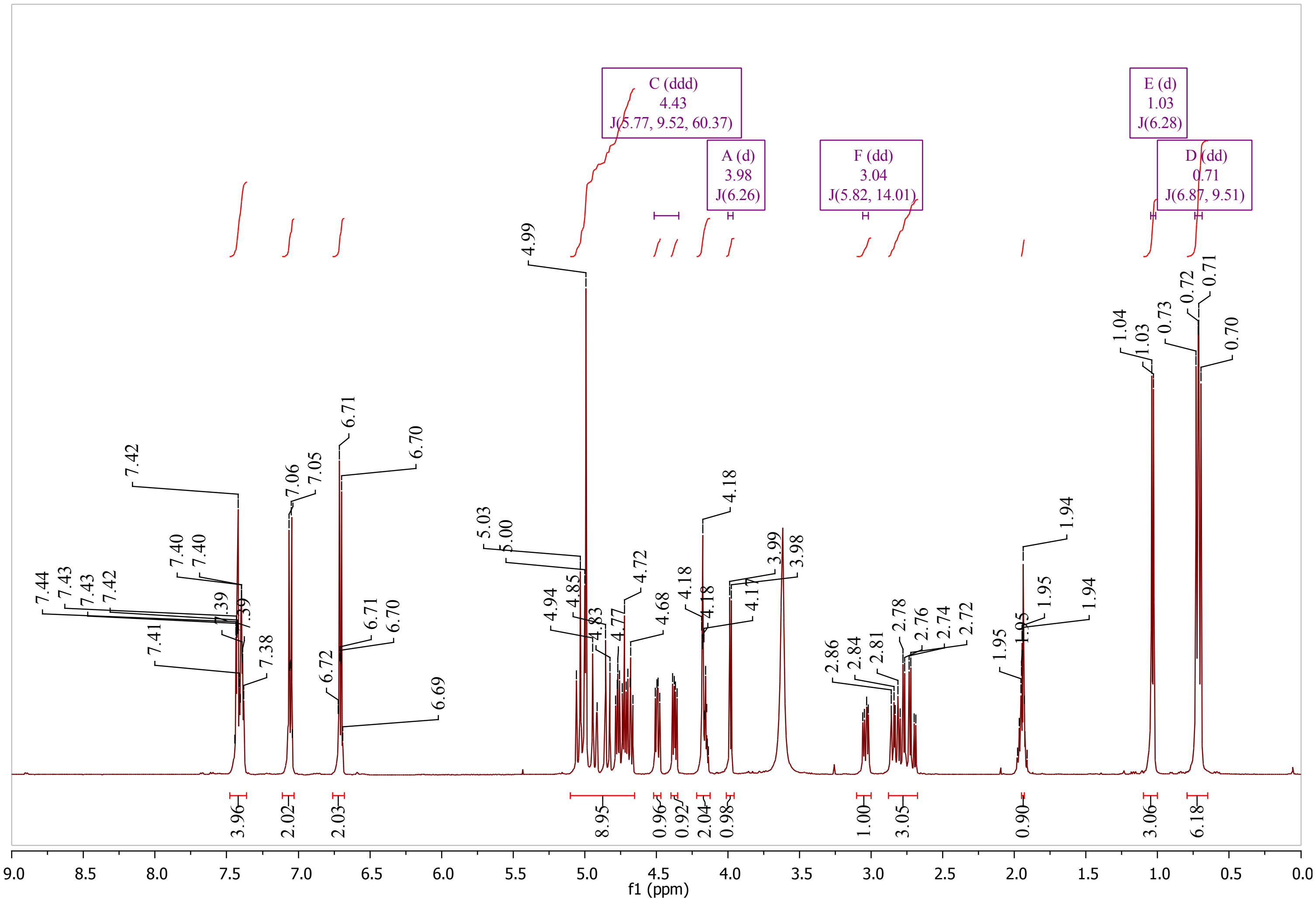

Fig. S70 NMR (600 MHz, CD_3CN, 300 K, σ) spectrum of the BASN^{Oxa+}–CO–DVYT–NH_2

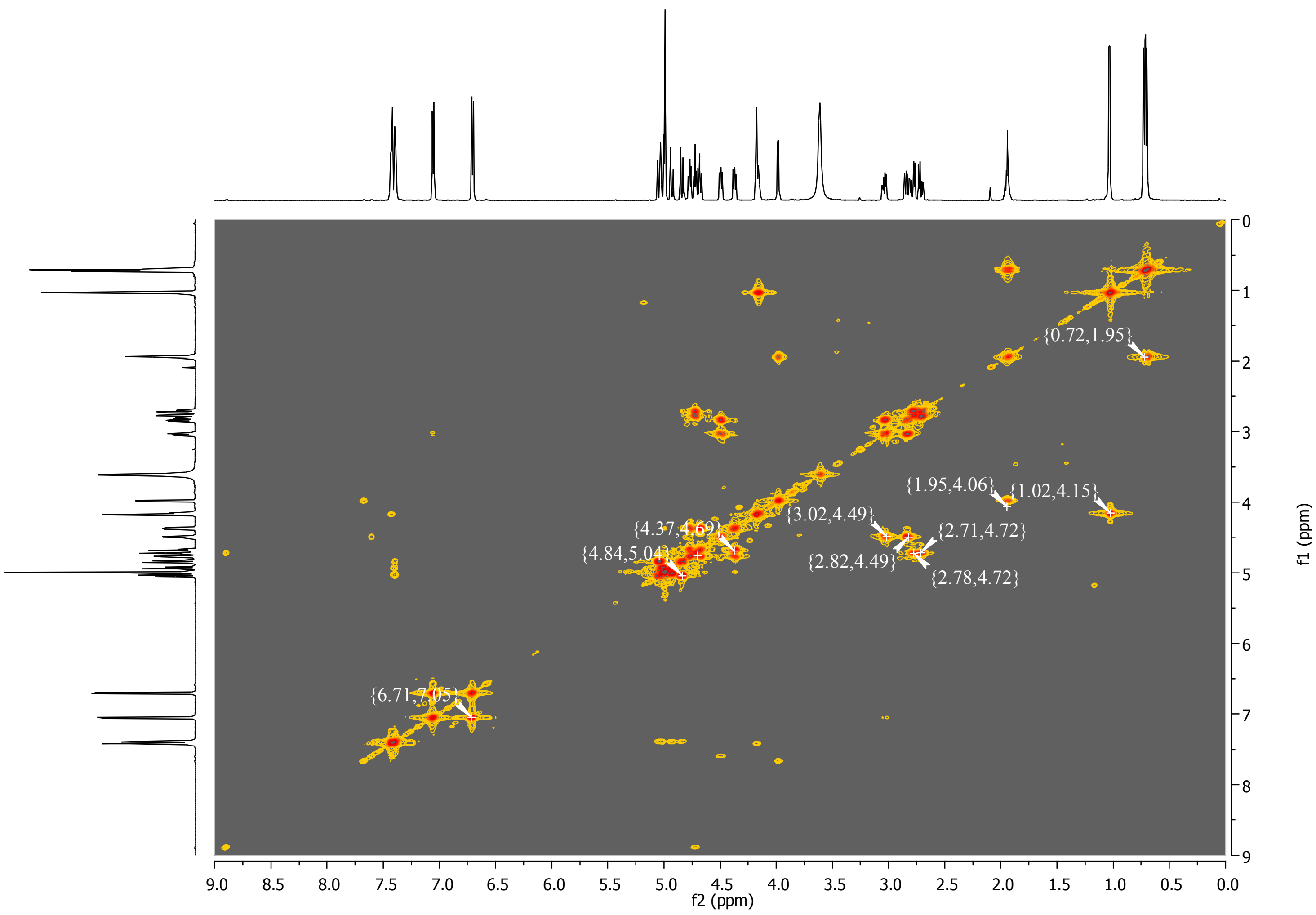

Fig. S71 COSY spectrum of the BASN^{Oxa+}–CO–DVYT–NH$_2$

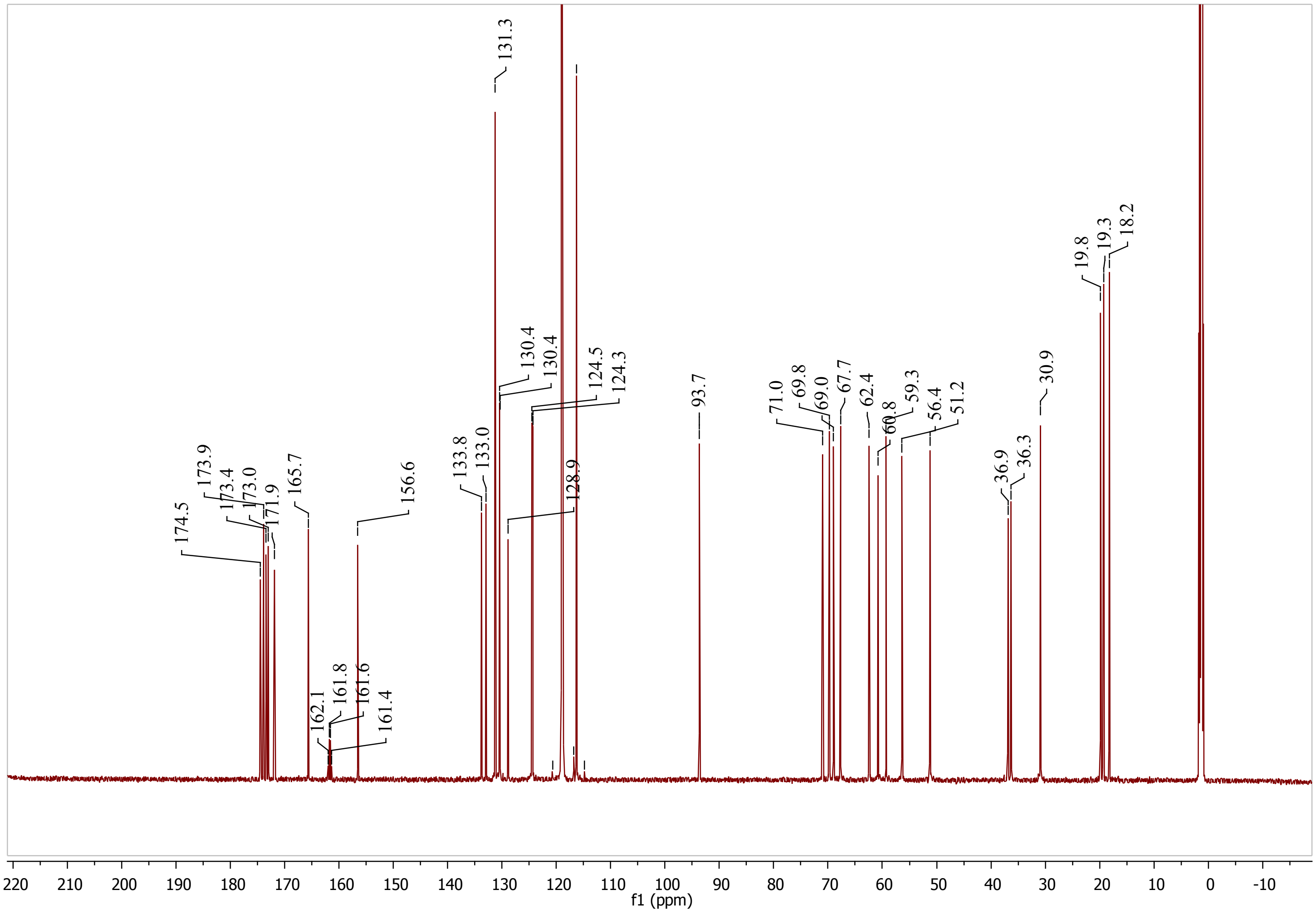

Fig. S72 ^{13}C NMR (150 MHz, CD_3CN, 300 K, σ) spectrum of the BASN^{Oxa+}–CO–DVYT–NH_2

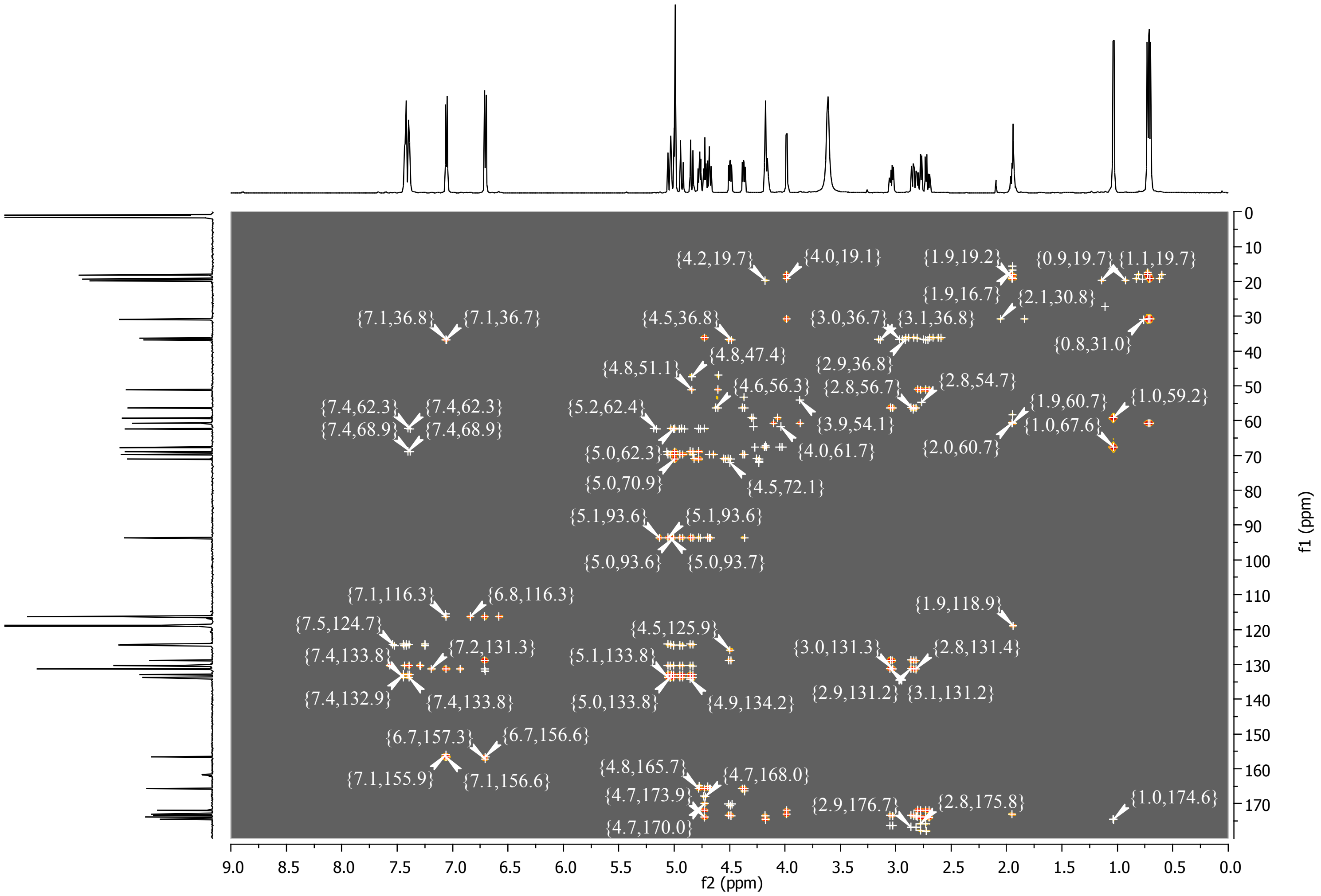

Fig. S73 HMBC spectrum of the BASN^{Oxa+}–CO–DVYT–NH$_2$

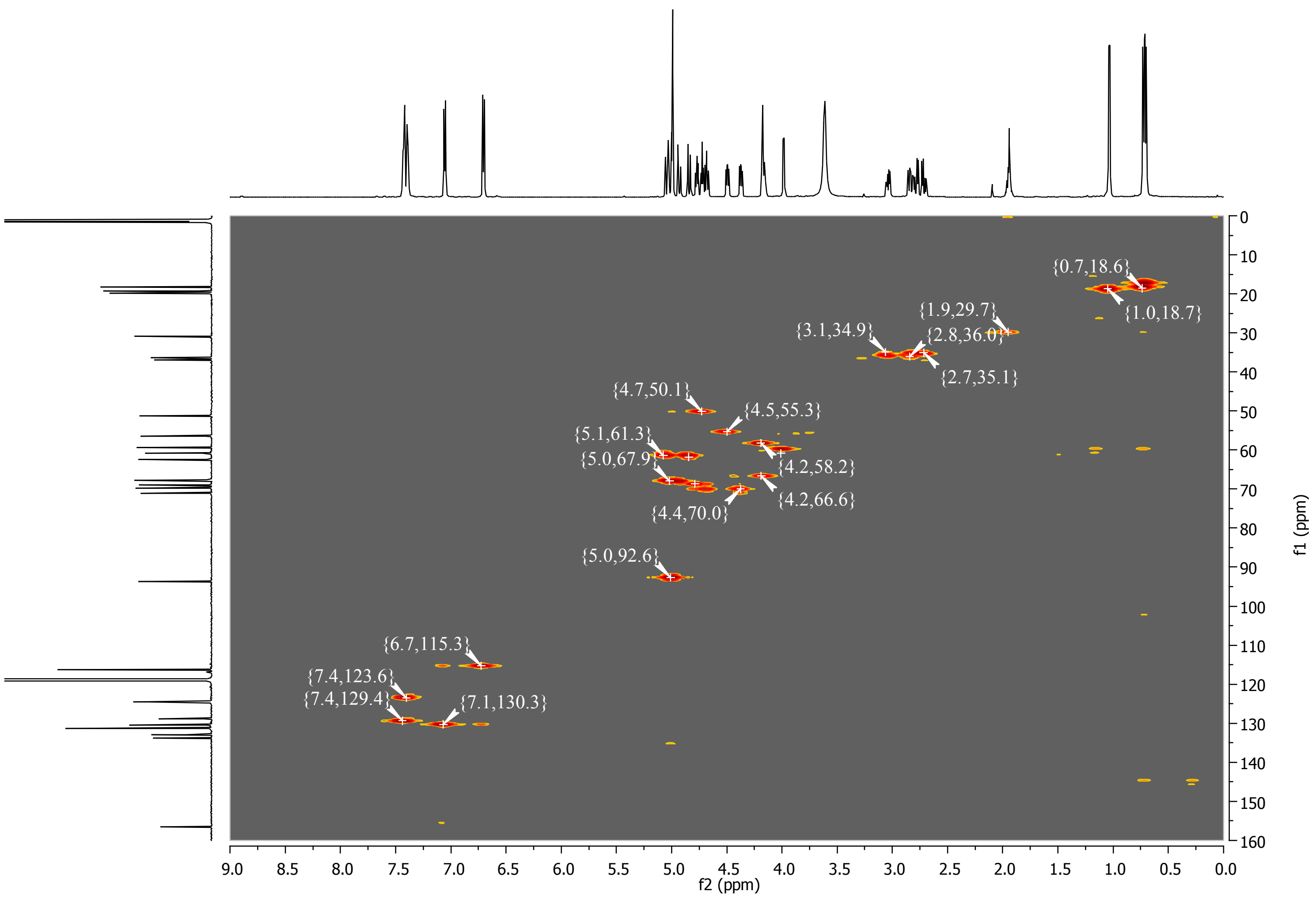

Fig. S74 HMQC spectrum of the BASN^{Oxa+}–CO–DVYT–NH_2

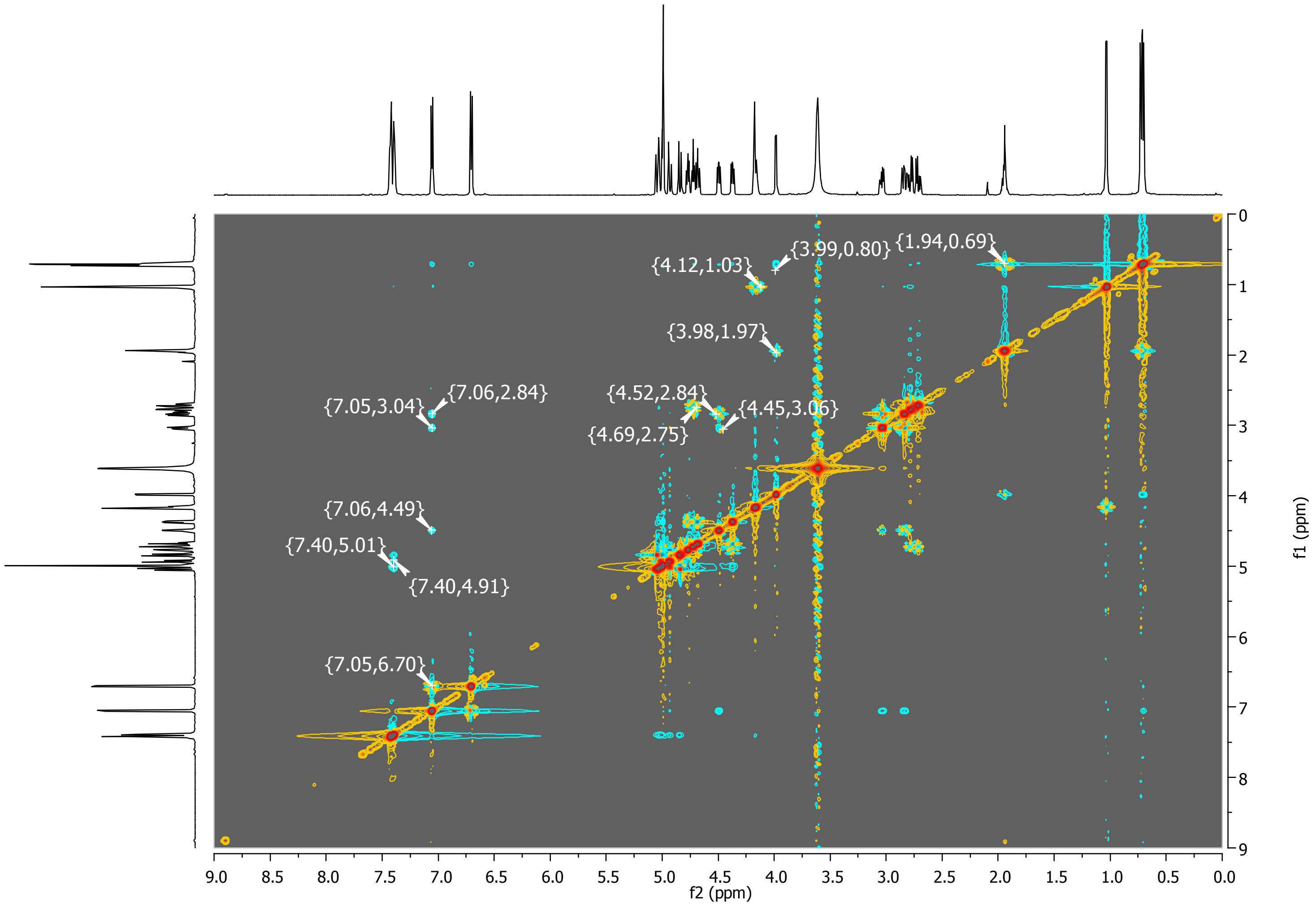

Fig. S75 NOESY spectrum of the BASN^{Oxa+}–CO–DVYT–NH_2

References

[1] M. Green, J. Berman. Preparation of pentafluorophenyl esters of FMOC protected amino acids with pentafluorophenyl trifluoroacetate. *Tetrahedron Lett.* **1990**, 31, 5851.

[2] H. Shaw, H. D. Perlmutter, C. Gu, S. A. Arco, T. O. Quibuyen. Free–Radical Bromination of Selected Organic Compounds in Water. *J. Org. Chem.* **1997**, 62, 236.

[3] M. Falorni, S. Conti, G. Giacomelli, S. Cossu, F. Soccolini. Optically Active 4-Oxaproline Derivatives. New Useful Chiral Synthons Derived from Serine and Threonine. *Tetrahedron*: *Asymmetry* **1995**, 6, 287.

[4] M. Rudowska, D. Wojewska, A. Kluczyk, R. Bąchor, P. Stefanowicz, Z. Szewczuk. The hydrogen-deuterium exchange at α-carbon atom in *N*,*N*,*N*-trialkylglycine residue: ESI-MS studies. *J. Am. Soc. Mass Spectrom.* **2012**, 23 , 1024.

[5] M. Wierzbicka, B. Setner, Z. Szewczuk. Analysis for trace amounts of analytes by electrospray mass spectrometry. *Acta Physica Polonica B Proceedings Supplement* **2016**, 9, 345.
